# The oncogene *AAMDC* links PI3K-AKT-mTOR signaling with metabolic reprograming in estrogen receptor-positive breast cancer

**DOI:** 10.1038/s41467-021-22101-7

**Published:** 2021-03-26

**Authors:** Emily Golden, Rabab Rashwan, Eleanor A. Woodward, Agustin Sgro, Edina Wang, Anabel Sorolla, Charlene Waryah, Wan Jun Tie, Elisabet Cuyàs, Magdalena Ratajska, Iwona Kardaś, Piotr Kozlowski, Elizabeth K. M. Johnstone, Heng B. See, Ciara Duffy, Jeremy Parry, Kim A. Lagerborg, Piotr Czapiewski, Javier A. Menendez, Adam Gorczyński, Bartosz Wasag, Kevin D. G. Pfleger, Christina Curtis, Bum-Kyu Lee, Jonghwan Kim, Joseph Cursons, Nathan J. Pavlos, Wojciech Biernat, Mohit Jain, Andrew J. Woo, Andrew Redfern, Pilar Blancafort

**Affiliations:** 1 Cancer Epigenetics Group, The Harry Perkins Institute of Medical Research, The University of Western Australia, Perth, WA Australia; 2 Centre for Medical Research, The University of Western Australia, Perth, WA Australia; 3 Department of Microbiology and Immunology, Faculty of Medicine, Minia University, Minia, Egypt; 4 School of Human Sciences, The University of Western Australia, Perth, WA Australia; 5 Girona Biomedical Research Institute, Girona, Catalonia Spain; 6 ProCURE (Program Against Cancer Therapeutic Resistance), Metabolism & Cancer Group, Catalan Institute of Oncology, Girona, Catalonia Spain; 7 Department of Biology and Medical Genetics, Medical University of Gdansk, Gdansk, Poland; 8 The Centre for Cell Therapy and Regenerative Medicine, School of Biomedical Sciences, The University of Western Australia, Perth, WA Australia; 9 Department of Pathology, Otago University, Dunedin, New Zealand; 10 Laboratory of Clinical Genetics, University Clinical Centre, Gdansk, Poland; 11 Institute of Bioorganic Chemistry, Polish Academy of Sciences, Poznan, Poland; 12 Molecular Endocrinology and Pharmacology, Harry Perkins Institute of Medical Research, Nedlands, WA Australia; 13 Australian Research Council Centre for Personalised Therapeutics Technologies, Melbourne and Perth, Australia; 14 Department of Anatomical Pathology, Path West Laboratory, Fiona Stanley Hospital Network, Murdoch, WA Australia; 15 Departments of Medicine and Pharmacology, University of California, San Diego, CA USA; 16 Department of Pathomorphology, Medical University of Gdansk, Gdansk, Poland; 17 Institute of Pathology, Dessau Medical Centre, Dessau, Germany; 18 Dimerix Limited, Nedlands, WA Australia; 19 Stanford University School of Medicine (Departments of Medicine & Genetics) and Stanford Cancer Institute, Stanford, CA USA; 20 Department of Biomedical Sciences, Cancer Research Center, University at Albany-State University of New York, Rensselaer, NY USA; 21 Department of Molecular Biosciences, Center for Systems and Synthetic Biology, The University of Texas at Austin, Austin, TX USA; 22 Biomedicine Discovery Institute & Department of Biochemistry and Molecular Biology, Monash University, Clayton, VIC Australia; 23 School of Biomedical Sciences, The University of Western Australia, Perth, WA Australia; 24 School of Medical and Health Sciences, Edith Cowan University, Perth, WA Australia; 25 School of Medicine, University of Western Australia, Perth, WA Australia; 26 The Greehey Children’s Cancer Research Institute, The University of Texas Health Science Center at San Antonio, San Antonio, TX USA

**Keywords:** Breast cancer, Cancer metabolism

## Abstract

*Adipogenesis associated Mth938 domain containing (AAMDC)* represents an uncharacterized oncogene amplified in aggressive estrogen receptor-positive breast cancers. We uncover that *AAMDC* regulates the expression of several metabolic enzymes involved in the one-carbon folate and methionine cycles, and lipid metabolism. We show that *AAMDC* controls PI3K-AKT-mTOR signaling, regulating the translation of ATF4 and MYC and modulating the transcriptional activity of *AAMDC*-dependent promoters. High *AAMDC* expression is associated with sensitization to dactolisib and everolimus, and these PI3K-mTOR inhibitors exhibit synergistic interactions with anti-estrogens in IntClust2 models. Ectopic *AAMDC* expression is sufficient to activate AKT signaling, resulting in estrogen-independent tumor growth. Thus, *AAMDC*-overexpressing tumors may be sensitive to PI3K-mTORC1 blockers in combination with anti-estrogens. Lastly, we provide evidence that AAMDC can interact with the RabGTPase-activating protein RabGAP1L, and that AAMDC, RabGAP1L, and Rab7a colocalize in endolysosomes. The discovery of the RabGAP1L-AAMDC assembly platform provides insights for the design of selective blockers to target malignancies having the *AAMDC* amplification.

## Abstract (web summary)

Adipogenesis associated Mth938 Domain Containing gene (AAMDC) is frequently amplified in the IntClus2 subgroup of ER + breast cancer. Here, the authors show that AAMDC drives tumourigenesis through activating PI3K-AKT-mTOR pathway for metabolic reprogramming.

## Introduction

Analysis of transcriptomic data has traditionally stratified breast cancers (BCs) into six subtypes: hormone receptor-positive (HR^+^) luminal A and luminal B, HER2-enriched (HER2^+^), basal-like, claudin-low, and normal-like^1,2^. While luminal A tumors generally respond well to anti-hormonal therapies, luminal B tumors often lose HR-driven transcriptional programs and have higher rates of mortality^3^. To date, there are no clinical biomarkers to stratify the fraction of HR^+^ tumors with a high probability of relapse from the bulk of malignancies that respond to traditional anti-hormonal approaches^3^. Current diagnostic tests, such as Oncotype Dx, are limited to the determination of whether the addition of cytotoxic chemotherapy can provide a therapeutic benefit, rather than identifying rational drug targets to directly inhibit oncogenic drivers^4^.

More recent studies integrating both transcriptomic and genomic datasets have provided further insights into breast cancer heterogeneity by defining 10 different integrative clusters (IntClust) subtypes. A subgroup of estrogen receptor-positive (ER^+^) cancers (IntClust2) has been identified with a prognosis inferior to all but the HER2-enriched group (IntClust5). The hallmark of IntClust2 tumors is focal amplification of a region of chromosome 11 (chr 11) (11q13.5–14.1) defining a *cis*-acting oncogenic cluster^5^. This subtype harbors genomic and transcriptional alterations in cell cycle-related genes, including the *MYC* targets *CCND1* and *CCNE1*, and displays aggressive behavior including a high level of proliferation and endocrine therapy resistance^5^. The 11q13.5–14.1 amplicon encompasses 12 potential oncogenes, two with recognized roles in anti-estrogen and paclitaxel resistance in breast and ovarian cancer: *RSF1*^6^ and *PAK1*^7^, and at least three targets in the amplicon are known to regulate metabolic processes: *NDUFC2*^8^, *ALG8*^9^, and *THRSP*^10^. Importantly, *AAMDC* (*Adipogenesis associated Mth938 domain containing*), is centered in the peak of the amplicon, and it thus represents one of the most frequently amplified genes in the cluster, highlighting a prospective biomarker for IntClust2 subtype identification as well as a potential pathogenic driver for these cancers.

*AAMDC* encodes a 122 amino acid protein of unknown function that has a high degree of structural homology with a bacterial protein from *M. thermoautotrophicum*, thus suggesting collateral gene transfer from bacteria to eukaryotic cells^11^. The induction of adipogenesis upon overexpression of the murine homolog of *AAMDC* in 3T3-L1 pre-adipocyte cells^12^, together with its observed pattern of mRNA expression in white adipose tissue, suggests a potential role for this gene in metabolism. However, the distribution and function of this putative protein in human cancers has not been previously investigated.

Our results indicate that AAMDC is a signal transduction oncoprotein that constitutively activates the PI3K-AKT-mTOR pathway, thereby inducing survival of ER^+^ BCs during metabolic stress conditions such as estrogen deprivation. Our work suggests that the proliferation and survival of IntClust2 malignancies may be dependent on metabolic reprograming, and hence these poor prognosis ER^+^ tumors could be vulnerable to tailored therapies focusing on PI3K-mTOR inhibitors in combination with anti-estrogens. The discovery of potential binding interfaces between AAMDC and RabGAP1L (Rab-GTPase activating protein) could assist in the development of inhibitors to target these currently hard-to-drug proteins. We propose that the identification of *AAMDC*-overexpressing tumors could facilitate the identification of patients with a poor prognosis and the development of selective therapeutic interventions against this aggressive subtype of ER^+^ disease.

## Results

### Overexpression and amplification of *AAMDC* in ER^+^ BCs

**Fig. 1:**
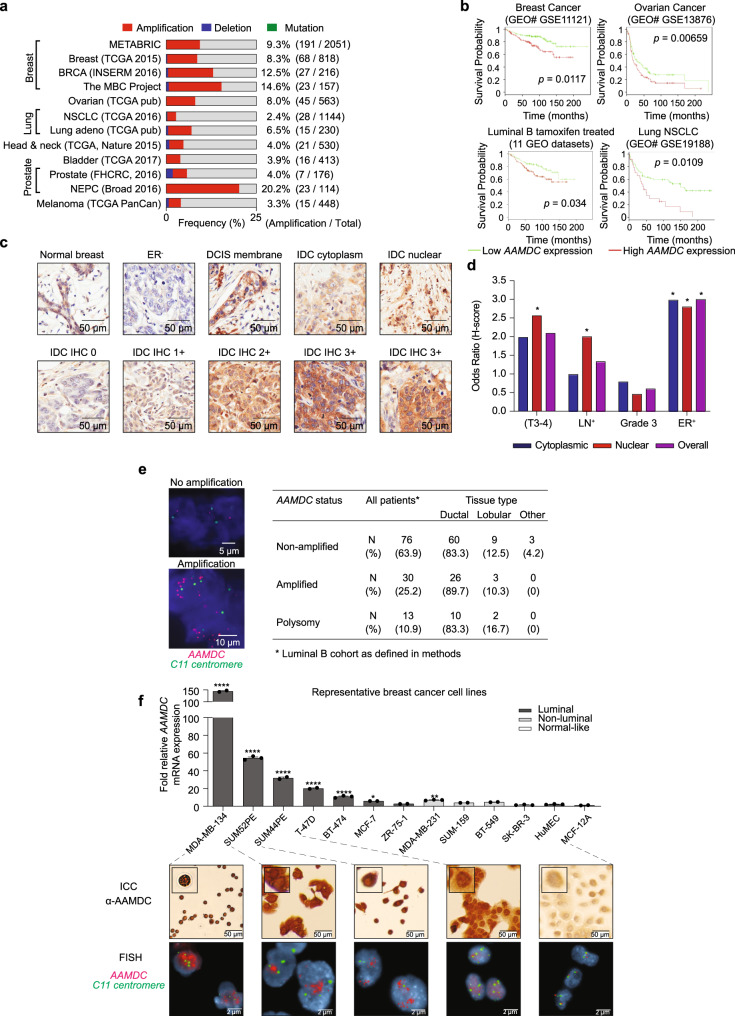
*AAMDC* overexpression and amplification are associated with a subgroup of ER^+^ breast cancer with poor prognosis. **a** Analysis of somatic alterations of *AAMDC* using cancer genomic data sets and tools available from cBioPortal (see “Methods”). The frequency of amplification is shown as a percentage and the sample numbers are shown in brackets. METABRIC Molecular Taxonomy of Breast Cancer International Consortium, TCGA The Cancer Genome Atlas, BRCA Breast Cancer, INSERM Institut national de la santé et de la recherche médicale, MBC Metastatic Breast Cancer, NSCLC non-small-cell lung carcinoma, FHCRC Fred Hutchinson Cancer Research Center, NEPC National Environment Protection Council, PanCan Pan-Cancer. **b** Kaplan–Meier survival plots for patients with tumors expressing high (red) or low (green) levels of *AAMDC* mRNA. The lower left plots correspond to luminal B tumors treated with tamoxifen (see “Methods”). The *p* value shown for each plot is determined by the log-rank test. GEO Gene Expression Omnibus, GSE genomic spatial event, NSCLC non-small-cell lung carcinoma. **c** Localization of the AAMDC protein in tumors from a breast tissue microarray (TMA) assessed by immunohistochemistry (IHC). Representative IHC sections of normal breast tissue, estrogen receptor-negative (ER^−^) tumor tissue, ductal carcinoma in situ (DCIS), and invasive ductal carcinoma (IDC) are shown. 0, 1+, 2+, 3+ indicate the staining intensity score. **d** Associations between AAMDC expression (IHC) and lymph node metastasis (LN^+^) as well as tumor grade, tumor size (T3-4), and ER positivity (ER^+^) by AAMDC localization from the same TMA. Statistical significance is indicated by Chi-square analysis with a one-tailed *p*-value relative to ER^−^ tissue. For T3-4: *p = 0.03; for LN^+^: *p = 0.03; for ER^+^, from left to right: *p = 0.003, *p = 0.005, *p = 0.005. *n* = 60 biologically independent samples. Full details of the TMA are provided in Supplementary Table 1. **e** Frequency of *AAMDC* amplification/polysomy in a cohort of 119 luminal B breast cancer specimens. Representative fluorescence in situ hybridization (FISH) images are indicated, with specific probes for *AAMDC* (red) and *Centromere enumeration 11* probe for *chromosome 11* (*C11*, green). The full clinical and pathological features of these tumors are shown in Supplementary Data 1. **f** Real-time expression analyses (qRT-PCR) of *AAMDC* in luminal, non-luminal, and normal-like breast cells. Significance levels are determined relative to MCF-12A by Ordinary one-way ANOVA with Dunnett multiple comparison test. Data are presented as mean values ± SEM (*p = 0.0217, **p = 0.0018, ****p < 0.0001). *n* = 3 biologically independent RNA extractions. Representative images of immunocytochemistry (ICC) and FISH of selected luminal cell lines are presented. HuMECs non-transformed human mammary epithelial cells.

Examination of oncogenomic databases revealed frequent *AAMDC* copy number alterations in a broad array of patient samples, including breast, ovarian, lung, and prostate cancers (Fig. 1a). These were predominantly amplification alterations, with infrequent gene mutation or deletion events, and a frequency of amplification of ~10% of BC cases across multiple databases (Fig. 1a). Tumors with high *AAMDC* expression had inferior overall survival in breast, ovarian, and lung cancers. In addition, high expression of *AAMDC* significantly correlated with lower survival in aggressive luminal B BCs treated with tamoxifen, thus suggesting an association with anti-estrogen therapy resistance (Fig. 1b).

To explore the demographics of AAMDC expression, we performed immunohistochemistry (IHC, Fig. 1c) in a breast tissue microarray (Biomax) including normal breast tissue, benign breast lesions, and 60 high-risk BC cases (Supplementary Fig. 1a and b for validation of the α-AAMDC antibody). AAMDC, as quantified by the H-index (intensity × % positive cells), was basally expressed at lower levels in normal and non-luminal cells and at higher levels in in situ carcinoma. As expected from genomic databases, the antigen was selectively and significantly overexpressed in ER^+^ relative to ER^−^ breast tumors (47 vs. 15%, *χ*^2^ *p* = 0.005; Supplementary Table 1). AAMDC^+^ cancers exhibited the highest expression in the cytoplasm, with relatively frequent but a lower level of nuclear and membrane-associated staining (Fig. 1c). Even within this high-risk patient series, tumors with a high level of nuclear expression showed more frequent lymph node involvement (48 vs. 24%, *p* = 0.03) and an increase in the proportion of tumors >5 cm (36 vs. 14%, *p* = 0.03). Interestingly, in contrast to the nuclear staining, these correlations were not significant for the cytoplasmic staining (Fig. 1d, Supplementary Table 1).

To validate the frequency of *AAMDC* amplification in high-risk HR^+^ BCs, fluorescence in situ hybridization (FISH) was conducted on a focused cohort of 119 luminal B BCs using probes for *AAMDC* and *Centromere enumeration 11* (*CEN-11*). Notably, 25% of the luminal B tumors screened exhibited *AAMDC* amplification, with amplification indexes from >2–5, and a further 11% displayed a chr 11 polysomy, with a trend of the amplification correlating with lymph node involvement (Fig. 1e, Supplementary Data 1). Although AAMDC expression and amplification did not correlate with HER2 positivity, they were not mutually exclusive to this such that both amplifications could exist within a single tumor (Supplementary Table 1 and Supplementary Data 1).

Consistent with the clinical data, the overexpression and amplification of *AAMDC* were predominant in ER^+^ luminal cell lines, with the highest level of expression in MDA-MB-134, SUM52PE, and SUM44PE, all of which harbored the amplification, followed by T-47D, which presented a chr 11 polysomy. The lowest expression of *AAMDC* was detected in ER^−^ tumor cell lines and in non-transformed primary human mammary epithelial cells (HuMEC) and MCF-12A cells. AAMDC was found in both cytoplasmic and nuclear compartments, accompanied by plasma membrane staining depending on the cell line analyzed (Fig. 1f).

### Depletion of *AAMDC* expression inhibits BC cell growth and migration

**Fig. 2:**
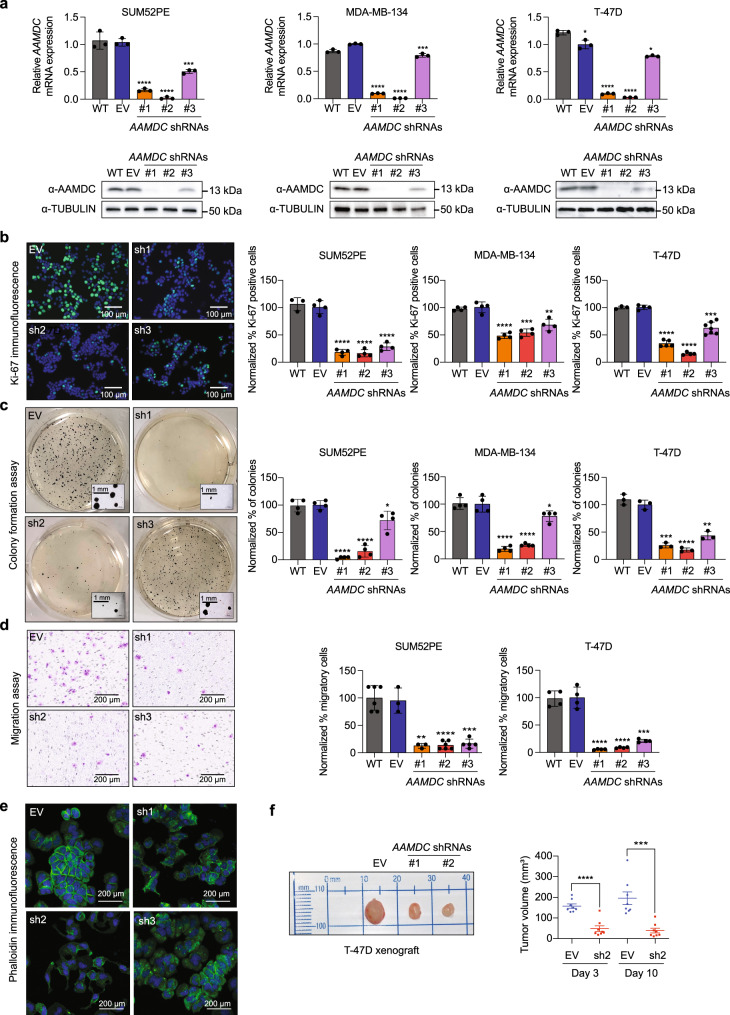
*AAMDC* knockdown in luminal breast cancer cells inhibits tumorigenic and migratory capacity. **a** Detection of *AAMDC* expression in luminal breast cancer cells with the *AAMDC* amplification (SUM52PE and MDA-MB-134) or the chromosome 11 polysomy (T-47D). Cells were transduced with either the *AAMDC* shRNAs #1–#3 or with empty vector (EV) and processed by qRT-PCR (top) and immunoblotting (bottom). Wild-type (WT) indicates untransduced cells. Data are normalized to the EV control and presented as mean values ± SD. *p*-values are determined by two-tailed unpaired *t*-test (SUM52PE: ***p = 0.0003, ****p < 0.0001; MDA-MB-134: ***p = 0.0004, ****p < 0.0001; T-47D: *p = 0.0142 and *p = 0.0107 for EV and shRNA #3 respectively, ****p < 0.0001). *n* = 3 biologically independent experiments. Source data are provided as a Source Data file. **b** Cell proliferation assessed by α-Ki-67 immunostaining (green), superimposed on nuclear Hoechst 33258 staining (blue). Data are normalized to the EV and presented as mean values ± SD. *p*-values are determined by two-tailed unpaired *t*-test (SUM52PE: ****p < 0.0001; MDA-MB-134: **p = 0.0044, ***p = 0.0002, ****p < 0.0001; T-47D: ***p = 0.0001, ****p < 0.0001). *n* = 3 biologically independent experiments. **c** Inhibition of anchorage-independent cell growth by depletion of *AAMDC* expression assessed by soft agar colony formation assay 28 days after transduction. Data are normalized to the EV and presented as mean values ± SD. *p*-values are determined by two-tailed unpaired *t*-test (SUM52PE: *p = 0.0235, ****p < 0.0001; MDA-MB-134: *p = 0.0466, ****p < 0.0001; T-47D: **p = 0.0010, ***p = 0.0002, ****p < 0.0001). *n* = 3 biologically independent experiments. **d** Inhibition of cell migration as assessed by Boyden migration chamber assays 24 h after transduction. Data are normalized to the EV and presented as mean values ± SD. *p*-values are determined by two-tailed unpaired *t*-test (SUM52PE: **p = 0.0036, ***p = 0.0004, ****p < 0.0001; T-47D: ***p = 0.0002, ****p < 0.0001). *n* = 3 biologically independent experiments. **e** Effect of the *AAMDC* knockdown (KD) on F-actin organization assessed by phalloidin fluorescence staining (Alexa Fluor 488, green), nuclei (Hoechst 33258, blue). In (**b**–**e**), representative images of SUM52PE cells transduced with EV or *AAMDC* shRNAs #1–#3 (sh1–3) are shown (left). In (**a**–**d**), quantification for each cell line transduced with empty vector (EV, blue), shRNA #1 (orange), shRNA #2 (red), shRNA #3 (lilac), and untransduced (WT, gray). **f** Tumor growth inhibition in vivo by *AAMDC* KD in the T-47D xenograft model in nude mice. Representative sizes of the tumors extracted from the animals at day 10 post-injection of the cells are shown (left). Scatter dot plot outlining the decrease in tumor volume at day 3 and day 10 post-implantation of the cells (right). EV, blue; sh2, red. Data are normalized to the EV and presented as mean values ± SEM. *p*-values are determined by two-tailed unpaired *t*-test (***p = 0.0003, ****p < 0.0001), *n* = 8 mice per group.

We investigated the role of *AAMDC* by lentiviral transduction of three shRNAs in cell lines expressing high *AAMDC* levels associated with the *AAMDC* amplification (MDA-MB-134 and SUM52PE), or moderate levels (T-47D) associated with the chr 11 polysomy (Fig. 2a). The on-target specificity of the most potent shRNA utilized in genomic and functional studies (*AAMDC* sh2) was validated by *AAMDC* cDNA rescue experiments (Supplementary Fig. 1c). The *AAMDC* knockdowns (KDs) inhibited cell proliferation (Fig. 2b) and impaired colony formation in vitro (Fig. 2c), accompanied by a small but significant increase in apoptosis (Supplementary Fig. 1e). Furthermore, the *AAMDC* KDs reduced cell migration (Fig. 2d), a finding consistent with altered F-actin organization (Fig. 2e) and significantly decreased xenograft tumor burden in mice (Fig. 2f). Analysis of DepMap CRISPR knockout screening data matched with available molecular data from the Cancer Cell Line Encyclopedia (CCLE) provided independent confirmation that the SUM52PE cell line with strong amplification and *AAMDC* overexpression had a greater dependence on *AAMDC* for cell survival relative to other luminal BC lines that do not harbor the *AAMDC* amplification (Supplementary Fig. 1d).

In non-transformed mammary epithelial cells (HuMECs), even the most potent *AAMDC* KDs produced only minor changes in cell proliferation compared to the changes in cancerous IntClust2 cell lines, thus suggesting that IntClust2 cells are more dependent on *AAMDC* expression (Supplementary Fig. 2a). However, *AAMDC* cDNA overexpression did not activate cell migration in poorly migratory cells (Supplementary Fig. 2b) nor did it induce anchorage independence in HuMEC and primary adult human dermal fibroblasts (HDFa) (Supplementary Fig. 2c).

Since bovine and murine pre-adipocytes can be differentiated to induce lipid production by *AAMDC* cDNA overexpression^12,13^, we also investigated whether *AAMDC* could regulate lipogenesis in human cells. In SUM52PE cells, the *AAMDC* KD significantly reduced the percentage of Oil Red O^+^ cells, thus suggesting that *AAMDC* may control the accumulation of neutral lipids. In contrast, the *AAMDC* cDNA did not significantly induce lipogenesis in HuMECs (Supplementary Fig. 2d).

### *AAMDC* controls transcription of multiple metabolic enzymes

**Fig. 3:**
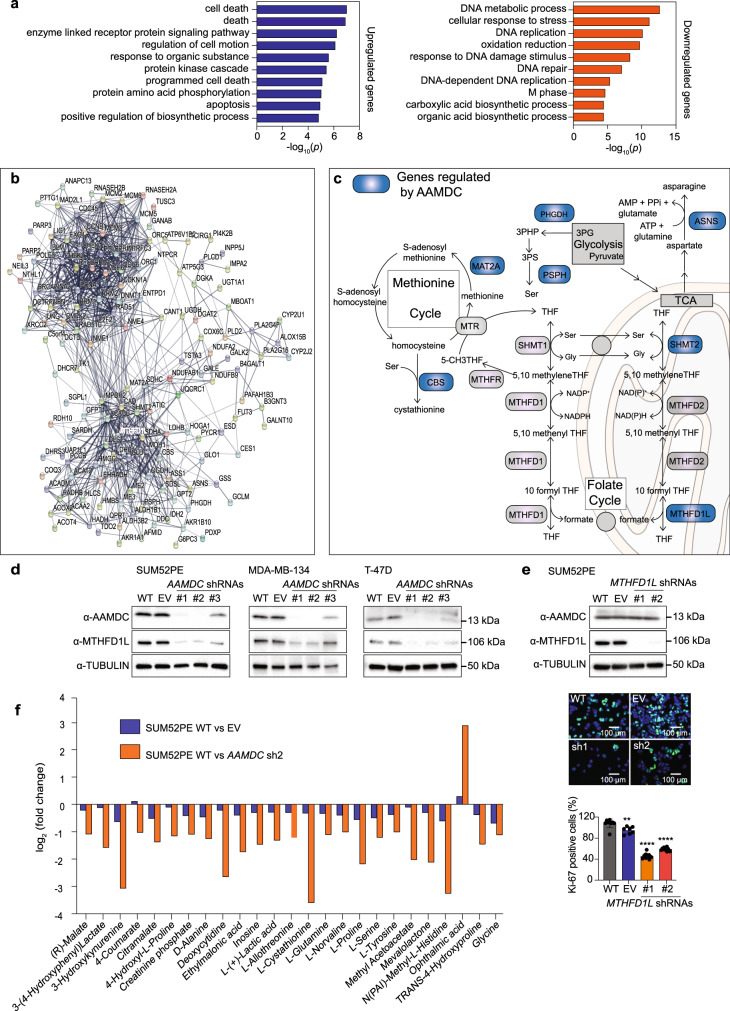
*AAMDC* regulates targets involved in cell proliferation and metabolism. **a** Gene ontology (GO) analyses of the top 10 biological processes (sorted by Modified Fisher Exact *p*-value, EASE score by DAVID) identified by analysis of differentially regulated genes as assessed by RNA-seq of SUM52PE cells transduced with *AAMDC* shRNA #2 (sh2) compared with empty vector (EV) (Supplementary Data 2). **b** Network analysis (STRING, v11) outlining interactions between differentially regulated targets. *MTHFD1L* is outlined in red. **c** Schematic representation of 1C metabolism (folate and methionine cycles) and amino acid biosynthesis (serine and asparagine) pathways. Genes regulated by AAMDC (as per RNA-seq) are depicted in dark blue. 3PHP 3-phosphohydroxypyruvate, 3PS 3-phosphoserine, 3PG 3-phosphoglycerate, THF tetrahydrofolate, TCA tricarboxylic acid cycle. **d** Representative immunoblots (IB) showing the reduction of MTHFD1L protein expression by the *AAMDC* knockdowns (KDs) shown in Fig. 2a. Source data are provided as a Source Data file. **e** Knockdown of *MTHFD1L* by shRNA decreased cell proliferation in SUM52PE cells. Protein levels are assessed by IB (top) and cell proliferation by α-Ki-67 staining (green); Hoechst 33258-stained nuclei (blue). WT wild-type (untransduced cells), EV empty vector, *MTHFD1L* shRNAs #1–#2 (sh1–2). Representative images are shown (middle). Quantification is shown in bottom panel: WT (gray), EV (blue), shRNA #1 (orange), shRNA #2 (red). The relative quantification of Ki-67^+^ cells is normalized to EV and presented as mean values ± SD. *p*-values are determined by two-tailed unpaired *t*-test (**p = 0.0039, ****p < 0.0001). Fields of view examined: *n* = 7 for EV, *n* = 9 for WT and sh1, and *n* = 10 for sh2 over 2 independent experiments (right panel). **f** The top 25 significantly differentially regulated metabolites (WT vs. *AAMDC* sh2; *p* < 0.05, two-tailed unpaired *t*-test) identified by liquid chromatography-mass spectrometry (LC-MS) in SUM52PE cells stably transduced with either *AAMDC* sh2 or EV. *n* = 3 biologically independent experiments.

RNA-sequencing (RNA-seq) in SUM52PE cells transduced with *AAMDC* sh2 revealed that the *AAMDC* KD differentially down- and upregulated 1151 and 839 annotated genes, respectively (log_2_ fold-change, *q* < 0.05) relative to empty vector (EV) and untransduced cells (Supplementary Data 2). Gene ontology (GO) analyses of the significantly regulated targets indicated strong involvement of *AAMDC* in cell death; the cell cycle; small GTPase signaling; and various metabolic processes, including lipid, nucleotide, and amino acid biosynthesis (Fig. 3a, Supplementary Data 2). Network analysis of the differentially regulated targets revealed two functional clusters, one associated with cell proliferation (enriched in cell cycle genes) and the other with metabolism (Fig. 3b). The metabolic cluster, comprising more than 119 enzymes, was centered on a core of 26 carbon metabolism enzymes including several one-carbon (1C)/folate metabolism enzymes (all downregulated by the KD) that included *MTHFD1L* (*methylenetetrahydrofolate dehydrogenase (NADP* + *Dependent) 1-like*)) encoding a key metabolic enzyme linking mitochondrial and cytoplasmic elements of mammalian 1C metabolism (Fig. 3c). Not only did the KD of *AAMDC* downregulate MTHFD1L (Fig. 3d), but the KD of *MTHFD1L* also inhibited cell proliferation, without affecting AAMDC expression, thus suggesting that *AAMDC* acts upstream of *MTHFD1L* (Fig. 3e).

Comprehensive metabolomic profiling of *AAMDC* sh2 and controls by high-resolution liquid chromatography-mass spectrometry in SUM52PE cells revealed strong downregulation of the synthesis of nucleosides (deoxycytidine and inosine), amino acids (including proline, serine, and glycine), and metabolites associated with cellular gluco-energetics (malate and lactate; Fig. 3f and Supplementary Data 3). Interestingly, ophthalmic acid, which may be an indicator of oxidative stress associated with the metabolic depletion of glutathione^14^, was significantly upregulated by the *AAMDC* KD. The most significantly downregulated metabolite was cystathionine, the product of the enzymatic reaction catalyzed by cystathionine-β-synthase (CBS), which is an enzyme that converts homocysteine and serine to cystathionine in the methionine cycle and which is hence necessary for the synthesis of cysteine (Fig. 3c). These data confirmed that *AAMDC* regulates enzymes involved in the folate and methionine cycles of 1C metabolism such as *MTHFD1L* and *CBS*, respectively.

### AAMDC regulates PI3K-AKT-mTOR, leading to translational control of oncogenic TFs

**Fig. 4:**
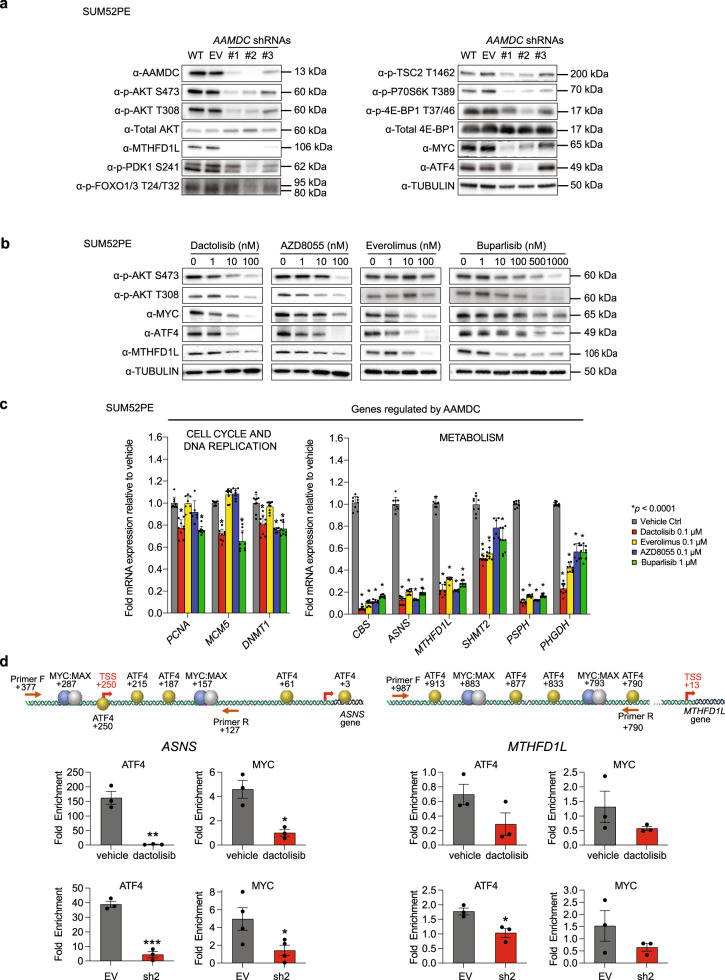
*AAMDC* knockdowns downregulate the PI3K-AKT-mTOR axis through translational suppression of MYC and ATF4 leading to transcriptional downregulation of AAMDC-dependent targets. **a** Regulation of the PI3K-AKT-mTOR pathway in SUM52PE cells transduced with *AAMDC* shRNAs assessed by immunoblotting. WT wild-type (untransduced cells), EV empty vector. Source data are provided as a Source Data file. **b** Modulation of the PI3K-AKT-mTOR pathway by pharmacological inhibition. SUM52PE cells were treated for 24 h with the indicated PI3K-AKT-mTOR inhibitors. Source data are provided as a Source Data file. **c** Transcriptional regulation of selected AAMDC-dependent transcripts involved in cell cycle and epigenetic regulation (left) and metabolic control (right). The results are normalized to vehicle-treated cells. Statistical significance is determined by multiple *t*-test using the Holm-Sidak method with alpha = 0.001 (left) and alpha = 0.05 (right), and presented as mean values ± SD, *p < 0.0001. *n* = 3 biologically independent experiments. **d**, Decreased promoter occupancy of ATF4 and MYC transcription factors at predicted promoter sites in two AAMDC targets: *ASNS* and *MTHFD1L* determined by promoter-specific chromatin immunoprecipitation (ChIP). SUM52PE cells are transduced with either empty vector (EV) or with *AAMDC* shRNA #2 (sh2), or treated with either vehicle control or 100 nM dactolisib (24 h). The position of ATF4 and MYC and the primers used for quantification are shown (top). Enrichment is determined by qRT-PCR and normalized to control cells and presented as mean values ± SEM. *p*-values are determined by two-tailed unpaired *t*-test. For *ASNS* promoter: *p = 0.0102 and *p = 0.0447 for dactolisib and sh2, respectively; **p = 0.0019, ***p = 0.0003. For *MTHFD1L* promoter: *p = 0.0201. *n* = 3 biologically independent experiments. TSS transcription start site, F forward, R reverse.

Since the PI3K-AKT-mTORC1 axis is fundamental for the control of cell proliferation and metabolism^15–18^, we next investigated whether AAMDC can modulate this signaling pathway. The *AAMDC* KDs inhibited activation of AKT relative to controls, as indicated by a reduction in p-S473 and p-T308 levels, accompanied by a decrease in MTHFD1L expression (Fig. 4a). We also observed decreased levels of p-PDK1 S241 and p-TSC2 T1462 and a reduction in the levels of p-4E-BP1 T37/46 and p-P70S6K T389, which are downstream targets of mTORC1. The total protein (but not the mRNA) levels of MYC and ATF4 were also downregulated in the *AAMDC* KDs. Since ATF4 and MYC are translationally regulated by 4E-BP1^16,19^, this suggests that *AAMDC* similarly controlled the translation of oncogenic transcription factors (TFs) at least in part via canonical PI3K-AKT-mTORC1 signaling.

We next investigated whether *TSC2* depletion could prevent the effects of *AAMDC* on PI3K-mTOR signaling. SUM52PE cells were first transduced with either EV or *TSC2* shRNA, and subsequently transduced with either EV or *AAMDC* shRNA. The *TSC2* KD partially reduced the endogenous TSC2 levels, and this was concomitant with a higher level of p-4E-BP1 relative to total 4E-BP1, thus indicating that *TSC2* KD induced mTORC1 signaling. This was also confirmed by increased MYC and p-4E-BP1 levels downstream of mTORC1. However, the *TSC2* KD did not completely prevent the effects of *AAMDC* KD on the phosphorylation of 4E-BP1 and MYC downregulation (Supplementary Fig. 3a). We also observed a reduction in the phosphorylation of another AKT-specific target, p-FOXO 1/3 (T24/32), which is not a substrate of mTORC1. Thus, AAMDC could act upstream of AKT to regulate several pathways, inclusive of but not exclusive to mTORC1 signaling (Fig. 4a).

While decreases in p-mTOR S2448 and TSC2 levels were confirmed by confocal microscopy, the intracellular localization of p-mTOR S2448 and TSC2 was not altered by the *AAMDC* KD, nor did we observe differences in the colocalization with lysosome-associated membrane protein 2 (LAMP2) (Supplementary Fig. 3b, top). Similarly, the intracellular localization of the p110α catalytic subunit of PI3K was not perturbed by the *AAMDC* KD (Supplementary Fig. 3b, bottom). However, LAMP2, and to a lesser extent the early endosomal marker EEA1, appeared to be more clustered in the *AAMDC* KD compared to the control cells. Strikingly, the *AAMDC* KD induced enlarged LAMP2^+^ late endosomal vesicles, which exhibited a degree of positive staining for p-mTOR S2448 (Supplementary Fig. 3b) and fibroblast growth factor receptor 2 (FGFR2), which is overexpressed in IntClust2 SUM52PE cells and confers activation of PI3K^20^ (Supplementary Fig. 7b). While requiring further investigation, this lysosomal phenotype also points to decreased mTORC1 signaling induced by the *AAMDC* KD.

Furthermore, pharmacological inhibition of PI3K-AKT-mTOR in SUM52PE cells by dactolisib (a dual mTORC1/2 and PI3K inhibitor), AZD8055 (an mTORC1/2 inhibitor), everolimus (an mTORC1 inhibitor), and buparlisib (a PI3K inhibitor) mimicked the effects of the *AAMDC* KDs in terms of downregulation of PI3K-AKT-mTOR. These inhibitors similarly reduced the levels of ATF4, MYC, and MTHFD1L. Furthermore, AKT activation was decreased by all of the inhibitors except everolimus (an anticipated difference as it acts downstream of mTORC2 and PDK1), thus suggesting that mTORC1 is at least sufficient to regulate MTHFD1L levels (Fig. 4b).

Pharmacological blockade of PI3K-AKT-mTOR also phenocopied the effect of the loss-of-function of AAMDC in terms of downregulation of transcription of selected cell cycle, DNA replication, and metabolic targets (Fig. 4c) discovered in our RNA-seq (Fig. 3). In particular, dactolisib was at least as effective as the other inhibitors in suppressing target gene expression and significantly more effective for particular targets (e.g., *PHGDH*; Fig. 4c). Both dactolisib and depletion of *AAMDC* reduced the binding occupancy of ATF4 and MYC to the promoters of two selected downregulated genes, *ASNS* and *MTHFD1L*, compared to control promoters lacking binding sites for these TFs (*ACTB*; Fig. 4d). These results suggest that AAMDC controls the protein levels and consequently the binding occupancy of oncogenic TFs in their targeted promoters.

**Fig. 5:**
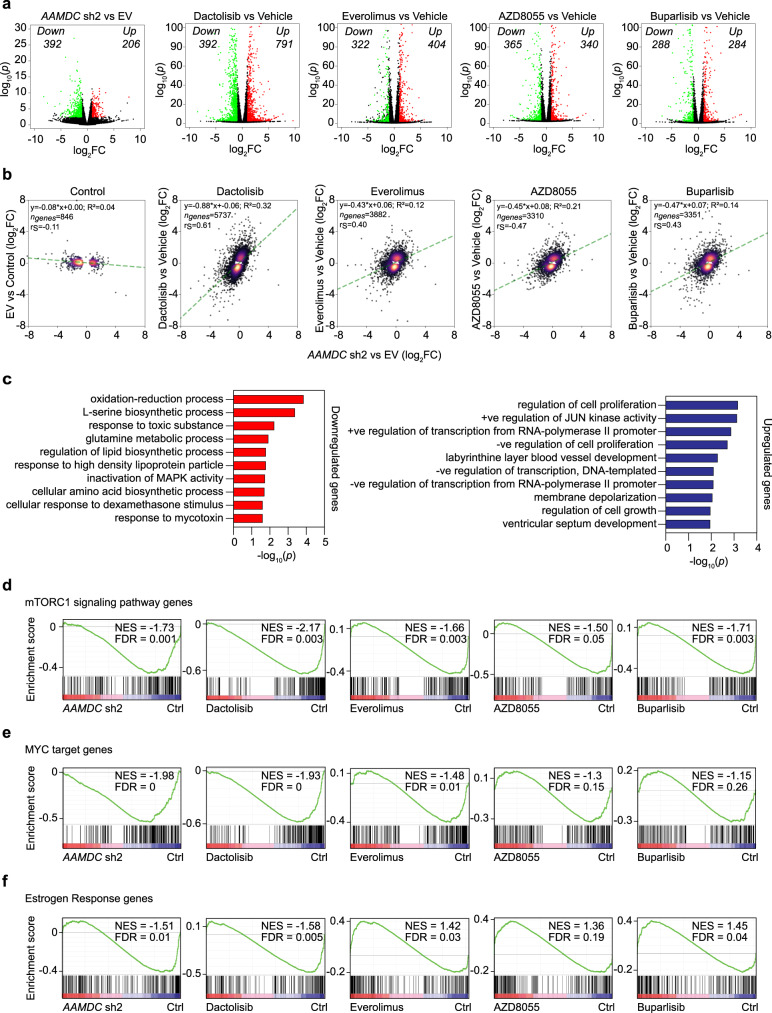
*AAMDC* knockdown and PI3K-AKT-mTOR inhibitors regulate the transcription of genes involved in common biological processes. **a** Volcano plots showing differentially expressed gene (DEG) transcripts in *AAMDC* shRNA #2 (sh2) vs. empty vector (EV) and dactolisib, everolimus, AZD8055, and buparlisib vs. dimethyl sulfoxide (DMSO) vehicle controls (left to right panels). Significantly upregulated DEGs, log_2_fold-change (log_2_FC) > 1, *P*_adj._ < 0.05, are depicted in red and significantly downregulated DEGs (log_2_FC < −1, *P*_adj._ < 0.05) in green. **b** Scatter plots outlining the distribution of all significant DEGs (*P*_adj._ < 0.01) in EV vs. WT (wild-type) control (*y*-axis) against *AAMDC* sh2 vs. EV (*x*-axis) and dactolisib, everolimus, AZD8055, and buparlisib vs. DMSO controls (*y*-axis) against *AAMDC* sh2 vs. EV (*x*-axis) (left to right panels). A linear model is shown (green line) (R^2^ indicating model fit) and the Spearman correlation (r_S_) between the outlined conditions. Color represents gene density within that region of the plot: low density (black) to high density (yellow). **c** Gene ontology (GO) analysis of 186 downregulated and 123 upregulated genes (*P*_adj_. <  0.01) that are common in the *AAMDC* sh2 sample for at least three of the drug treatments. The top 10 biological processes are ranked by the *p*-values. **d** Gene set enrichment analysis (GSEA) plot of mTORC1 signaling targets shown in Supplementary Data 4 in *AAMDC* sh2 compared with EV, as well as dactolisib, everolimus, AZD8055, and buparlisib compared with DMSO control (Ctrl) (left to right panels). The *y*-axis and the green line show the enrichment score for each gene, illustrated as a vertical line plotted in rank order of the most gene abundance (red, left) to the least gene abundance (blue, right) within the indicated samples (as log_2_FC/comparison); the black vertical lines correspond to member genes from the set. NES normalized enrichment score, FDR false discovery rate. **e** As in (**d**) for the MYC target genes shown in Supplementary Data 4. **f** As in (**d**) for the estrogen-responsive genes shown in Supplementary Data 4.

The genomic selectivity of *AAMDC* relative to that of the PI3K-mTOR inhibitors was examined by comparative transcriptomics in SUM52PE cells (Fig. 5a). The RNA-seq from the *AAMDC* KD was analyzed in parallel with drug-treated samples (dactolisib, everolimus, AZD8055, or buparlisib) to enable cross-examination of differentially expressed genes (DEGs). We found a stronger trend for DEGs in dactolisib-treated cells to up- or downregulate in the same direction as the DEGs in the *AAMDC* KD, thus suggesting similar downstream targets, compared to the other inhibitors that were tested (Fig. 5b).Various metabolic processes were enriched in the gene ontology (GO) term analysis for genes commonly downregulated between the KD and at least three of the drug treatments (Fig. 5c, Supplementary Fig. 4, Supplementary Data 4). Upregulated GO terms included regulation of cell proliferation and transcription (Fig. 5c). Gene set enrichment analysis also revealed depletion of mTORC1 signaling and MYC target genes in the *AAMDC* KD and all of the drug-treated samples (Fig. 5d–e, Supplementary Data 4). Interestingly, although ER response genes were depleted in the *AAMDC* KD and dactolisib-treated cells, we observed an opposite trend, namely upregulation of ER and AR response genes, in cells treated with everolimus, AZD8055, and buparlisib (Fig. 5f). Among the AAMDC-specific genes (Supplementary Fig. 5, Supplementary Data 4), we identified unique targets involved in PI3K-dependent vesicle trafficking and lysosomal degradation of cellular receptors, thereby substantiating a role of AAMDC in the regulation of endosomal trafficking.

### *AAMDC* overexpression activates AKT signaling and induces estrogen-independent tumor growth in vivo

**Fig. 6:**
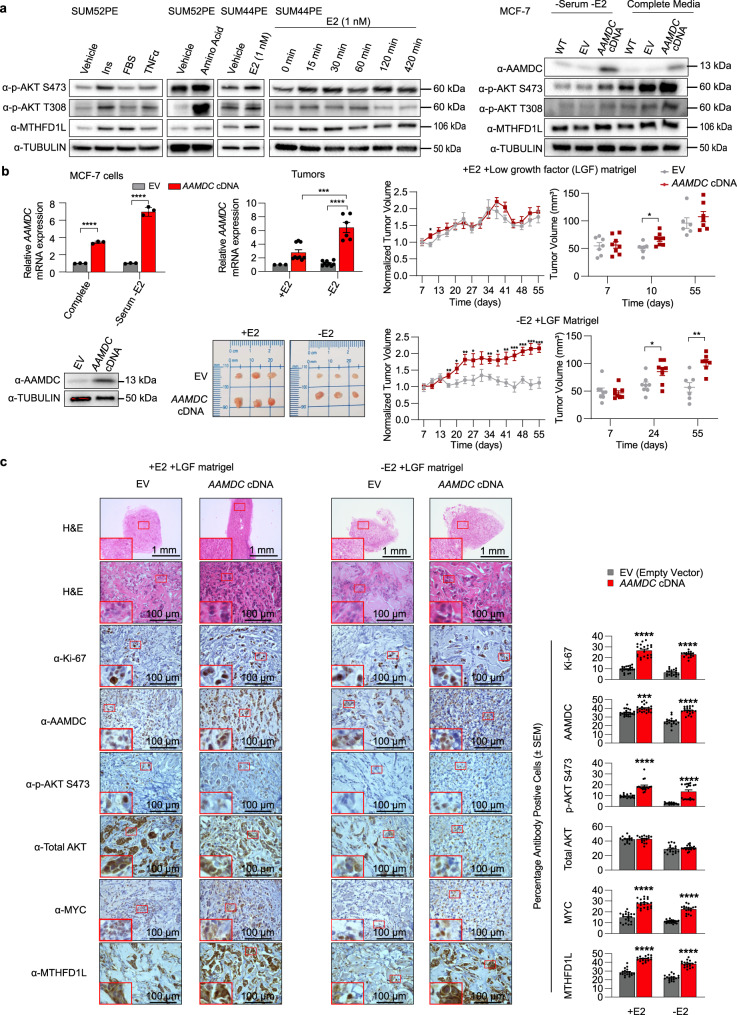
AAMDC activates PI3K-AKT-mTOR signaling and promotes E2-independent tumor growth in vivo. **a** Activation of PI3K-AKT-mTOR signaling by various ligands increases MTHFD1L expression, as determined by immunoblot (IB). Panels 1 and 2 (from left to right): Serum-starved SUM52PE subjected to growth factor stimulation for 24 h with insulin (Ins, 300 μg/mL), fetal bovine serum (FBS, 20%), tumor necrosis factor α (TNFα, 50 ng/mL), and amino acids (4 h). Panel 3: The estrogen receptor positive (ER^+^) cell line SUM44PE was estrogen-starved for 72 h and then stimulated with estrogen (E2, 1 nM) for 24 h. Panel 4: Time-course of E2-mediated activation of the PI3K-AKT-mTOR-MTHFD1L axis in SUM44PE cells. Panel 5: Activation of PI3K-AKT-mTOR upon lentiviral transduction of *AAMDC* cDNA in MCF-7 cells compared to empty vector (EV) and untransduced wild-type (WT) cells grown either under starvation conditions (in the absence of serum, estrogen, or non-essential amino acids) or in complete medium for 24 h prior to immunoblotting with the indicated antibodies (α). Source data are provided as a Source Data file. **b** Left: mRNA (top) and protein expression (bottom) levels in MCF-7 cells lentivirally transduced either with *AAMDC* cDNA or EV grown under starvation conditions prior to their injection into nude mice. mRNA levels for the same cells grown in complete medium are shown. Middle: mRNA expression of *AAMDC* in the extracted tumors (top) with representative images of the tumors indicated (bottom), harvested at day 55. Right: Mean tumor volumes in nude mice injected with low growth factor (LGF) Matrigel® and with MCF-7 cells transduced with either the EV or *AAMDC* cDNA. Mice were injected with 1 μg of estradiol valerate (+E2, top) or with vehicle control in the absence of estrogen (-E2, bottom). The mean tumor volume of *n* = 8 mice is normalized to day 7 (left). Volume measurements of individual mice are plotted for the selected time-points shown (right). Statistical significance is determined using a two-tailed unpaired *t*-test and presented as mean values ±  SEM. For MCF-7 cells and tumors: ***p = 0.0005, ****p < 0.0001. Multiple unpaired *t*-test for tumor volume +E2 + LGF: *p = 0.0214, left; *p = 0.0111, right. For tumor volume −E2 + LGF (left plot): day 16 **p = 0.0086, day 20 *p = 0.0277, day 24 **p = 0.0052, day 27 *p = 0.0339, day 34 **p = 0.0026, day 38 *p = 0.0132, day 41 **p = 0.0042, day 45 ***p = 0.0009, day 48 ***p = 0.0002, day 52 ***p = 0.0004, day 55 ***p = 0.0003; (right plot): day 24 *p = 0.0175, day 55 **p = 0.0011). Source data are provided as a Source Data file. **c** Immunohistochemistry (IHC) analysis of two representative tumors collected at day 55 for each condition. Tumor sections were stained with hematoxylin and eosin (H&E) or the antibodies indicated and the images quantified. The error bars indicate the mean ± SEM. Statistical significance between empty vector (EV) and *AAMDC* cDNA conditions is determined by a two-tailed unpaired *t*-test (***p = 0.0002, and ****p < 0.0001). *n* = 3 biologically independent animals.

Consistent with the model of action of *AAMDC*, insulin, fetal bovine serum (FBS), amino acids, and tumor necrosis factor α (TNF-α) activated AKT signaling, thereby increasing MTHFD1L expression (Fig. 6a). Treatment of insulin-dependent SUM52PE cells with insulin partially rescued the effects of *AAMDC* KD, as assessed by detection of p-AKT S473 and MTHFD1L by immunoblotting (Supplementary Fig. 3c). Similarly, estrogen (β-estradiol, E2) led to fast and strong activation of this pathway in ER^+^ SUM44PE IntClust2 cells, thus suggesting a non-genomic effect, with a biphasic pattern resulting in peaks of activation after 30 and 420 min of exposure to the ligand (Fig. 6a). Of note, the endogenous *AAMDC* levels were not modulated by treatment of ER^+^ cells with E2, thus suggesting that *AAMDC* is not, in itself, an estrogen-responsive gene (Supplementary Fig. 3d). Interestingly, the exogenous levels of both *AAMDC* mRNA and protein were greater in transgenic *AAMDC*-MCF-7 cells subjected to starvation conditions compared to cells grown in complete medium (Fig. 6a, right and 6b, left), thus suggesting the occurrence of post-transcriptional feedback regulatory loops^13^. Overexpression of the *AAMDC* cDNA in MCF-7 cells also resulted in constitutive AKT activation, particularly in cells depleted of FBS, insulin, and E2, and thus the *AAMDC* cDNA activated the pathway in conditions of nutrient deprivation or metabolic stress (Fig. 6a, right).

To assess the oncogenic function of *AAMDC* in vivo, we injected MCF-7 cells overexpressing either *AAMDC* cDNA or EV into immunodeficient mice (Fig. 6b). The tumor cells were injected subcutaneously with low growth factor (LGF)-containing Matrigel® and supplemented either with injections of 1 μg of E2 (20 μg/mL) every 3–4 days (+E2) or in the absence of E2 supplementation (−E2). As expected, the EV tumors grew in the +E2 condition but they failed to proliferate in the −E2 condition. In the +E2 conditions, the *AAMDC*-overexpressing tumors only had significantly higher tumor volumes relative to the controls at day 10 post-injection (Fig. 6b, top right). However, in the −E2 condition, *AAMDC* significantly activated tumor growth at all of the time points investigated and reached volumes at day 55 post-injection comparable to those of tumors grown in the +E2 condition (Fig. 6b, bottom right). The MCF-7-*AAMDC* tumors sustained *AAMDC* overexpression even at day 55 post-injection, with those in the E2-deprived conditions exhibiting significantly greater levels of *AAMDC* mRNA than the +E2 tumors (Fig. 6b, middle).

Representative tumors resected at day 55 post-injection confirmed the overexpression of AAMDC and its downstream selected effectors p-AKT S473, MYC, and MTHFD1L. The *AAMDC* cDNA sections exhibited more dense islands of tumor cells and higher levels of Ki-67 (notably in the −E2 condition) relative to the EV (Fig. 6c). Interestingly, the MCF-7-*AAMDC* tumors in the −E2 condition exhibited a higher frequency of nuclear MYC and p-AKT S473 staining than the +E2 tumors. These results validated the oncogenic role of *AAMDC* in activating PI3K-AKT-mTOR in vivo, particularly in conditions of growth factor and E2 deprivation.

### Pharmacological inhibition of AAMDC-dependent targets by PI3K-mTORC1 blockers in combination with anti-estrogens

**Fig. 7:**
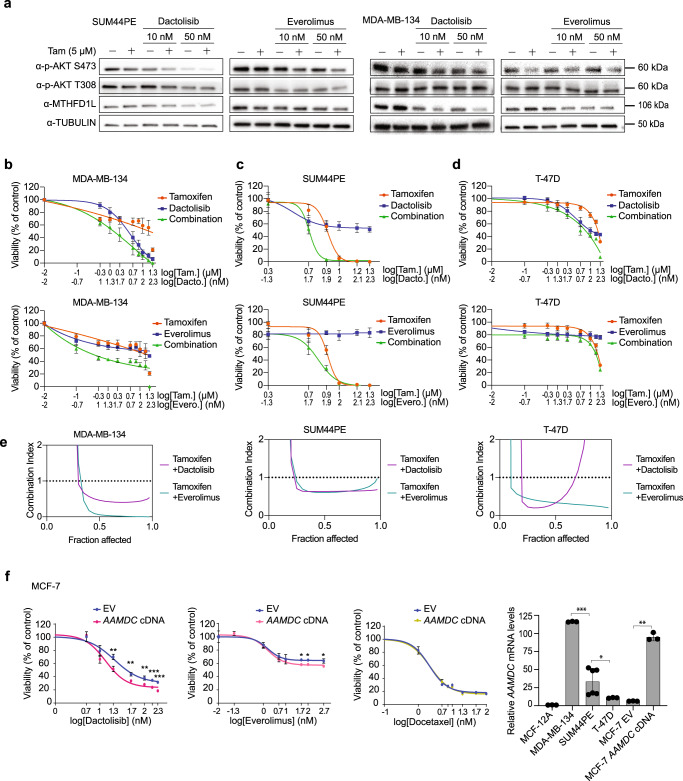
Sensitization of luminal IntClust2 cell lines expressing AAMDC by co-treatment with PI3K-AKT-mTORC1 blockers in combination with tamoxifen. **a** Pharmacological blockade of PI3K-AKT-mTOR leads to downregulation of MTHFD1L expression in estrogen receptor positive (ER^+^) intercluster 2 (IntClust2) breast cancer cell lines (SUM44PE and MDA-MB-134) treated with tamoxifen (Tam, 5 μM) in combination with dactolisib or everolimus (24 h, at the concentrations indicated in the blots). Source data are provided as a Source Data file. **b**–**d** Dose-dependent changes in cell viability in the ER^+^ breast cancer cell lines MDA-MB-134 (**b**), SUM44PE (**c**), and T-47D (**d**) treated with dactolisib (Dacto., top, blue) or everolimus (Evero., bottom, blue), with tamoxifen (Tam., red) and as combinations (green). Data are presented as mean values ± SD. *n* = 3 biologically independent experiments. Viability is determined using a luminescence assay (CellTiter-Glo®) after 72 h post-treatment. **e** Tamoxifen and selected inhibitors of the PI3K-AKT-mTOR pathway exhibit synergistic pharmacological interactions in inhibiting tumor cell viability. Plots indicating the combination index (CI) for tamoxifen with dactolisib (purple) and tamoxifen with everolimus (cyan) in MDA-MB-134 (left), SUM44PE (middle), and T-47D (right) cells. CI < 1 synergistic, CI = 1 additive, and CI > 1 antagonistic. The CI is calculated from the average of three independent cell viability assays by the CI index method. Fraction affected is the fraction of non-viable cells. **f** Dose-dependent changes in cell viability in the MCF-7 cell line stably overexpressing *AAMDC* cDNA compared to empty vector (EV) treated with dactolisib, everolimus, and docetaxel. Statistical significance is determined for biological triplicates and presented as mean values ± SD and *p*-values are determined by multiple unpaired *t*-tests. For dactolisib, from left to right: **p = 0.0007, **p = 0.0013, **p = 0.0016, ***p < 0.0001, ***p < 0.0001; for everolimus, from left to right: *p = 0.0062, *p = 0.0053, *p = 0.0066. Bottom right: Relative *AAMDC* mRNA expression in a panel of ER^+^ breast cancer cell lines normalized to the non-tumorigenic epithelial line MCF-12A, as assessed by qRT-PCR. Data presented as mean values ± SD and *p*-values are determined by two-tailed unpaired *t*-test with Welch’s correction (*p = 0.0308, **p = 0.0012, ***p = 0.0001).

We next investigated whether the effects of AAMDC could be mitigated through combination treatment with PI3K-mTORC1 inhibitors and the ER modulator tamoxifen. Treatment of ER^+^ SUM44PE and MDA-MB-134 cells with tamoxifen in combination with everolimus or dactolisib resulted in greater inhibition of AKT signaling and MTHFD1L expression than with either drug alone (Fig. 7a).

In addition, the dual PI3K-mTOR inhibitor dactolisib more potently inhibited cell viability than the mTORC1-specific inhibitor everolimus, particularly in the ER^+^ IntClust2 cell line MDA-MB-134, which showed the highest level of endogenous *AAMDC* (Figs. 1f, 7f, right) and exhibited resistance to tamoxifen alone across all concentrations tested (Fig. 7b–d). Moreover, we observed highly synergistic pharmacological interactions between these PI3K-mTOR inhibitors with tamoxifen in IntClust2 cell lines, with combination indexes (CIs) < 1 (Fig. 7e). Furthermore, overexpression of *AAMDC* in MCF-7 cells also increased the sensitivity to dactolisib and, to a lesser extent, everolimus, but not to the non-selective cytotoxic drug docetaxel (Fig. 7f).

### AAMDC binds to the GTPase-activating protein RabGAP1L

To determine the direct binding partners of AAMDC, we conducted two independent yeast two-hybrid (Y2H) screens using bait plasmids *N-LexA-AAMDC-C* and *N-Gal4-AAMDC-C* and a cDNA library from luminal BC cells. We identified RabGAP1L (Rab GTPase-activating Protein 1-like) as a high-confidence prey, being the only target identified in both independent screens (Supplementary Data 5). RabGAP1L is a signal transduction protein that binds and regulates Rab GTPases, thereby controlling protein trafficking^21^.

**Fig. 8:**
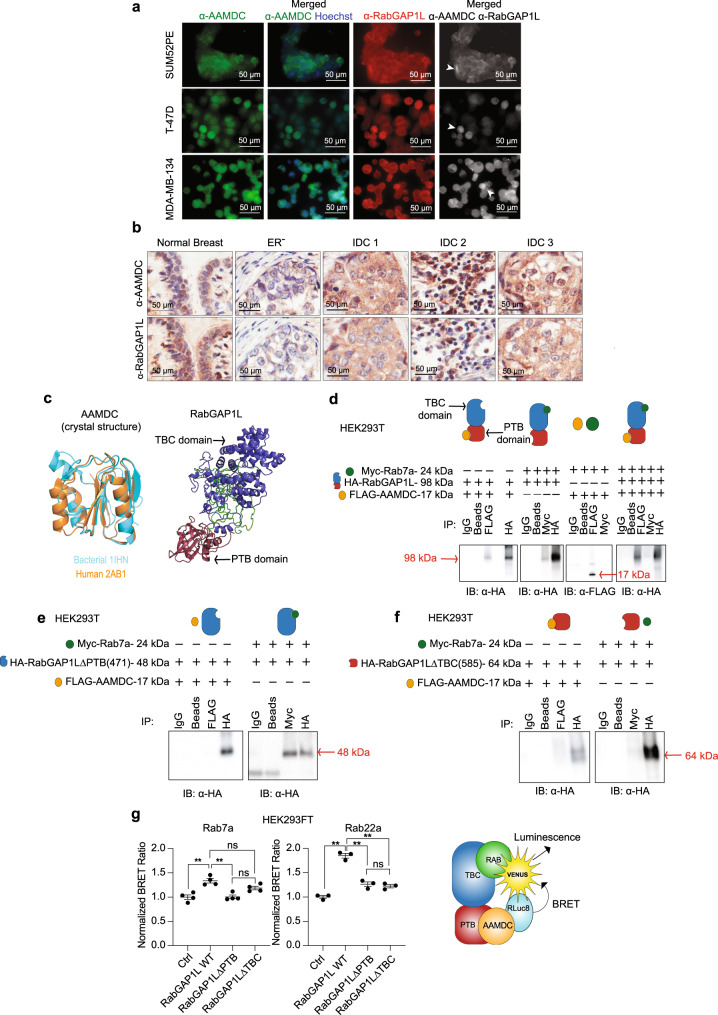
AAMDC interacts with the GTPase activating protein RabGAP1L. **a** Colocalization of endogenous AAMDC and RabGAP1L proteins in luminal breast cancer cells assessed by immunofluorescence (IF). Hoechst 33258-stained nuclei (blue), α-AAMDC antibody (green), and α-RabGAP1L antibody (red). Arrows indicate regions of strong signal overlap. **b** Localization of AAMDC and RabGAP1L in adjacent representative sections of normal breast, and in selected estrogen receptor negative (ER^−^) or invasive ductal carcinoma (IDC) breast cancer specimens assessed by immunohistochemistry (IHC). **c** Superposition of the crystal structure of bacterial (1IHN, cyan, 10.2210/pdb1IHN/pdb)^11,54^ and human (2AB1, orange, 10.2210/pdb2AB1/pdb)^55,74^ AAMDC proteins (left) and the Phyre2 (v2.0) homology model of human RabGAP1L. The phosphotyrosine-binding (PTB) domain is shown in red and the C-terminal Tre-2/Bub2/CdC16 (TBC) Rab-binding domain in violet (right). **d**–**f** Interaction between AAMDC, RabGAP1L, and Rab7a by immunoprecipitation (IP) in HEK293T cells transiently transfected with full-length **(d)** or deletion mutants **(e**–**f**) of the tagged cDNAs: *HA-RabGAP1L*, *FLAG-AAMDC*, and *Myc-Rab7a*. The IPs were immunoblotted with an α-HA antibody to detect HA-RabGAP1L (98 kDa) or α-FLAG to detect FLAG-AAMDC (17 kDa). Deletion of the PTB domain is indicated by HA-RabGAP1LΔPTB(471) (48 kDa) (**e**), and deletion of the Rab-binding TBC domain by HA-RabGAP1LΔTBC(585) (64 kDa) (**f**); α-IgG-conjugated beads and beads only are used as a control. **g** Bioluminescence resonance energy transfer (BRET) assays in HEK293FT cells transiently overexpressing *Venus-HA-Rab7a* or *Venus-Rab22a*, *RLuc8-AAMDC*, full-length *RabGAP1L*, *RabGAP1LΔPTB*, or *RabGAP1LΔTBC*. Control cells (Ctrl) are transfected with *Venus-Rab* and *RLuc8-AAMDC* only. The BRET ratio values are normalized to the respective controls. The individual values for *n* = 4 biological replicates (Rab7a) and for *n* = 3 biological replicates (Rab22a) are shown as mean values ± SEM, and statistical significance is determined using Brown-Forsythe and Welch ANOVA tests with Dunnett’s T3 multiple comparison test (Rab7a: **p = 0.0096 for Ctrl vs. RabGAP1L WT, **p = 0.0069 for RabGAP1L WT vs. RabGAP1LΔPTB, and not significant (ns) for RabGAP1L WT vs. RabGAP1LΔTBC and for RabGAP1LΔPTB vs. RabGAP1LΔTBC; Rab22a: **p = 0.0024 for Ctrl vs. RabGAP1L WT, **p = 0.0045 for RabGAP1L WT vs. RabGAP1LΔPTB, **p = 0.0020 for RabGAP1L WT vs. RabGAP1LΔTBC, and ns for RabGAP1LΔPTB vs. RabGAP1LΔTBC).

Both RabGAP1L and AAMDC were co-expressed on the plasma-membrane, in the cytoplasm, and/or in the nucleus of luminal cancer cells, as assessed by IF (Fig. 8a). A similar pattern of costaining, both in terms of intensity and intracellular localization, was observed in serial full-face sections taken from clinical breast tissue specimens, as assessed by IHC (Fig. 8b).

The Y2H experiments revealed that the minimal interaction domain of AAMDC comprised the phosphotyrosine binding (PTB) domain of RabGAP1L (Supplementary Data 5) and, consistent with this, AAMDC contains a PTB binding consensus motif (NTTY). In contrast, RabGAP1L binds small Rab GTPases via its highly conserved Tre-2/Bub2/cdc16 (TBC) domain (Fig. 8c).

We next investigated the interactions between AAMDC and RabGAP1L and a subset of selected Rabs: Rab13, Rab22a, Rab7a, and Rab35. Rab13 and Rab22a were chosen as they are known interactors of RabGAP1L^21,22^, while Rab7a was captured as a binding partner of AAMDC^22^. Similar to AAMDC, oncogenic Rab35 constitutively activated p-AKT S473 in a ligand-independent manner^23^.

Immunoprecipitations (IPs) with tagged constructs in HEK293T cells confirmed that HA-RabGAP1L can bind FLAG-AAMDC, and that HA-RabGAP1L can be found associated with selected Myc-Rab proteins (particularly Rab7a, as shown in Fig. 8d, as well as Rab13 and Rab35, see Supplementary Fig. 6a). Reciprocal IPs with α-HA-RabGAP1L beads failed to capture AAMDC and/or Rab proteins, which could be due to steric inaccessibility of their binding epitopes in the RabGAP1L pull-downs (Supplementary Fig. 6c and d). In the absence of RabGAP1L expression, no direct interaction between the Rabs and AAMDC was observed (Fig. 8d, Supplementary Fig. 6a). Moreover, when the three plasmids (FLAG-*AAMDC/HA-RabGAP1L/Myc-Rab*) were co-transfected, pull-downs with α-FLAG-AAMDC confirmed the association between AAMDC and RabGAP1L. Pull-downs by α-Myc-Rab-coated beads resulted in a much weaker capture of HA-RabGAP1L (Fig. 8d; Supplementary Fig. 6a). Thus, in these assays, *AAMDC* overexpression could influence the binding and/or dissociation of certain Rab proteins from RabGAP1L, but this will require further experiments for validation.

Subsequent IPs showed that RabGAP1L and AAMDC bound Ankyrin-B (AnkB), which has been shown to recruit RabGAP1L in PI3P^+^ vesicles^21^ (Supplementary Fig. 6e). Regions of AAMDC-AnkB overlap were predominant near the plasma membrane and in the cytoplasm, as assessed by confocal microscopy (Supplementary Fig. 6f). Live-cell microscopy confirmed several regions of overlap between 2xFYVE N-terminally fused to mCherry, a marker of phosphatidylinositol 3-phosphate (PI3P) with eGFP-RabGAP1L, thereby providing evidence that RabGAP1L may colocalize, at least in part, in PI3P^+^ vesicles, some of which were Rab7a^+^ late endosomes (Supplementary Fig. 7a).

Deletion of the PTB domain of RabGAP1L [*HA-RabGAP1L*Δ*PTB(471)*] suppressed the interaction between AAMDC and RabGAP1L, as assessed by IPs in HEK293T cells, but retained the RabGAP1L-Rab interactions (Fig. 8e, Supplementary Fig. 6b). In contrast, deletion of the TBC domain [*HA-RabGAP1L*Δ*TBC(585)*] suppressed both the interaction between RabGAP1L and AAMDC as well as the binding of Rab proteins (Fig. 8f, Supplementary Fig. 6b).

To analyze the physical proximity of AAMDC, RabGAP1L, and Rab proteins in intact cells we conducted Bioluminescence Resonance Energy Transfer (BRET) experiments. HEK293FT cells were co-transfected with luciferase-tagged *AAMDC* (*RLuc8-AAMDC*), fluorophore-tagged *Rabs* (*Venus-tagged Rabs*), and either untagged full-length *RabGAP1L*, *RabGAP1L*Δ*PTB*, or *RabGAP1L*Δ*TBC*. Co-transfection of *AAMDC*, *RabGAP1L*, and selected *Rabs* resulted in a consistently greater BRET signal compared to that of *AAMDC* in the absence of RabGAP1L (control). Transfection of *RabGAP1L*Δ*PTB* or *RabGAP1L*Δ*TBC* produced a decay in BRET signal relative to the full-length *RabGAP1L*, particularly with *Rab7a, Rab22a*, and *Rab35* (Fig. 8g and Supplementary Fig. 8a).

Further IF assays with transfected HEK293T cells provided evidence that the RabGAP1L staining could overlap with that of Rab7a (Supplementary Fig. 8b), which is a finding consistent with our live-cell imaging (Supplementary Fig. 7a). In contrast, RabGAP1LΔPTB and RabGAP1LΔTBC exhibited a more diffuse cytoplasmic pattern of expression, with negligible areas of overlap with Rab7a, in accordance with the BRET data (Supplementary Fig. 8b).

Lastly, we investigated the dependency of the AAMDC-RabGAP1L interaction in activating AKT signaling. We found that *RabGAP1L* KD in SUM52PE cells had similar effects as the *AAMDC* KD, reducing p-AKT S473 and MTHFD1L levels (Supplementary Fig. 9a). Furthermore, lentiviral overexpression of *RabGAP1LΔPTB* and *AAMDC-DAAE*, which has the PTB binding consensus sequence 20-NTTY-23 mutated to DAAE reduced AKT signaling (Supplementary Fig. 9b). The RabGAP1LΔPTB mutant phenotype, however, was mild and reversible and it progressively reverted towards a phenotype sensitive to the effects of the *AAMDC* KD (Supplementary Fig. 9c), which could be due to competition between the RabGAP1L mutant transgene with the endogenous gene. In addition, both *RabGAP1L*Δ*PTB* and *AAMDC-DAAE*, but not WT *RabGAP1L* and *AAMDC* cDNAs, phenocopied the effects of the *AAMDC* KDs in inducing F-actin remodeling (Supplementary Fig. 9d). These experiments indicated that functional effects of *AAMDC* KDs could be phenocopied by suppression of RabGAP1L expression or by alteration of its predicted binding interfaces with AAMDC.

In summary, our results indicate that AAMDC constitutively activates PI3K-AKT-mTORC1 signaling to support tumor growth, notably under conditions of metabolic stress such as growth factor and E2 deprivation. We propose that AAMDC regulates the translation of oncogenic TFs such as MYC and ATF4, which in turn could control the transcription of several target genes involved in cell cycle control and metabolism (Supplementary Fig. 10). We also describe AAMDC-RabGAP1L physical interactions that could mediate some of the functional facets of AAMDC for induction of AKT signaling, thereby highlighting potentially druggable interfaces for future therapeutic intervention.

## Discussion

Herein, we have unveiled the function of *AAMDC*, an oncogene selectively amplified in ~25% of luminal B BCs, which often exhibit anti-E2 resistance and very poor prognoses^24,25^. We propose that AAMDC orchestrates the coordinate control of proliferation and metabolism by regulating the PI3K-AKT-mTOR signaling axis. The *AAMDC* KDs inhibited the translation and thereby the promoter occupancy of MYC and ATF4 at the *AAMDC*-responsive promoters. Notably, we show that AAMDC is involved in the regulation of the expression of enzymes participating in the folate and methionine cycles of 1C metabolism (such as *MTHFD1L*, *ASNS*, *SHMT2*, and *CBS)* and in lipid metabolism. Supporting our model, there is abundant evidence that mTORC1 regulates the cap-dependent translation of MYC^15,19,26–28^ and ATF4^16,29^, and that mTORC1 modulates metabolic genes transcription^16,29–31^.

Our findings illuminate metabolic vulnerabilities that could be therapeutically exploited, more specifically, to target the malignancies carrying the *AAMDC* amplification. Excitingly, the metabolite most downregulated by *AAMDC* KD was cystathionine, which is a product of the CBS enzyme involved in the methionine cycle and implicated in tumor survival in breast cancer^32^ and lipid metabolism in ovarian cancer^33^. In addition, the effects of *AAMDC* KD in normal breast epithelial cells were mild, thus suggesting that IntClust2 cells could be more addicted to *AAMDC* expression.

Our comparative transcriptomics data suggests that *AAMDC* KD is more similar to the dual PI3K-mTOR blocker dactolisib than to single PI3K and mTOR blockers. Moreover, our rescue experiments highlighted that the AAMDC effects are mediated by but not exclusively to mTORC1. This finding was reinforced by our RNA-seq data that also pointed to unique AAMDC targets involved in catabolic metabolism of cellular receptors, lysosome/vacuole organization, and endosomal trafficking, thereby further highlighting more specific biological features induced by AAMDC.

In vivo, *AAMDC* overexpression was sufficient to activate the growth of E2-dependent tumor cells under metabolic stress conditions (low levels of growth factor and E2 deprivation). This was accompanied by activation of AKT signaling in vivo, with greater nuclear localization of p-AKT S473 and MYC relative to control tumors. It is known that constitutive activation of AKT leads to E2-independent tumor growth in vivo^34–36^, and that breast cancer cells adapt to long-term E2 deprivation with increases in PI3K-AKT activity, thereby becoming dependent on PI3K signaling for growth^37^. Our findings suggest a role of *AAMDC* as a molecular metabolic switch whereby upregulation of *AAMDC* through copy number amplification could help sustain ligand-independent activation of PI3K-AKT, and thus supporting tumor survival under metabolic stress conditions, such as E2 deprivation. This mechanism could potentially be of great significance in patients undergoing anti-E2 treatments.

Importantly, we suggest that some oncogenic facets of AAMDC for activation of AKT may be dependent on the interaction between AAMDC and the PTB domain of RabGAP1L. Further, we present evidence that AAMDC, RabGAP1L, and Rab7a could be localized in endolysosomes, which would provide them with an opportunity to interact. Likewise, there is potential for AAMDC and RabGAP1L to associate with additional Rabs, as suggested by our biochemical and BRET data, but this remains to be verified based on colocalization and other approaches.

Future investigation will be required to elucidate the exact molecular mechanism of action by which AAMDC activates the PI3K-AKT-mTOR pathway. Small GTPases are implicated in multiple aspects of PI3K and mTOR regulation^23,38–44^. In this context, the AAMDC-RabGAP1L complex could play a role in the regulation of vesicle trafficking, potentially altering the recycling of small G proteins and thus remodeling the cancer signaling network. These effects could also explain the multiple differential localizations of AAMDC and RabGAP1L in human tumors. It is conceivable that AAMDC could modulate AKT signaling by alteration of the association or release of particular Rab proteins from RabGAP1L, and potentially modulating their intrinsic GTPase activity. The enlarged LAMP2^+^ vesicles induced by the AAMDC KDs that stained positive for p-mTOR S2448 and FGFR2 supports a mechanism of aberrant mTOR signaling through impaired lysosome function via degradation of cellular receptors, such as FGFR2.

Ras-like Rab oncoproteins^45^ are traditionally considered difficult-to-drug and thus our work opens up new avenues for the development of informed treatments for these aggressive BCs^24,25^. We found that combination treatments of dactolisib or everolimus with tamoxifen also decreased cell viability in a highly synergistic manner in IntClust2 ER^+^ cell lines expressing high levels of *AAMDC*. The activity of everolimus in combination with anti-E2 therapy in clinical trials is such that it is now used routinely with anti-estrogens in the setting of metastatic BC resistant to anti-E2 treatment alone^46^. However, the failure of translational studies to find an effective biomarker of activity^47^, in combination with the relatively high toxicity associated with the drug, has precluded both wider use in the metastatic setting as well as progression of this combination to adjuvant trials. In regard to dactolisib, there is pre-clinical evidence of synergy with anti-estrogens^48^, although again the toxicity profile is such that a biomarker of efficacy is likely to be required to obtain a viable therapeutic profile for routine usage^49^. In this context, our work indicates that AAMDC could just be such a biomarker and that targeting AAMDC signaling with PI3K-AKT-mTOR inhibitors and potentially more specifically, targeting key binding interfaces with effectors (such as the PTB domain of RabGAP1L), may be an effective and more selective treatment for IntClust2 cancers or other malignancies that have *AAMDC* amplification.

## Methods

Additional information of key resources used for undertaking this study is provided in Supplementary Data 6.

### Human studies

The immunohistochemistry (IHC) of estrogen receptors, as well as the *AAMDC* gene amplification studies by FISH, were performed on a total of 119 samples. All of the patients were women between 31 and 91 years of age. Luminal B tumors were defined either by ER and HER2 co-expression or by a high tumor grade in ER-expressing malignancies. The study was approved by the medical review board of the Medical University of Gdansk (NKBBN/205/2017). Informed consent for the acquisition and the use of patient’s samples was obtained.

### Animal studies

The animal experiments were performed in accordance with the protocols approved by the Animal Ethics Committee of the University of Western Australia (RA/3/100/1159 and RA/3/100/1687). Five-week-old female BALB/cJ Foxn1nu/Arc (Nude mice) were obtained from the Animal Resources Centre (Canning Vale, Australia) and used for the breast cancer xenograft growth study. All animals received adequate environment enrichment, which includes housing with other animals where possible. Mice were housed in Techniplast GM500 cages (30 cm  × 16.7 cm × 13.5 cm) (*n* = 4/cage) on a coarse aspen bedding with a paper towel, a tissue, a cotton nestlet, and an aspen gnawing block for enrichment. Room temperature and humidity was maintained at 22.5 °C and between 30% and 70%, respectively. All mice were held under 12:12 (12-h light:12-h dark) with light increasing gradually in both the morning and evening. The mice were randomly assigned to the different experimental groups.

### Oncogenomic datasets and portals

Analysis of somatic alterations for the *AAMDC* gene (Fig. 1a) was performed by examination of cancer genomic datasets and tools available from cBioPortal^50,51^ (https://www.cbioportal.org/results/cancerTypesSummary?cancer_study_list=brca_metabric%2Cbrca_igr_2015%2Cbrca_mbcproject_wagle_2017%2Cbrca_tcga_pub2015%2Cov_tcga_pan_can_atlas_2018%2Cnsclc_tcga_broad_2016%2Cluad_tcga_pan_can_atlas_2018%2Chnsc_tcga_pub%2Cblca_tcga_pub_2017%2Cprad_mskcc_2017%2Cnepc_wcm_2016%2Cskcm_tcga_pub_2015&Z_SCORE_THRESHOLD=2.0&RPPA_SCORE_THRESHOLD=2.0&data_priority=0&profileFilter=0&case_set_id=all&gene_list=AAMDC&geneset_list=%20&tab_index=tab_visualize&Action=Submit). The survival analyses of the cancer patients with high and low expression levels of *AAMDC* (Fig. 1b) were performed with the PPISURV portal^50,52^ (http://www.bioprofiling.de/GEO/PPISURV/ppisurv.html). The survival analyses of breast, ovarian, and lung cancer patients were performed by investigating the GSE11121, GSE13876, and GSE19188 GEO datasets, respectively. Survival of Luminal B patients treated with tamoxifen with high and low expression of AAMDC was compared using Kaplan–Meier plotter server^53^ and the GEO datasets: GSE12093, GSE16391, GSE17705, GSE19615, GSE26971, GSE2990, GSE3494, GSE37946, GSE45255, GSE6532, and GSE9195.

### Molecular modeling

Molecular modeling of the bacterial and human AAMDC protein structures was performed with Phyre2 (v2.0). Protein Data Bank (PDB) structures shown in Fig. 8 are available under the accession codes IHN 10.2210/pdb1IHN/pdb^54^ and 2AB1, 10.2210/pdb2AB1/pdb^55^.

### Cell culture

All of the cell lines used in the study were authenticated, *Mycoplasma*-free and commercially available. All of the cells were cultured at 37 °C in a 5% CO_2_ incubator in a humidified atmosphere. The MDA-MB-231 and HEK293T cells were grown in DMEM high glucose-pyruvate medium containing 10% FBS and 1% antibiotic-antimycotic. The HEK293FT cells were cultured in DMEM containing 0.3 mg/mL glutamine, 100 IU/mL penicillin, and 100 μg/mL streptomycin supplemented with 10% fetal calf serum (FCS) and 400 μg/mL Geneticin (Thermo Fisher Scientific). The MDA-MB-134 cells were cultured in DMEM high glucose-pyruvate medium supplemented with 20% FBS and 1% penicillin-streptomycin. The MCF-7 cells were grown in MEM alpha medium containing 10% FBS, 1% sodium pyruvate, 1% sodium bicarbonate, 1% non-essential amino acids and 1% antibiotic-antimycotic. The T-47D, SK-BR-3, ZR-75-1, and BT-549 cell lines were grown in RPMI-1640 medium containing 10% FBS and 1% antibiotic-antimycotic. For SK-BR-3 cells, medium additionally contained 1% sodium pyruvate and for BT-549, 1 μg/mL insulin. The BT-474 cells were grown in Hybri-Care medium containing 10% FBS, 1% sodium bicarbonate, and 1% antibiotic-antimycotic. The SUM44PE, SUM52PE, and SUM159 cells were grown in Ham’s F12 medium containing 1% antibiotic-antimycotic. For SUM159 cells, medium additionally contained 5% FBS, 1 μg/mL hydrocortisone, 5 μg/mL insulin, and 10 mM HEPES buffer. For the SUM52PE cells, the medium additionally contained 10% FBS, 10 mM HEPES buffer, 1 μg/mL hydrocortisone, and 5 μg/mL insulin. For SUM44PE cells, the medium contained 2% FBS, 1 g/L bovine serum albumin, 5 mM ethanolamine, 10 mM HEPES buffer, 1 μg/mL hydrocortisone, 5 μg/mL insulin, 50 nM sodium selenite, 5 μg/mL apo-Transferrin, and 10 nM triiodothyronine (T3). The MCF-12A cells were cultured in 50% Ham’s F12 medium and 50% DMEM medium containing 5% horse serum, 1% antibiotic-antimycotic and 20 ng/mL epidermal growth factor (EGF), 10 μg/mL insulin, 500 ng/mL hydrocortisone and 100 ng/mL of cholera toxin. For experiments involving non-genetically manipulated human mammary epithelial cells (HuMECs) (Fig. 1f), human primary mammary epithelial cells were purchased from ATCC (HMEC, PCS-600-010). These cells were grown in Mammary Epithelial Cell Basal Medium (ATCC) supplemented with the components of the Mammary Epithelial Cell Growth Kit (ATCC). For experiments involving genetically manipulated (lentivirally transduced) HuMECs (Supplementary Fig. 2a, c and d), the cell line hTERT-HME1 [ME16C] (ATCC CRL4010) was utilized. These cells were grown in MEGM^TM^ Mammary Epithelial Cell Growth Medium BulletKit^TM^ (Lonza), with supplements added according to the manufacturers’ instructions.

The primary adult human dermal fibroblasts cells were purchased from ATCC (HDFa, PCS-201-012, Supplementary Fig. 2c and Supplementary Data 6) and grown in Fibroblast Basal Medium supplemented with Fibroblast Growth Kit-Low Serum (ATCC) in accordance to manufacturers’ instructions. The 3T3-L1 cells were grown in DMEM high glucose-pyruvate medium containing 10% FBS and 1% antibiotic-antimycotic.

### Immunohistochemistry (IHC)

The IHC staining of AAMDC and RabGAP1L was performed on a commercially available breast cancer tissue microarray (TMA) (Biomax, #BR1503d) and it was automatized using the Ventana BenchMark ULTRA IHC/ISH staining module available at the Fiona Stanley Hospital, Perth, Australia. The slides were warmed to 75 °C for 4 min, deparaffinated, conditioned, and treated with peroxidase inhibitor. The slides were then incubated for 32 min at 36 °C with α-AAMDC antibody (PTD015, Santa Cruz Biotechnology, 1:100) and α-RabGAP1L antibody (Proteintech, 1:50). The slides were counter-stained with Hematoxylin for 4 min. An ultraView Universal DAB Detection Kit (Ventana) was used for chromogenic detection and visualization.

The IHC of MCF-7 xenograft tumors was performed on tissues harvested on day 24, fixed in 4% paraformaldehyde, embedded in paraffin blocks, and cut into 5 μm sections. All of the sections for IHC were deparaffinized and hydrated with graded concentrations of ethanol. The tumor sections were stained with α-Ki-67 (Cell Signaling Technology, 1:500), α−p-AKT S473 (Cell Signaling Technology, 1:100), total α−AKT (Cell Signaling Technology, 1:200), α-cMyc (Santa Cruz Biotechnology, 1:200), α-MTHFD1L (Cell Signaling Technology, 1:200) or α-AAMDC (Santa Cruz Biotechnology, 1:30) antibodies. Images were captured using a Nikon Ti inverted microscope at ×40 magnification unless specified otherwise.

### Immunocytochemistry (ICC)

These cells were fixed with 4% paraformaldehyde in phosphate-buffered saline (PBS), permeabilized in 0.025% Triton X-100, and blocked with normal goat serum. Anti-AAMDC antibody (PTD015, Santa Cruz Biotechnology, 1:100) in 10% normal goat serum was added to the cells and incubated overnight at 4 °C. Cellular peroxidase activity was blocked with 0.3% hydrogen peroxide in PBS. The cells were then incubated for 30 min at room temperature with a biotinylated α-rabbit secondary antibody from the LSAB IHC Kit (Rabbit IgG) (GeneCopoeia). Detection was performed with streptavidin-horseradish peroxidase (HRP) for 30 min at room temperature, followed by 3,3-diaminobenzidine (DAB) solution for signal development. Positive AAMDC cells were detected under a light microscope Olympus IX71 (Singapore).

### Fluorescence in situ hybridization

FISH was performed using commercially available probes (Empire Genomics) labeled red (Red-dUTP 5-carboxyl-x-rhodamine) for the *AAMDC* gene, and green for the pericentromeric region of chromosome 11 (5-Fluorescein dUTP). After deparaffinization and rehydration, the slides were soaked in pre-treatment solution (Histology FISH Accessory Kit, Dako) and heated in a microwave oven for 10 min, followed by 17 min of pepsin digestion (room temperature, RTU pepsin solution, Histology FISH Accessory Kit, Dako). The slides were next dehydrated and 10 μL of probe working solution was applied to each slide. After co-denaturation at 78 °C for 5 min, the slides were placed overnight in a humid chamber at 37 °C. The fluorescence Mounting Medium (Histology FISH Accessory Kit, Dako) was applied prior to visualization with a fluorescence microscope (ZEISS, Germany). The FISH samples were analyzed using Isis Fluorescence Imaging (MetaSystems, Germany). For each sample, at least 40 nuclei were counted. Cells with a ratio of gene-specific probe (*AAMDC*) to control probe (pericentromeric region of chromosome 11) ≥2.0 were considered to have amplification.

### Molecular cloning

A fragment encoding residues 471–864 (*N-3xHA-RabGAP1LΔPTB*(471) of full-length *N-3xHA-RabGAP1L WT* and a fragment encoding residues 1–585 (*N-3xHA-RabGAP1LΔTBC*(585)) were amplified by PCR from the pEZ-M06 *N-3xHA-RabGAP1L WT* plasmid (GeneCopoeia) and were subcloned into the Pme1 and Not1 restriction sites of pEZ-M06 *N-3xHA-RabGAP1L WT* (GeneCopoeia). For the *N-3xHA-RabGAP1LΔTBC*(585) amplification, the primers used were: Forward 5′-CAGCCTCCGGACTCTAGC-3′ and Reverse 5′-CTCCTCCTCCTCCTCCTCCTCGCGGCCGCACTCGAGCTACACCAGAGTAGACAGCCCTTT-3′. For the *N-3xHA-RabGAP1LΔPTB*(471) amplification, the primers were: Forward 5′-GAGGAGGAGGAGGAGGAGGTTTAAACCGATTTTGGTATTTCAGCAG-3′ and Reverse 5′- GCTTATAATACGACTCACTATAGGG-3′, as described in Supplementary Table 2. These transient expression vectors were used in all the IPs (Fig. 8, Supplementary Fig. 6), intracellular localization studies (Supplementary Fig. 8b), and BRET assays (Fig. 8g and Supplementary Fig. 8a).

The full-length *RabGAP1L WT* and the *RabGAP1L*Δ*PTB*(471) inserts were subcloned into the pLv105 lentiviral vector (Supplementary Fig. 9b–d). The *AAMDC-DAAE* mutant was generated by site-directed mutagenesis of the PTB binding consensus sequence, 20-NTTY-23 on pLv105*-AAMDC* (Supplementary Fig. 9b).

For BRET assays, the *AAMDC* cDNA was N-terminally fused to the bioluminescent donor *RLuc8* (*RLuc8-AAMDC*), the *Rab GTPases* were N-terminally fused to the *Venus* acceptor fluorophore (*Venus-Rab*), and *RabGAP1L* (full-length and mutants, see above) were not fused with any reporter activity. The *Venus-HA-Rab7a* expressing plasmid (in the pEYFP-C1 backbone, Clontech) was kindly provided by Nevin Lambert (Georgia Regents University). The *Venus-Rab13*, *Venus-Rab22a*, and *Venus-Rab35* fusions were synthesized by GeneArt (Thermo Fisher Scientific) and subcloned into a pcDNA3*-Venus* expressing plasmid, while the *RLuc8-AAMDC* insert was synthesized by GeneArt and subcloned into the pcDNA3*-RLuc8* vector^56^.

### Lentiviral production and transduction of breast cell lines

Lentiviral particles expressing either the shRNAs or the cDNAs were generated by lentiviral transduction of HEK293T cells transfected with 4.5 μg of either empty vector plasmids (pLKO.1 or pLv105, GeneCopoeia, Supplementary Data 6), pLKO.1 expressing the corresponding shRNA, or pLv105 expressing the selected cDNAs, and 1.54 μg of pCI VSV-G Envelope plasmid, 2.88 μg of pMDLg/pRRE (GAG/POL), and 1.1 μg of pRSV-Rev packaging plasmids (Addgene). The DNAs were mixed with 50 μL of Lipofectamine2000 transfection reagent (Thermo Fisher Scientific) in Opti-MEM Reduced Serum Medium (Gibco). Four hours after transfection, the HEK293T culture medium was replaced with a complete serum-containing medium. The supernatants containing the viral particles were harvested 44, 51, and 68 h after the medium replacement, and filtered through a 0.22 μm syringe filter (Millipore). The Polybrene (Sigma) was added to the filtered viral supernatants at a final concentration of 8 μg/mL.

### Transduction of shRNA- and cDNA-expressing lentiviruses

The host MCF-7, T-47D, SUM52PE, HuMEC, and MDA-MB-134 cells were seeded in 10 cm dishes and transduced with the lentiviral supernatants for a period of 8 h. The lentiviral transductions were identically repeated three times by collecting the HEK293T supernatants daily. Eight hours after the last transduction, the culture medium was replaced with a fresh medium. Seventy-two hours later, transduced cells were selected with 5 μg/mL of puromycin (Gibco) for the T-47D, 1.5 μg/mL for the HuMEC, 1.25 μg/mL for the SUM52PE, and 1 μg/mL for the MCF-7 and the MDA-MB-134 cell lines, respectively.

### Double lentiviral transductions

For rescue experiments demonstrating the on-target specificity of the *AAMDC* shRNA2, SUM52PE cells were initially transduced with a first lentiviral vector, either pLKO.1 or the *AAMDC* shRNA2 (see lentiviral transduction section above). Seventy-two hours post-transduction, the host cells were selected with 1.25 μg/mL of puromycin. Immediately after selection, the cells were transduced with the second lentivirus expressing either the empty vector pLv105 or the *AAMDC* cDNA (Supplementary Fig. 1c). Similarly, double transductions were performed as in Supplementary Fig. 3a (transduction #1: either pLKO.1 or the *TSC2* shRNA1; transduction #2: either pLKO.1 or the *AAMDC* shRNA2) and Supplemental Fig. 9c (transduction #1: either pLv105 or *RabGAP1L*Δ*PTB*; transduction #2: either pLKO.1 or the *AAMDC* shRNA2). These cell lines were maintained in cell media containing 1.25 μg/mL of puromycin. The efficacy of each individual consecutive transduction was validated by western blotting.

### Transient transfection assays

For the IP assays, HEK293T cells were transiently transfected with various combinations of plasmid constructs expressing: *N-Myc-Rab7a, N-Myc-Rab13, N-Myc-Rab22a, N-Myc-Rab35* (in pEZ-M43, GeneCopoeia)*, N-3xHA-RabGAP1L WT* (in pEZ-M06, GeneCopoeia), and *N-3xFLAG-AAMDC* (in the pcDNA3.1 (+) Zeo backbone, Invitrogen) (Fig. 8d–f and Supplementary Fig. 6). In addition, HEK293T cells were transfected with plasmid constructs expressing the mutants of *RabGAP1L*: *N-3xHA-RabGAP1L*Δ*PTB* (RabGAP1L with deletion of residues 1–471 defining the PTB domain) and *N-3xHA-RabGAP1L*Δ*TBC* (RabGAP1L with deletion of residues 585–815 comprising the TBC domain) (in pEZ-M06, see molecular cloning, Supplementary Data 6).

For in vivo real-time fluorescence imaging (Supplementary Fig. 7a) HEK293T cells were transiently transfected with *N-*e*GFP-AAMDC* (cloned in the pcDNA3.1 (+) Zeo backbone, Invitrogen)*, N-eGFP-Rab7a*, *N-eGFP-RabGAP1L* (in pEZ-M29, GeneCopoeia), an*d mCherry-2xFYVE*_*Hrs*_ construct (generously provided by A/Prof Rohan Teasdale from The University of Queensland and generated by subcloning *2xFYVE*_*Hrs*_ from pEGFP*-2xFYVE*_*Hrs*_ into the pmCherry-C1 backbone, Clontech). For IF assays on fixed cells (Supplementary Fig. 6f) HEK293T cells were transfected with *Ankyrin-B-2xHA* (in the pEGFP-N1 backbone, Addgene) or *N-3xFLAG-AAMDC* (in the pcDNA3.1 (+) Zeo backbone, Invitrogen).

To assess the intracellular localization of RabGAP1L and its mutants, and that of Rab7a (Supplementary Fig. 8b), the HEK293T cells were co-transfected with: *N-Myc-Rab7a* and full-length *N-3xHA-RabGAP1L WT* or its deletion mutants *N-3xHA-RabGAP1L*Δ*PTB* or *N-3xHA-RabGAP1L*Δ*TBC*.

The transfections were performed using Lipofectamine2000 reagent (Thermo Fisher Scientific) according to the manufacturer’s protocol. For the IP experiments, HEK293T cells were transfected with 10 μg of plasmid DNA, and for the IF localization experiments, the cells were seeded onto 13 mm glass coverslips pre-treated with poly-L-lysine prior to transfection with 0.66 μg of plasmid DNA. The transfection media was replaced by complete serum-containing media 4 h after transfection. For the IP experiments, total protein extract was collected 48 h post-transfection, while for IF, the cells were fixed in 4% paraformaldehyde 24 h post-transfection.

For the BRET assays, the HEK293FT cells were transfected with various combinations of *Venus-HA-Rab7a, Venus-Rab13, Venus-Rab22a* and *Venus-Rab35, RLuc8-AAMDC, RabGAP1L WT, RabGAP1L*Δ*PTB*, and *RabGAP1L*Δ*TBC* (Fig. 8g and Supplementary Fig. 8a). The transfections were carried out using FuGENE 6 transfection reagent (Promega). The cells were harvested with 0.05% trypsin-EDTA 24 h after transfection and seeded into poly-L-lysine (Sigma Aldrich)-coated white 96-well plates in phenol red-free DMEM containing 25 mM HEPES, 0.3 mg/mL glutamine, 100 IU/mL penicillin, and 100 μg/mL streptomycin supplemented with 5% FCS.

### Quantitative real-time PCR (RT-qPCR)

Total RNA was extracted from tissue and transfected and transduced cells using QIAzol Lysis Reagent (Qiagen). The tumor tissue was homogenized at 30 Hz, 30 s cycles using a Qiagen TissueLyser II. cDNA was synthesized using the High-Capacity cDNA Reverse Transcription Kit (Applied Biosystems). Relative quantification of transcript expression was obtained by RT-qPCR using TaqMan probes in the ViiA^TM^ 7 RT-PCR machine (Applied Biosystems). The mRNA levels of *glyceraldehyde 3-phosphate dehydrogenase (GAPDH), Peptidylprolyl Isomerase A (PPIA)*, and *Glucuronidase Beta (GUSB)* were used for normalization. The *AAMDC* mRNA levels in a panel of breast cancer cell lines were expressed as fold change relative to that of MCF-12A cells after normalization with the housekeeping genes.

### Ki-67 proliferation and cleaved caspase-3 marker detection by immunofluorescence

The untransduced T-47D, MDA-MB-134, HuMEC, and SUM52PE cells or the cells lentivirally transduced with the empty vector pLKO.1 or with *AAMDC* and *MTHFD1L* shRNAs were seeded onto 13 mm glass coverslips pre-treated with poly-L-lysine (Sigma-Aldrich). The adhered cells were fixed with 4% paraformaldehyde for 20 min, washed twice with PBS, blocked with 5% normal goat serum for 1 h, and separately incubated with α-Ki-67 mouse monoclonal primary antibody (Cell Signaling Technology, 1:500) and with α-cleaved caspase-3 (Cell Signaling Technology, 1:500) in antibody diluent (1% BSA and 0.3% Triton X-100 in PBS) overnight and further incubated with a goat α-mouse secondary Alexa Fluor 488-conjugated antibody (Thermo Fisher Scientific, 1:500) and goat α-rabbit secondary Alexa Fluor 488-conjugated antibody (Thermo Fisher Scientific, 1:500), respectively. The percentage of Ki-67 and cleaved caspase-3 positive cells versus total cells (Hoechst 33258, 1:5000) was assessed in at least 9 fields of view. Data were normalized to empty vector (EV) transduced cells.

### Immunofluorescence assays

Immunofluorescence (IF) experiments for assessing the cellular localization of endogenous AAMDC and Rab-GTPase activating protein 1 like (RabGAP1L) proteins were performed in selected luminal breast cancer cell lines (Fig. 8a). The IF experiments for assessing the localization of phosphorylated-mammalian target of rapamycin serine 2448 (p-mTOR S2448), tuberous sclerosis complex 2 (TSC2), p110α with lysosome-associated membrane protein 2 (LAMP2), early endosome antigen 1 (EEA1) (Supplementary Fig. 3b), and FGFR2 (Supplementary Fig. 7b) were performed in SUM52PE cells transduced with either empty vector (pLKO.1) or *AAMDC* sh2. The cells were seeded overnight in 13 mm glass coverslips pre-treated with poly-L-lysine (Sigma-Aldrich). Subsequently, the cells were fixed with 4% paraformaldehyde, blocked with 5% normal goat serum, and incubated overnight at 4 °C with the primary antibodies α-AAMDC mouse monoclonal (Abcam, 1:30), α-RabGAP1L rabbit polyclonal (Proteintech, 1:50), α-phospho-mTOR (Ser2448) rabbit monoclonal (Cell Signaling Technology, 1:50), α-TSC2 rabbit monoclonal (Cell Signaling Technology, 1:800), α-PI3 Kinase p110α rabbit monoclonal (Cell Signaling Technology, 1:100), α-LAMP2 mouse monoclonal (Santa Cruz Biotechnology, 1:100), α-EEA1 rabbit polyclonal (Cell Signaling Technology, 1:100), and α-FGFR2 rabbit monoclonal (Cell Signaling Technology, 1:100), diluted in antibody diluent (1% BSA and 0.3% Triton X-100 in PBS). The F-actin staining was performed using Alexa Fluor™ 488 Phalloidin (Thermo Fisher Scientific, 1:500, Fig. 2e and Supplementary Fig. 9d).

For IF experiments involving transiently transfected HEK293T cells, the following primary antibodies were used: α-Myc Tag rabbit monoclonal (Cell Signaling Technology, 1:200), α-HA Tag antibody rat monoclonal (Novus Biologicals, 1:100), and α-FLAG (DYKDDDDK) Tag (9A3) mouse monoclonal (Cell Signaling Technology, 1:1600) (Supplementary Fig. 6f and 8b). The cells were then incubated for 1 h at room temperature with the following secondary antibodies: goat α-mouse or α-rabbit 488-conjugated antibody, goat α-mouse or α-rabbit 594-conjugated antibody, and goat α-rat 647-conjugated antibody (Thermo Fisher Scientific, 1:500). The cells were incubated with Hoechst 33258 (Sigma-Aldrich, 1:5000) for nuclei staining for 15 min. The coverslips were mounted onto slides and imaged using a Nikon A1Si inverted confocal microscope.

For the live cell imaging (Supplementary Fig. 7a), HEK293T cells transiently cotransfected with *mCherry-2xFYVE* and either *eGFP-Rab7a*, e*GFP-RabGAP1L or eGFP-AAMDC*, were grown on glass-bottom culture dishes (35 mm petri dish, 10 mm microwell with no. 1.5 coverglass, MatTek Corporation) and imaged by time-lapse fluorescence confocal microscopy using an inverted 100× (CFI Plan Apo Lambda Oil, N.A. 1.45) objective lens under controlled atmospheric conditions (37 °C and 5% CO_2_/Air) in a Tokai Hit Stage Top incubator (INUG2E-TIZ, Fujinomiya-shi, Shizuoka-ken, Japan). Frames were captured continuously for 5 min using NIS-C Elements AR (Version 4.13) software and processed using ImageJ.

### Anchorage-independent colony formation assays

Anchorage-independent growth was assessed with soft agar colony formation assays. The HuMEC, HDFa, T-47D, SUM52PE, or MDA-MB-134 cells were seeded in six-well plates, in 0.48% agarose in complete medium (Sea Plaque Low-Melting Agarose, Lonza), over a solid layer of 0.8% agarose in complete medium. Five-hundred microliters of complete growth medium were added to each well and replaced every 3 days. The colonies were allowed to grow for 3–4 weeks. The colonies were stained with methylthiazolyldiphenyl-tetrazolium bromide (MTT) (Sigma) at 0.5 mg/mL in complete media, washed in PBS and counted by Leika light microscope in 12 random fields of view.

### Induction of the PI3K-AKT-mTOR pathway

For cell signaling studies, SUM52PE cells were serum- and insulin-starved overnight and then treated with insulin (Ins, 300 μg/mL), 20% FBS, or tumor necrosis factor α (TNFα, 50 ng/mL) for 24 h or left untreated before being lysed for immunoblotting. For amino acid induction, SUM52PE cells were serum- and insulin-starved overnight and then maintained in Dulbecco’s phosphate-buffered saline (DPBS) for 1.5 h to remove the amino acids from the cell medium, followed by induction by adding 2× solution containing non-essential (100× stock) and essential (50× stock) amino acids (Gibco). Four hours post-induction, the cells were lysed for immunoblotting. Cells maintained in DPBS were used as mock controls.

For estrogen induction, SUM44PE or T-47D cells were first cultured for 72 h in estrogen-free medium (for SUM44PE: Ham’s F12 medium without phenol red, supplemented with the following agents (at their final concentrations): 2% charcoal-stripped FBS, 1 g/L bovine serum albumin, 5 mM ethanolamine, 10 mM HEPES buffer, 1 μg/mL hydrocortisone, 5 μg/mL insulin, 50 nM sodium selenite, 5 μg/mL apo-Transferrin, 10 nM triiodo-thyronine (T3), and 1% antibiotic-antimycotic; for T-47D: RPMI-1640 medium without phenol red, supplemented with 10% charcoal-stripped FBS and 1% antibiotic-antimycotic). The cells were then seeded in estrogen-free medium, incubated overnight, and estrogen (Sigma-Aldrich) (1 nM) was added for 15, 30, 60, 120 min, 7 h, and 24 h (SUM44PE) or 24 h (T-47D) before lysis for immunoblotting.

### Bioluminescence energy resonance transfer (BRET) assay

Forty-eight hours post-transfection of the HEK293FT cells with the corresponding plasmids (see transient transfection assays), the DMEM complete media was replaced with 5 μM coelenterazine *h* (Promega) in calcium- and magnesium-free Hank’s Balanced Salt Solution (HBSS). The BRET measurements were immediately taken at 37 °C using a LUMIstar Omega plate reader (BMG Labtech Mornington, Vic, Australia). Filtered light emissions were simultaneously measured at 460–490 nm for RLuc8 and 520–550 nm for Venus. The BRET ratio was calculated by subtracting the ratio of 520–550 nm emission and the 460–490 nm emission^57,58^.

### Induction of 3T3-L1 pre-adipocytes into mature adipocytes

The 3T3-L1 mouse embryonic fibroblasts were treated with complete medium supplemented with MDI reagents (2 μM insulin, 1 μM dexamethasone, 0.5 μM 3-isobutyl-1-methylxanthine) to induce adipocytic differentiation for 48 h. The medium was then replaced with a complete medium supplemented with 2 μM insulin for a further 48 h before Oil Red O staining.

### Oil Red O staining of neutral lipids

For the quantitative assessment of neutral lipid formation, the HuMEC or SUM52PE cells lentivirally transduced with the *AAMDC* cDNA or the AAMDC shRNA2 and controls (Supplementary Fig. 2d) were washed in PBS, fixed with 4% paraformaldehyde for 30 min, washed in distilled water, and briefly incubated in 40% isopropanol. Following the equilibration, the cells were stained with 40% Oil Red O solution for 20 min and counterstained with hematoxylin. Coverslips were mounted onto slides and imaged with a Nikon Ti inverted microscope. Images were collected using NIS-Elements AR (Version 4.13) software and processed using ImageJ.

### Migration assays

Migration assays were performed similarly to Moses et al.^59^. Transwell polycarbonate membrane filter inserts with 8 μm pore size (Cell Biolabs) were rehydrated and placed in 24-well tissue culture plates. MCF-7, T-47D, and SUM52PE cells were transduced with either *AAMDC* shRNAs or empty vector pLKO.1. The cells were serum-deprived overnight and added to the top chambers of the transwells. Ten percent serum-containing media was added to bottom chambers. The plates were next incubated at 37 °C for 24 h and then the culture media was aspirated. To visualize the migratory cells, the inserts were incubated with 400 μL cell stain solution (Cell Biolabs) for 10 min and pictures were captured using a cell culture inverted Leika light microscope to quantitate the number of cells that had passed through the porous membrane in 12 random fields. Results were expressed as average values relative to the empty vector pLKO.1 cells.

### In vivo tumor growth assay

For the generation of the *AAMDC* shRNA KD xenografts, 5 × 10^6^ T-47D cells stably transduced with either pLKO.1 lentiviral empty vector or *AAMDC* sh2 were injected subcutaneously into the right flank of 5-week-old female BALB/cJ Foxn1/Arc (Nude) mice. Prior to inoculation, the cells were resuspended in 100 μL of a 1:1 solution of Ham’s F12 medium and BD Matrigel® High Concentration (BD Bioscience). One microgram of estradiol valerate dissolved in 50 μL of peanut oil (Sigma-Aldrich) was injected subcutaneously in the proximity of the tumor every 4 days to allow the estrogen-dependent T-47D cells to proliferate. The widths and the lengths of the tumors were measured every 3–4 days using digital calipers for 14 days. Tumor volumes were calculated using the modified ellipsoid formula; *V* = [(width^2^) × ½ × length]. Animals bearing tumors >800 mm^3^ were humanely sacrificed.

For the *AAMDC* cDNA overexpression in xenografts, 1 × 10^6^ MCF-7 cells stably transduced with either pLv105 lentiviral empty vector or *AAMDC* cDNA were injected subcutaneously into the right flank of 5-week-old female BALB/cJ Foxn1/Arc mice. Twenty-four hours prior to inoculation, the cells were placed into minimal essential medium (MEM) and then resuspended in 100 μL of a 1:1 solution of MEM and Matrigel® Growth Factor Reduced (GFR) phenol red-free (Corning) for injection. +E2 mice were given injections of estradiol valerate every 3–4 days. The mice were monitored and the tumor sizes were measured as described above.

### RNA sequencing (RNA-seq)

Total RNA was extracted from SUM52PE cells lentivirally transduced with the empty vector pLKO.1 and *AAMDC* sh2 according to the instructions of the RNeasy Mini Kit (Qiagen). For the drug-treated samples, SUM52PE cells were treated twice every 36 h with dactolisib, everolimus, or AZD8055 (Selleck Chem, 100 nM) or buparlisib (Selleck Chem, 1000 nM), or vehicle control. After 72 h, total RNA was extracted from the cells using QIAzol Lysis Reagent (Qiagen) and the samples were DNase treated with the TURBO DNA-*free*™ Kit (Thermo Fisher Scientific).

Eluted RNA samples were sequenced using the HiSeq 2500 System at the Australian Genome Research Facility (AGRF) in Melbourne, with Illumina’s TruSeq stranded mRNA sample preparation protocol used for library preparation. The RNA sequencing data have been deposited in the Gene Expression Omnibus public database under accession numbers GSE92893 and GSE123740.

### Western blotting

For protein extraction, the cells were washed twice with PBS and lysed in ice-cold Cell Lysis Buffer (Cell Signaling Technology) containing 1 mM phenylmethylsulfonyl fluoride (PMSF) then sonicated for 5 s at 10 mA. From each sample, 15 μg protein was resolved by sodium dodecyl sulfate-polyacrylamide gel electrophoresis (SDS-PAGE) and transferred to PVDF membranes (BioRad). Immunoreactivity was determined with primary antibodies (1:1000) as described in Supplementary Data 6, except for mouse monoclonal α-AAMDC (Abcam, 1:500), and secondary goat α-rabbit or α-mouse antibodies (1:10,000). The membranes were visualized by Novex^TM^ ECL HRP Chemiluminescent Substrate Reagent Kit (Thermo Fisher Scientific) or Luminata crescendo (MilliporeSigma™) using the ChemiDoc^TM^ Imaging System (Bio-Rad).

### IP experiments

The HEK293T cells were co-transfected for 48 h with various combinations of plasmids expressing the following tagged constructs: *Myc-Rab7a*, *Myc-Rab13*, *Myc-Rab22a*, *Myc-Rab35*, *FLAG-AAMDC*, *HA-RabGAP1L*, *HA-RabGAP1L*Δ*PTB, HA*-*RabGAP1L*Δ*TBC*, and *Ankyrin-B-HA* (see transient transfection assays, Supplementary Data 6). The cells were harvested and lysed by sonication in lysis buffer (Cell Signaling Technology) containing 1 mM PMSF and then pelleted by centrifugation. Two-hundred microliters of cell lysate were collected for each IP. Unconjugated beads and beads conjugated with IgG were utilized for controls; beads conjugated with Myc-Tag (Cell Signaling Technology), α-FLAG, and α-HA were used to immobilize the Myc-tagged Rab proteins, FLAG-tagged AAMDC, and HA-tagged RabGAP1L (full length and mutant proteins), respectively. Dynabeads™ Protein G (Thermo Fisher Scientific) were conjugated by incubation with PBST (beads only), IgG antibody (SCBT), or α-HA antibody (BioLegend) for 10 min at room temperature. Two-hundred microliters of lysate were incubated with 30 μL of beads for 10 min at room temperature with rotation for IgG, α-HA, and α-MYC IPs, and overnight at 4 °C for FLAG-conjugated beads. The beads were washed three times with TBST or TBS, and bound protein was eluted in 50 μL of SDS-PAGE buffer and separated directly by SDS-PAGE.

### Comprehensive metabolomics

Metabolomics was performed using liquid chromatography-mass spectrometry (LC-MS). Cultured cells (1.5 × 10^6^ cells/sample) were washed three times with PBS, pelleted by centrifugation, and then stored at −80 °C. Cell pellets were resuspended in 100 μL of ice-cold 80:20 methanol:water and lysed by three freeze-thaw cycles. An additional 300 μL of ice-cold 80:20 methanol:water was added, and the samples were sonicated and centrifuged at 14,000 × *g* for 10 min at 4 °C. The supernatants (375 μL) were dried using a vacuum concentrator. The samples were re-suspended in 50 μL of 80:20 methanol:water and then sonicated, vortexed, and centrifuged at 14,000 × *g* for 10 min at 4 °C. An injection volume of 2.5 μL, which contained metabolic material from ~75,000 cells, was used for all of the cell samples. LC-MS/MS-based metabolomics analysis was performed using a Thermo Q Exactive Orbitrap mass spectrometer coupled to a Thermo Vanquish UPLC system. Chromatographic separation of metabolites was achieved using a Millipore (Sequant) Zic-pHILIC 2.1 × 150 mm 5 μm column maintained at 25 °C. The collected data was imported into mzMine 2.20 software suite for analysis. Pure standards were used for the identification of metabolites through manual inspection of spectral peaks and matching of retention time and MS1 accurate mass, with confirmation of the identification through comparison to MS/MS fragmentation patterns.

### Yeast two-hybrid screenings

The yeast two-hybrid screenings were carried out by Hybrigenics Services, S.A.S., Paris, France. The *Homo sapiens AAMDC* coding sequence (https://www.ncbi.nlm.nih.gov/nuccore/34328078) was cloned into the pB27 vector as a C-terminal fusion with the LexA DNA-binding domain (*N-LexA-AAMDC-C*), and into the pB66 backbone as a C-terminal fusion with the DNA-binding domain of Gal4 (*N-Gal4-AAMDC-C*). These constructs were utilized as baits for the screening of a human cDNA library generated by random priming from the T-47D, MDA-MB-468, MCF-7, and BT-20 breast cancer cell lines. The library was next cloned into the pP6 vector.

Eighty-five million clones (eightfold the complexity of the library) and 68 million clones (sixfold the complexity of the library) were screened with the LexA and the Gal4 constructs, respectively. A mating approach with the YHGX13 (Y187 ade2–101::loxP-kanMX-loxP, matα) and the L40Δgal4 (mata) yeast strains were employed for the LexA screenings. Similarly, a mating method with the HGX13 (Y187 ade2–101::loxP-kanMX-loxP, matα) and the CG1945 (mata) yeast strains was performed for the Gal4 screenings. A total of 16 His+ colonies were selected on a medium without tryptophan, leucine, and histidine, while 53 His+ colonies were selected on a medium lacking tryptophan, leucine, and histidine, respectively. The positive clones (prey fragments) were PCR-amplified and sequenced at their 5′ and 3′ junctions (Supplementary Data 5). The resulting sequences were used to identify the corresponding interacting proteins in the GenBank database (NCBI) by a fully automated procedure. A confidence score (PBS, for Predicted Biological Score) was attributed to each interaction^60^.

### Chromatin immunoprecipitation (ChIP)

ChIP experiments were performed in biological triplicates. For each ChIP sample, SUM52 cells were seeded in 1 × 15 cm^2^ tissue culture plate with a final confluence of >80% and treated with either PBS (vehicle) or 100 nM dactolisib. In addition, SUM52 cells transduced with empty vector pLKO.1 or *AAMDC* shRNA2 were also seeded in the same conditions. These cells were fixed for 10 min at room temperature with 20 mL complete media containing 1% paraformaldehyde (Thermo-Fisher). One mL of 2.5 M glycine was added to each plate to quench unreacted formaldehyde. The media was aspirated and the fixed cells washed twice with 1× ice-cold PBS containing 1 mM PMSF. The cells were aseptically scraped using cell scrapers (Sarstedt) and pelleted at 1150 × g for 4 min at 4 °C. The cell pellets were washed twice in 1× ice-cold phosphate-buffered saline (PBS) and resuspended in 1 mL sodium dodecyl sulfate (SDS) Lysis Buffer (1% SDS, 10 mM ethylenediaminetetraacetic acid (EDTA), 50 mM Tris-Cl pH 8.1 in sterile milliQ water). The samples were sonicated using a Covaris M220 instrument at 4 °C to fragment chromatin into 200–1500 bp segments. Following sonication, equal amounts of ChIP dilution buffer (0.1% SDS, 1% Triton X-100, 2 mM EDTA, 20 mM Tris-Cl pH 8.1, 150 mM NaCL in sterile milliQ water) was added to each sample. The insoluble components were removed by centrifugation at 15,500 × *g* for 10 min at 4 °C. Sheared chromatin was conjugated with α-c-MYC-antibody (Cell Signaling Technology, 1:50) and α-ATF4-antibody (Cell Signaling Technology, 1:100). The samples were incubated with the corresponding antibodies for 4 h on a rotator at 4 °C and no antibody samples were investigated as negative controls. Per each ChIP reaction, 200 μL Dynabeads™ Protein G (Invitrogen) were washed twice with ChIP dilution buffer and added to the sheared chromatin-antibody samples. The conjugated samples and beads were incubated on a rotator overnight at 4 °C. After incubation, beads were washed for 5 min with a single wash of the following ice-cold buffers: low salt wash buffer (0.1% deoxycholate, 1% Triton X-100, 1 mM EDTA, 50 mM HEPES pH 7.5, 150 mM NaCL in sterile milliQ water), high salt wash buffer (0.1% deoxycholate, 1% Triton X-100, 1 mM EDTA, 50 mM HEPES pH 7.5, 500 mM NaCl) and LiCl wash buffer (250 mM LiCl, 0.5% NP40, 0.5% deoxycholate, 1 mM EDTA, 10 mM Tris-Cl pH 8.1). Beads were washed twice with TE Buffer (Invitrogen, pH 8.0) for 5 min for each wash. The immunoprecipitated chromatin was eluted from beads by incubation for 1 h at 60 °C in SDS elution buffer (1% SDS, 10 mM EDTA, 50 mM Tris-Cl pH 8.1) followed by a reverse cross-link incubation overnight at 70 °C. Samples were treated with RNase A and Proteinase K, and the DNA was purified using the phenol-chloroform phase separation DNA extraction method. The immunoprecipitated DNA was quantified using the Qubit 4 Fluorometer (Invitrogen).

### Chromatin immunoprecipitation coupled with quantitative PCR (ChIP-qPCR)

The ChIP-qPCR method enabled the amplification of the genomic regions containing TF binding sites in the proximal promoter of *MTHFD1L* and *ASNS*. The qPCR was performed with the QuantiFast SYBR Green PCR master mix (Qiagen) using a ViiA 7 Real-Time PCR System (Applied Biosystems). The amplification of exon 6 of the *Actin Beta* (*ACTB)* gene having no TF binding sites was assessed as control. Sequences of the promoter-specific oligonucleotide primers (Integrated DNA Technologies) used in this study were *MTHFD1L* Forward 5′-GTTTAGGGGCGATTTTGTGACCAC-3′, *MTHFD1L* Reverse 5′-TGATTGGTTCCAGGGCCCCTC-3′, *ASNS* Forward 5′-AGTCCTGCTCCGCCC-3′, and *ASNS* Reverse 5′-GCACGCGAGGAGGATGC-3′. Amplification of exon 6 of *ACTB* was performed with the following primers: *hActB*_ex6_Forward 5′-GATGAGATTGGCATGGCTTT-3′ and *hActB*_ex6_Reverse 5′-CACCTTCACCGTTCCAGTTT-3′ (Supplementary Table 2). The ChIP qPCR data were expressed as fold-increase of signal at the proximal promoters of *MTHFD1L* and *ASNS* normalized to the signal at the *ACTB* promoter and calculated for validated peak enrichment from three biological replicates of 100 nM dactolisib and sh2 against *AAMDC* vs. vehicle control and pLKO.1, respectively.

### Drug treatments and cell viability assays

For assessment of PI3K-AKT-mTOR pathway activity (Fig. 4b), the SUM52PE cells were treated with four different drug regimes: (1) dactolisib; (2) AZD8055; (3) everolimus at concentrations of 0, 1, 10, and 100 nM; and (4) buparlisib at concentrations of 0, 1, 10, 100, 500, and 1000 nM for a period of 24 h. For assessment of drug combinations on PI3K-AKT-mTOR pathway activity (Fig. 7a), the SUM44PE and MDA-MB-134 cells were treated with combinations of: (1) dactolisib; (2) everolimus (10 and 50 nM) and tamoxifen (5 μM) for a period of 24 h. Single drug treatments were used as controls. The cells were harvested in Cell Lysis Buffer (Cell Signaling Technology) for subsequent immunoblotting.

For insulin rescue experiments (Supplementary Fig. 3c), SUM52PE cells transduced with either empty vector (EV) or *AAMDC* shRNAs as well as untransduced cells (WT) were insulin starved overnight. Cells were vehicle treated or stimulated with insulin (300 μg/mL) for 7 h and then harvested in Cell Lysis Buffer.

For cell viability assays (Fig. 7b–e), the MDA-MB-134, SUM44PE, and T-47D cell lines were treated with single agents: (1) tamoxifen (0–20 μM); (2) dactolisib (0–200 nM); and (3) everolimus (0–200 nM); or in combination treatments of dactolisib or everolimus with different doses of tamoxifen. Cells were treated for a period of 72 h and processed with CellTiter-Glo® 2.0 luminescence assay protocol (Promega) to determine cell viability.

To investigate the influence of the *AAMDC* cDNA on the drug responses of MCF-7 cells, these cells were stably transduced with empty vector (EV) or overexpressing the *AAMDC* cDNA and treated with either: (1) dactolisib (0–200 nM); (2) everolimus (0–200 nM); and (3) docetaxel (1–100 nM) for 72 h and processed by CellTiter-Glo® 2.0 luminescence assay protocol (Promega). Luminescence was measured using the EnVision® Multilabel Plate Reader (PerkinElmer). The luciferase measurements were normalized to the vehicle condition (untreated).

### Quantification and statistical analysis

The *AAMDC* mRNA expression, Ki-67 immunofluorescence^61^, anchorage-independent cell growth, cell migration, cleaved caspase immunofluorescence, and dose-dependent changes in cell viability were normalized to the empty vector (EV). The relative *AAMDC* mRNA expression in a panel of ER^+^ breast cancer cell lines was normalized to the non-tumorigenic epithelial line MCF-12A. Unless otherwise indicated in the figure, the experiments were performed on at least three independent biological replicates. For the in vivo tumorigenicity of *AAMDC* sh2 KD, statistical significance was determined with *n* = 8 mice/group, while for MCF-7 *AAMDC* cDNA, *n* = 7 mice were analyzed for the +E2 EV group and *n* = 8 mice for other groups until day 24, when one mouse from each group was culled. The statistical analyses were performed using Prism 7 software for Mac OS X. For the analysis of differentially regulated metabolites by LC-MS, the experiments were performed on three independent biological replicates, and statistical significance was determined by an unpaired *t*-test. Metabolites were considered to be significantly regulated when the −2 < log_2_FC was <2 and the *p*-value was <0.05 (FC = fold change). For the RNA-seq data shown in Fig. 3, sequenced reads were aligned to the human (hg19) genome using TopHat (v2.0.14)^62^, and expression at the gene level (FPKM values) was estimated and normalized by Cufflinks (v2.2.1), Cuffmerge (v1.0.0), and Cuffnorm (v2.2.1). Differential gene expression analysis was performed using Cuffdiff (v2.2.1), with significant changes in gene expression determined using a *q*-value < 0.05 in three biological replicates. For the RNA-seq data shown in Fig. 5, the sequenced reads underwent pseudo-alignment against the GRCh38 (Ensembl 89) reference genome and quantification using Salmon (v0.8.2)^63^. The data were imported into R (v3.5) using the Bioconductor package tximport^64^ and collapsed to the gene-level for differential expression analysis using DESeq2 (v1.24.0)^65^. The results were visualized with python (v3.6) using the matplotlib^66^, SciPy (v1.3.0)^67^, NumPy (v1.16.4+mkl)^68^, and pandas (v0.24.2) packages^69^. All quantifications were done in at least 3–6 biological replicates and the mean ± SD is indicated in all of the quantifications. The Oil Red O staining experiments were performed with two independent biological replicates. The % neutral lipids were visually quantified relative to the total number of cells. The statistical analyses were conducted using Prism 8 software, with significance determined using an unpaired *t*-test.

### Software and algorithms

The image processing and quantification of the data were performed with NIS-Elements AR (Version 4.13) (Nikon Corporation, Tokyo, Japan) and ImageJ (Rasband, W.S., ImageJ, U. S. National Institutes of Health, Bethesda, Maryland, USA, https://imagej.nih.gov/ij/, 1997-2018). The DAVID (v6.8)^70^ database was used for GO functional annotation analysis. Structure analysis was conducted with PyMOL (v2.2) (Schrödinger)^71^ and the molecular modeling with Phyre2 (v2.0) (Structural Bioinformatics Group, Imperial College, London)^72^. The combination indexes were determined with the median dose-effect method (Chou and Talalay^73^) using CompuSyn software (ComboSyn, Inc.). The TMA visualization was carried out with Aperio ImageScope Pathology Slide Viewing Software (v12.3.3) (Leica Biosystems, Nussloch, Germany). Network analysis and KEGG pathway mapping of differentially regulated targets were performed by STRING (v11) (http://string-db.org) and DAVID (v6.8) (https://david.ncifcrf.gov/) databases, respectively. Lastly, GraphPad Prism (v8.4) was utilized for graphing and statistical analysis.

### Reporting summary

Further information on research design is available in the Nature Research Reporting Summary linked to this article.

## Supplementary information

Supplementary Information

Description of Additional Supplementary Files

Supplementary Data 1

Supplementary Data 2

Supplementary Data 3

Supplementary Data 4

Supplementary Data 5

Supplementary Data 6

Reporting summary

## Data Availability

The RNA sequencing data have been deposited and are available in the Gene Expression Omnibus (GEO) public database under accession numbers GSE92893 and GSE123740. Source data are provided with this paper. The authors declare that all data supporting the findings of this study are available within the article and its Supplementary Information files or from the corresponding author upon reasonable request. Source data are provided with this paper.

## Source data

Source Data

## Code availability

Code used to analyze ‘DepMap’ project data and to produce visualisations of RNA-seq data comparing specific drugs against shRNA-mediated *AAMDC* knockdown, are available from GitHub, https://github.com/jcursons/Golden_2021_NatComm.
