# Supplementary material for: The oncogene AAMDC links PI3K-AKT-mTOR signaling with metabolic reprograming in estrogen receptor-positive breast cancer
Source: Nat Commun. 2021 Mar 26;12:1920. doi: 10.1038/s41467-021-22101-7 (PMC7998036; doi:10.1038/s41467-021-22101-7)
Supplement: Supplementary file 1 — Supplementary Information [file 41467_2021_22101_MOESM1_ESM.pdf]

SUPPLEMENTARY INFORMATION

The oncogene *AAMDC* links PI3K-AKT-mTOR signaling with metabolic reprograming in estrogen receptor-positive breast cancer

Emily Golden[1,2*], Rabab Rashwan[1,2,3*], Eleanor A. Woodward[1,2*], Agustin Sgro[1,2,4], Edina Wang[1,2], Anabel Sorolla[1,2], Charlene Waryah[1,2], Wan Jun Tie[1,2], Elisabet Cuyàs[1,5,6], Magdalena Ratajska[7,8,9], Iwona Kardaś[7,10], Piotr Kozlowski[11], Elizabeth K. M. Johnstone[2,12,13], Heng B. See[2,12,13], Ciara Duffy[1,2,4], Jeremy Parry[14], Kim A. Lagerborg[15], Piotr Czapiewski[16,17], Javier A. Menendez[5,6], Adam Gorczyński[16], Bartosz Wasag[7,10], Kevin D. G. Pfleger[2,12,13,18], Christina Curtis[19], BumKyu Lee[20], Jonghwan Kim[21], Joseph Cursons[22], Nathan J. Pavlos[8,23], Wojciech Biernat[16], Mohit Jain[15], Andrew J. Woo[2,24], Andrew Redfern[25], and Pilar Blancafort[1,2,4,26]

[1]Cancer Epigenetics Group, The Harry Perkins Institute of Medical Research, The University of Western Australia, Perth, WA, Australia

[2]Centre for Medical Research, The University of Western Australia, Perth, WA, Australia

[3]Microbiology and Immunology Department, Faculty of Medicine, Minia University, Egypt

[4]School of Human Sciences, The University of Western Australia, Perth, WA, Australia

[5]Girona Biomedical Research Institute, Girona, Catalonia, Spain

[6]ProCURE (Program Against Cancer Therapeutic Resistance), Metabolism & Cancer Group, Catalan Institute of Oncology, Girona, Catalonia, Spain

[7]Department of Biology and Medical Genetics, Medical University of Gdansk, Gdansk, Poland

[8]The Centre for Cell Therapy and Regenerative Medicine, School of Biomedical Sciences, The University of Western Australia, Perth, WA, Australia

[9]Department of Pathology, Otago University, Dunedin, New Zealand

[10]Laboratory of Clinical Genetics, University Clinical Centre, Gdansk, Poland

[11]Institute of Bioorganic Chemistry, Polish Academy of Sciences, Poznan, Poland

[12]Molecular Endocrinology and Pharmacology, Harry Perkins Institute of Medical Research, Nedlands, WA, Australia

[13]Australian Research Council Centre for Personalised Therapeutics Technologies, Australia

[14]Department of Anatomical Pathology, Path West Laboratory, Fiona Stanley Hospital Network, Murdoch, WA, Australia

[15]Departments of Medicine and Pharmacology, University of California San Diego, CA, USA

[16]Department of Pathomorphology, Medical University of Gdansk, Gdansk, Poland

[17]Institute of Pathology, Dessau Medical Centre, Dessau, Germany

[18]Dimerix Limited, Nedlands, WA, Australia

[19]Stanford University School of Medicine (Departments of Medicine & Genetics) and Stanford Cancer Institute, Stanford, CA, USA

[20]Department of Biomedical Sciences, Cancer Research Center, University at Albany-State University of New York, Rensselaer, NY, USA

[21]Department of Molecular Biosciences, Center for Systems and Synthetic Biology, The University of Texas at Austin, Austin, TX, USA

[22]Biomedicine Discovery Institute & Department of Biochemistry and Molecular Biology, Monash University, Clayton, VIC, Australia

[23]School of Biomedical Sciences, The University of Western Australia, Perth, WA, Australia

[24]School of Medical and Health Sciences, Edith Cowan University, Perth, WA, Australia

[25]School of Medicine, University of Western Australia, Perth, WA, Australia

[26]The Greehey Children's Cancer Research Institute, The University of Texas Health Science Center at San Antonio, San Antonio, TX, United States of America

Corresponding author: Pilar Blancafort, Cancer Epigenetics Group, The Harry Perkins

Institute of Medical Research, Block QEII Medical Centre, 6 Verdun St, Nedlands WA 6009, Australia. Phone: +61 8 61510990; E-mail: pilar.blancafort@uwa.edu.au

*These authors contributed equally to this manuscript

Supplementary Figure 1

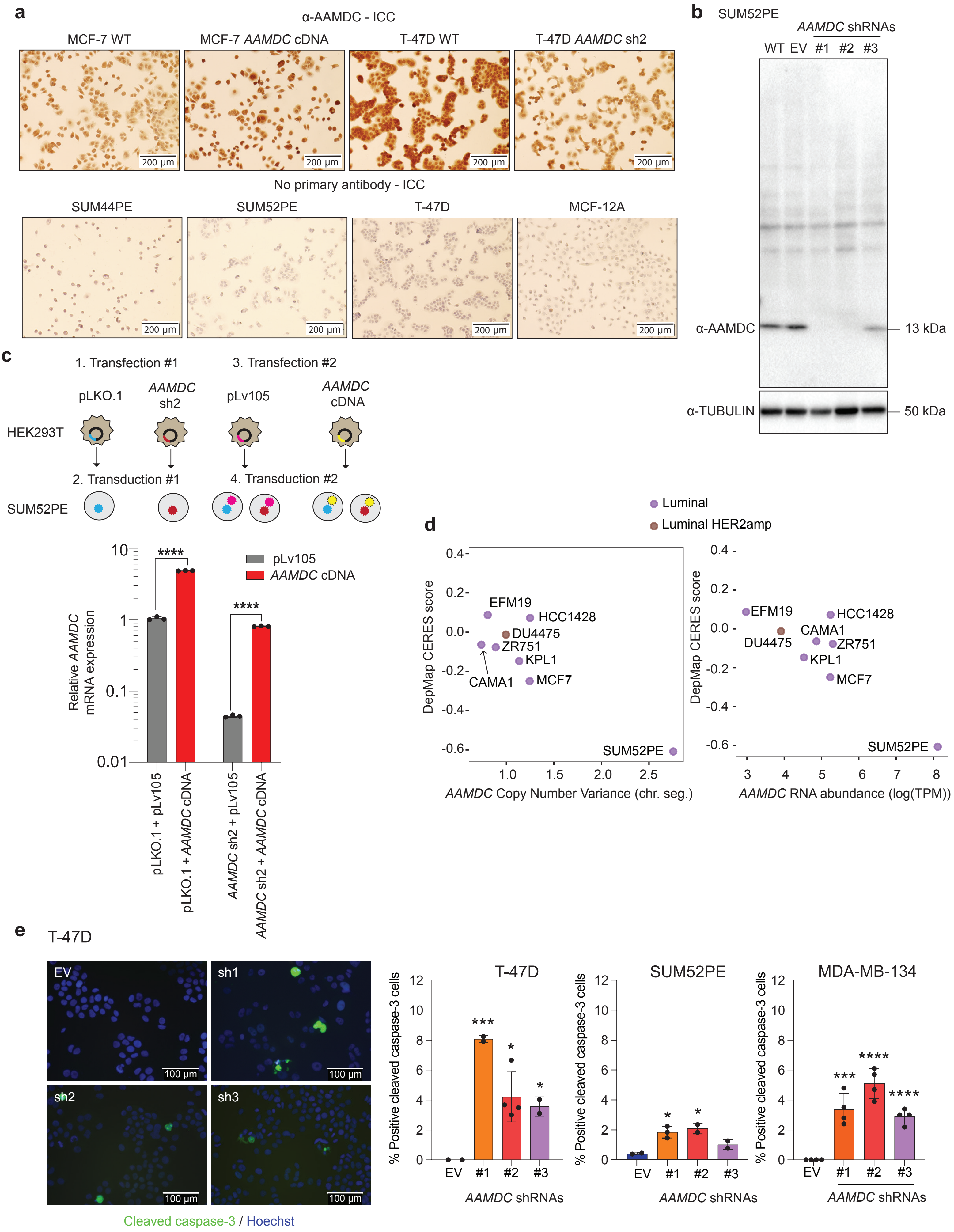

Supplementary Fig. 1. Relating to Fig. 1c: Specificity of AAMDC antibody (a-b). Relating to Fig. 2: On-target specificity of *AAMDC* shRNA (c-d) and depletion of *AAMDC* induces apoptosis in breast cell lines (e).

a, Validation of the rabbit polyclonal α-AAMDC antibody by immunocytochemistry (ICC). Detection of AAMDC by lentiviral *AAMDC* cDNA overexpression in MCF-7 cells (top left). Lentiviral transduction of *AAMDC* shRNA #2 (sh2) in T-47D cells (top right). ICC stains in the absence of AAMDC primary antibody (bottom).

b, Validation of the mouse monoclonal α-AAMDC antibody by immunoblotting (IB). SUM52PE cells were lentivirally transduced with either empty vector (EV) or with three different *AAMDC* shRNAs (Fig. 2). Source data are provided as a Source Data file.

c, On-target effects of the *AAMDC* knockdown (KD). Cells transduced with pLKO.1 empty vector EV or *AAMDC* sh2 were next transduced with pLv105 EV (grey bars) or with the *AAMDC* cDNA (red bars). The *AAMDC* mRNA expression relative to EV (pLKO.1 + pLv105) is indicated as mean values +/- SD, with statistical significance determined by two-tailed unpaired *t*-test on three replicates, ****$p < 0.0001$.

d, DepMap CRISPR knockout (KO) gRNA activity score (CERES score) of *AAMDC*-specific gRNAs in available luminal breast cancer cell lines from CCLE (Cancer Cell Line Encyclopedia). HER2amp, human epidermal growth factor receptor 2 amplification; Chr. Seg., chromosomal segment data; TPM, transcripts per million; WT, wild-type untransduced cells.

e, Cleaved caspase-3 immunofluorescence stains in breast cancer cell lines lentivirally-transduced with either empty vector (EV, pLKO.1) or *AAMDC* shRNAs. A representative image of cleaved caspase-3 staining (green) in T-47D cells transduced with EV or *AAMDC* shRNAs #1 - #3 (sh1-3) (left). Quantification of apoptotic cells as absolute percentage of caspase positive cells relative to all viable cells determined with Hoechst 33258 (blue) (right). EV (blue), shRNA #1 (orange), shRNA #2 (red), shRNA #3 (lilac). Data are presented as mean values +/- SD. *p*-values determined by two-tailed unpaired *t*-test (T-47D: *$p = 0.0282$ for sh2, *$p = 0.0164$ for sh3, ***$p = 0.0004$; SUM52PE: *$p = 0.0162$ for sh1, *$p = 0.0220$ for sh2; MDA-MB-134: ***$p = 0.0007$, ****$p < 0.0001$).

Supplementary Figure 2

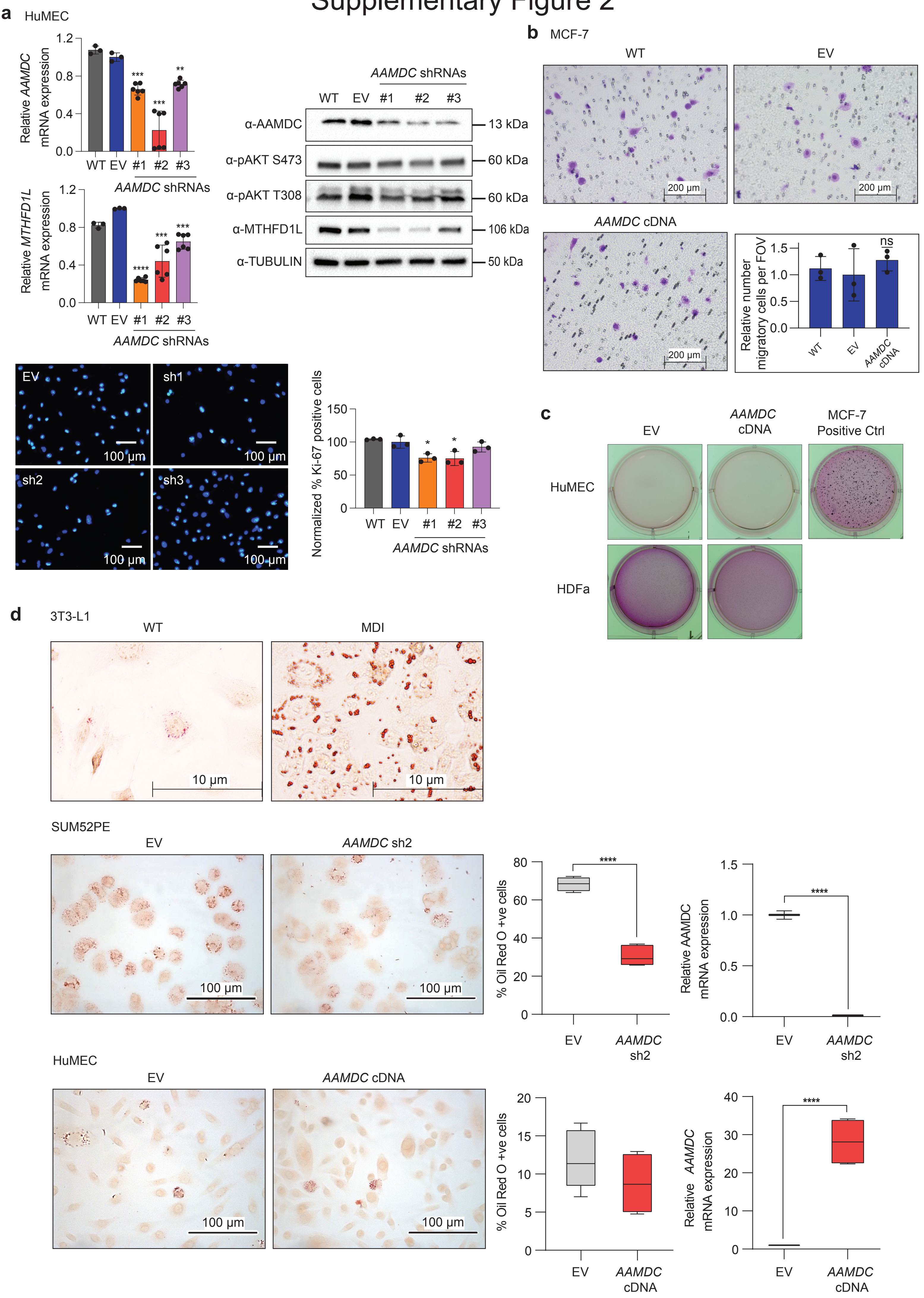

Supplementary Fig. 2. Relating to Fig. 2: Function of *AAMDC* in non-transformed and in poorly migratory cells.

a, Messenger RNA and protein expression and cell proliferation inhibition in human mammary epithelial cells (HuMEC) transduced with empty vector (EV) or with *AAMDC* shRNA #1 - #3 (sh1- sh3). Relative *AAMDC* and *MTHFD1L* mRNA expression (top left) normalized to EV and data presented as mean values +/- SD. *p*-values determined by two-tailed unpaired *t*-test (For *AAMDC*: $**p = 0.0029$, $***p = 0.0010$ for sh1, $***p = 0.0004$ for sh2; for *MTHFD1L*: $***p = 0.0009$ for sh2, $***p = 0.0002$ for sh3, $****p < 0.0001$). Immunoblots indicate AAMDC, MTHFD1L and PI3K pathway activation (top right). α-Ki-67 immunostaining (green), with nuclear Hoechst 33258 (blue) are indicated (bottom). Representative images are shown (left). Quantification performed with three biological replicates and normalized to EV (blue), shRNA #1 (orange), shRNA #2 (red), shRNA #3 (lilac), untransduced WT cells (grey). Statistical significance determined by two-tailed unpaired *t*-test as mean values +/- SD ($*p = 0.0212$ for sh1, $*p = 0.0384$ for sh2) (right). HuMEC, primary human mammary epithelial cells. Source data are provided as a Source Data file.

b, Cell migration by Boyden chamber assays in MCF-7 cells transduced with EV or *AAMDC* cDNA. Data presented as mean values +/- SD. *p*-values determined by two-tailed unpaired *t*-test. $n = 3$ biologically independent experiments. FOV, field-of-view; ns, not significant.

c, Colony formation assays with HuMEC cells and primary adult human dermal fibroblasts (HDFa) overexpressing EV or *AAMDC* cDNA. MCF-7 cells are shown as positive controls.

d, Lipid droplet formation by Oil Red O staining. Representative images of 3T3-L1 pre-adipocytes treated with MDI (0.5 μM 3-isobutyl-1-methylxanthine, 1 μM dexamethasone, 2 μM insulin) as a positive control (top). Representative images and plots indicating the percentage of Oil Red O^+ cells and *AAMDC* mRNA in SUM52PE cells transduced with *AAMDC* sh2 (red) or EV (grey) (middle); the same analyses in HuMEC cells (bottom). Statistical significance determined by a two-tailed unpaired *t*-test ($****p < 0.0001$) from $n = 3$ independent biological replicates. The boxplots indicate median, 25th, and 75th percentiles; whiskers correspond to minimum and maximum values.

Supplementary Figure 3

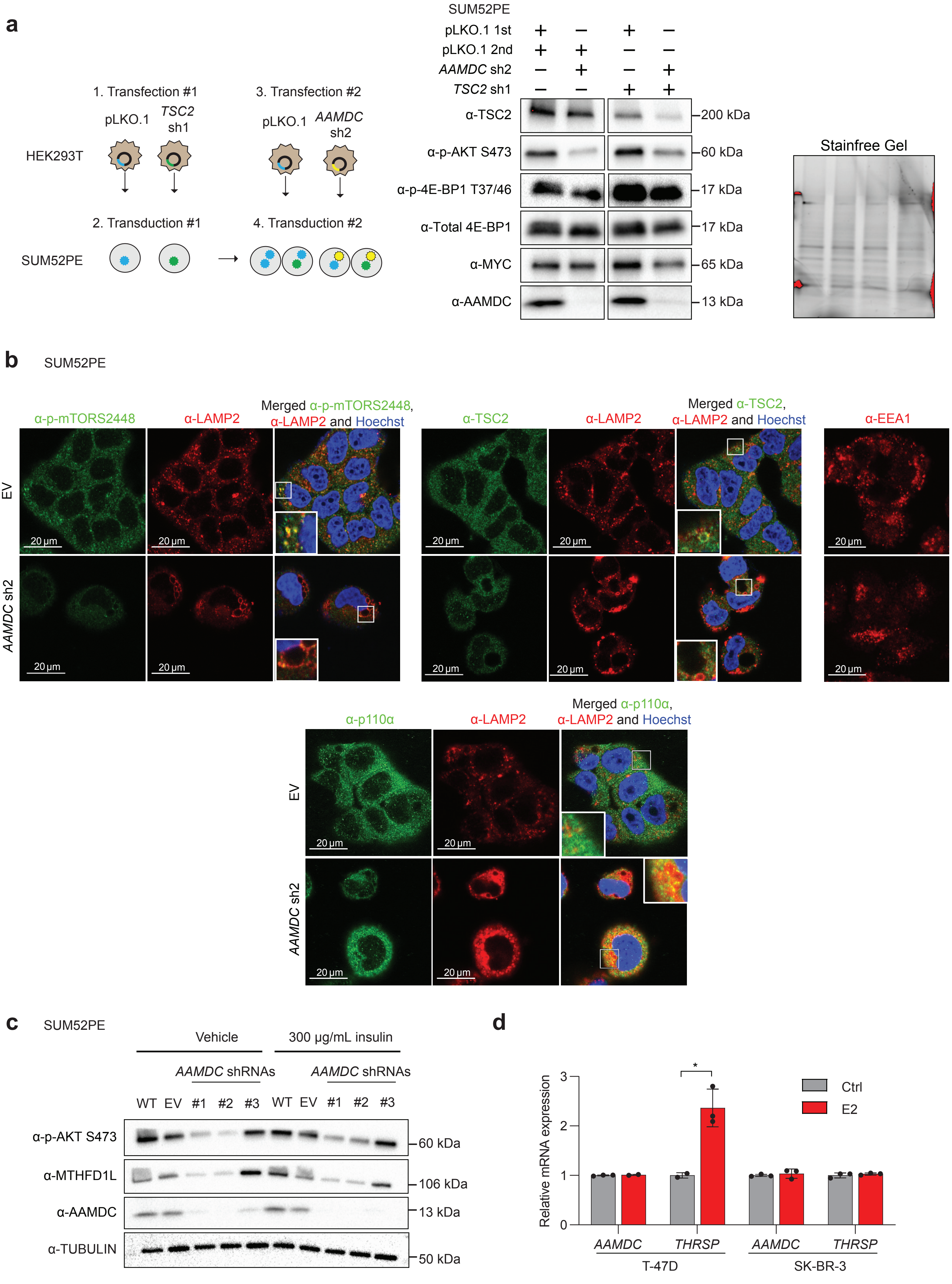

Supplementary Fig. 3. Relating to Fig. 4. and Fig. 6: Impact of AAMDC in lysosomal and vesicle trafficking.

a, Immunoblot (IB) of SUM52PE cells transduced with either pLKO.1 empty vector (EV) (1st) or with a *TSC2* shRNA 1 (sh1) and subsequently transduced with either an EV pLKO.1 (2nd) or *AAMDC* shRNA#2 (sh2). Stain-free images indicating equal total protein loading are shown. Source data are provided as a Source Data file.

b, Immunofluorescence images of SUM52PE cells transduced with *AAMDC* sh2 or EV stained with several antibodies for the detection of p-mTOR S2448 (green) and LAMP2 (red) (top left); TSC2 (green) and LAMP2 (red) (top middle); EEA1 (red) and Hoechst (blue) (top right); p110α catalytic subunit of PI3K (green), LAMP2 (red), and Hoechst (blue) (bottom).

c, Immunoblotting of SUM52PE cells transduced with *AAMDC* shRNAs or EV, insulin starved overnight and then treated with vehicle or 300 μg/mL insulin for 7 h. Source data are provided as a Source Data file.

d, Relative mRNA expression of *AAMDC* and *THRSP* in response to estrogen stimulation in the estrogen receptor positive (ER^+) T-47D and the estrogen receptor negative (ER^-) SK-BR-3 cell lines. Cells were treated with vehicle (Ctrl, grey) or 1 nM β-estradiol (E2, red). Data are presented as mean values +/- SD. Statistical significance is determined by a two-tailed unpaired *t*-test ($*p = 0.017$) for $n = 3$ biological replicates.

Supplementary Figure 4 (convergent genes)

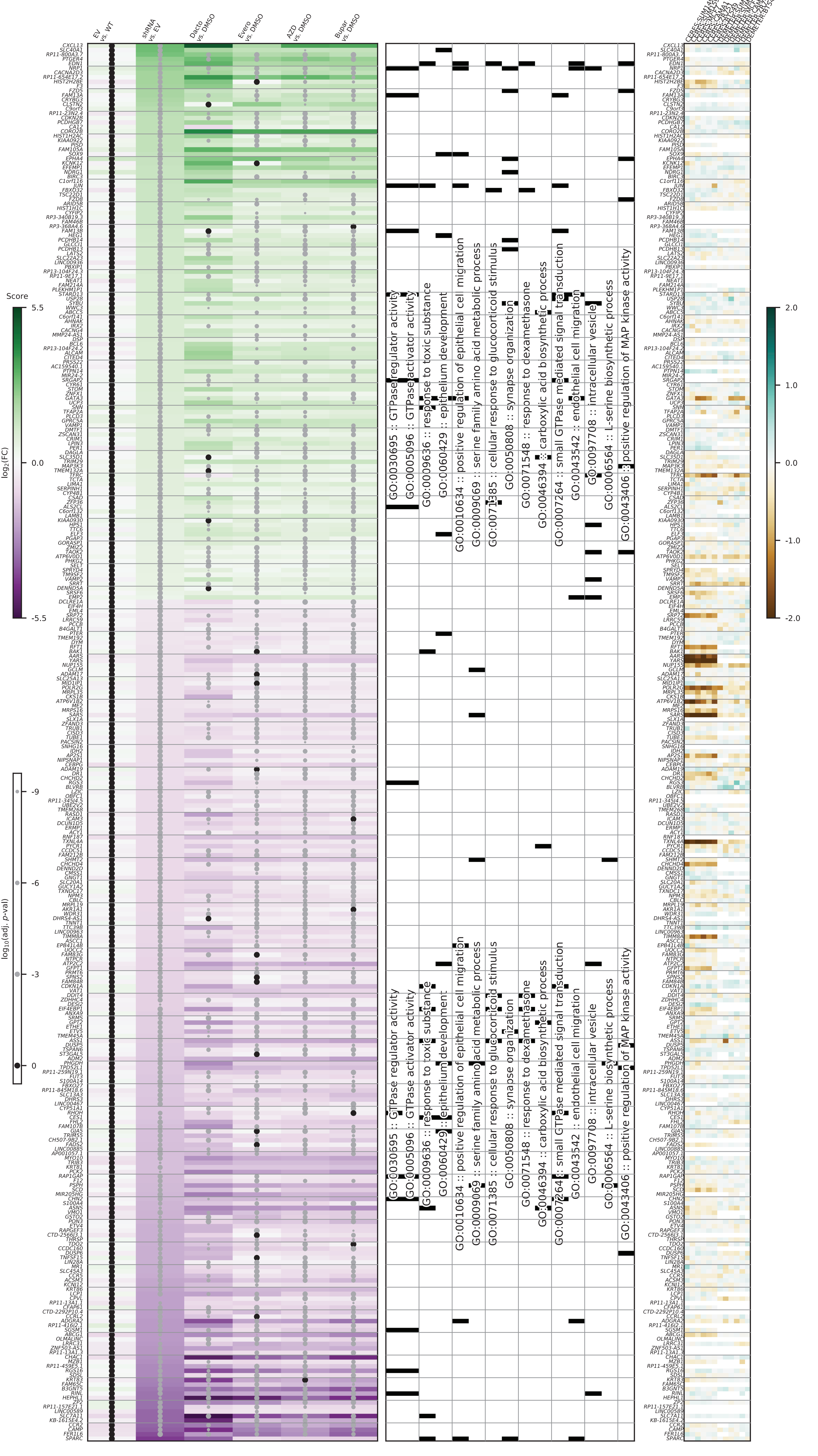

Supplementary Fig. 4. Relating to Fig. 5: Convergent genes for PI3K-AKT-mTOR inhibition and *AAMDC* shRNA.

Changes in transcript abundance (log_2 fold-change (log_2FC); color scale (left): downregulated (purple), upregulated (green) for the log_2FC) for specified genes with an overlaid estimate of the significance of the differential expression (adjusted *p*-value; key at left). Genes are selected based on significant (adjusted p-value < 0.01) differential expression in a consistent direction for at least 3 of the 4 drug treatment conditions, as well as the *AAMDC* shRNA treatment. Membership of the indicated genes within the most enriched Gene Ontology (GO) (black rectangle indicates membership; in the middle), with the GO categories ranked left to right by decreasing significance. Gene scores for essentiality are indicated as calculated by DepMap for CRISPRi (CERES score) or RNAi (DEMETER2 score) experiments (more negative scores indicate stronger repression of cell survival/growth, on the right).

Supplementary Figure 5 (divergent genes)

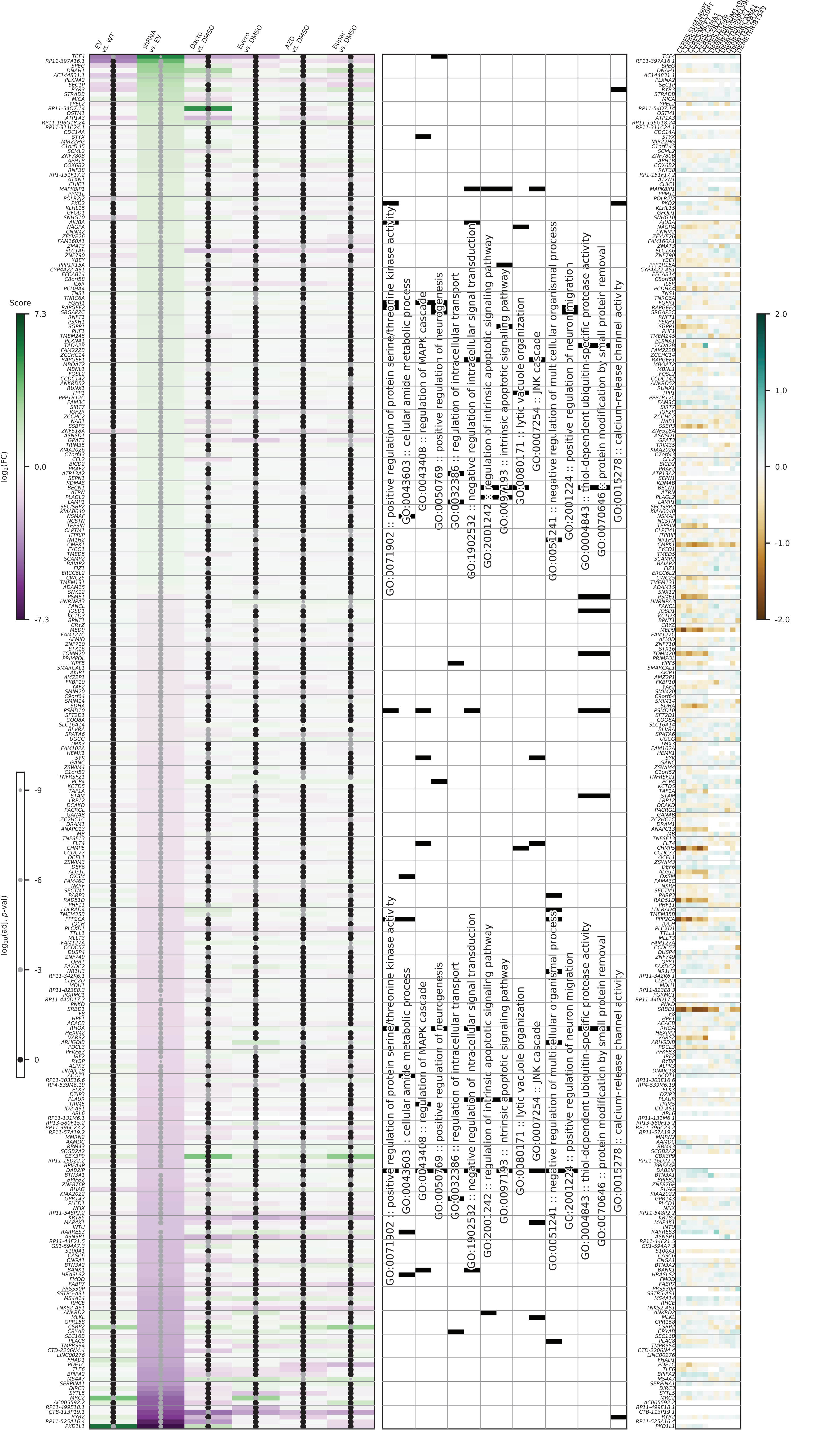

Supplementary Fig. 5. Relating to Fig. 5: Divergent genes for PI3K-AKT-mTOR inhibition and *AAMDC* shRNA.

Changes in transcript abundance ($\log_2$ fold-change ($\log_2$FC); color scale (left): downregulated (purple), upregulated (green) for the $\log_2$FC) for selected genes with an overlaid estimate of the significance of the differential expression (adjusted *p*-value; key at left). Genes are selected based on having both significant (adjusted p-value < 0.01) differential expression in the *AAMDC* shRNA treatment, and no significant differential expression (in the same direction) within any drug-treated sample. Membership of the indicated genes within the most enriched Gene Ontology (GO) (black rectangle indicates membership; in the middle), with the GO categories ranked left to right by decreasing significance. The gene scores are indicated for essentiality as calculated by DepMap for CRISPRi (CERES score) or RNAi (DEMETER2 score) experiments (more negative scores indicate stronger repression of cell survival/growth, on the right).

Supplementary Figure 6

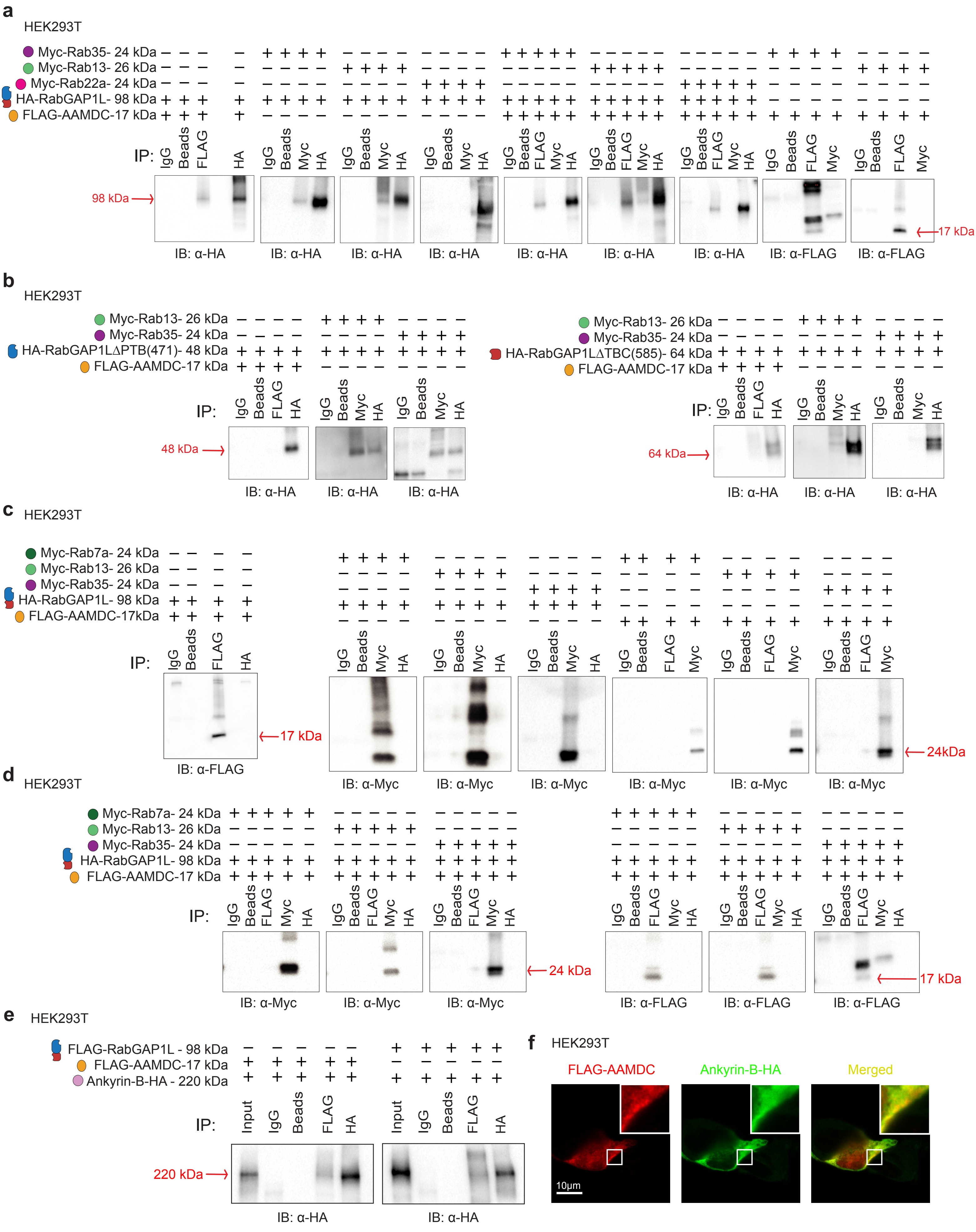

Supplementary Fig. 6. Relating to Fig. 8d-f: Interactions between AAMDC, RabGAP1L, and Rab proteins assessed by immunoprecipitation experiments.

a-d, Interaction between AAMDC and RabGAP1L, and selected Rab proteins (Rab22a, Rab7a, Rab13 and Rab35) by immunoprecipitation (IP) experiments in HEK293T cells transiently transfected with combinations of the tagged cDNAs: *HA-RabGAP1L*, *HA-RabGAP1LΔPTB*, *HA-RabGAP1LΔTBC*, *FLAG-AAMDC*, and *Myc-Rab13*, *Myc-Rab22a, Myc-Rab35,* and *Myc-Rab7a.* The IP products were immunoblotted with α-HA antibody to detect HA-RabGAP1L (98 kDa), α-FLAG to detect FLAG-AAMDC (17 kDa), or α-Myc to detect Myc-Rab (24 kDa). Deletion of the phosphotyrosine binding (PTB) domain is indicated by HA-RabGAP1LΔPTB(471) (48 kDa) and deletion of the Rab-binding Tre-2/Bub2/CdC16 (TBC) domain by HA-RabGAP1LΔTBC(585) (64 kDa). Immunoglobulin G (IgG)-conjugated beads and beads only (beads) were processed in parallel IPs as negative controls.

e, Interaction between Ankyrin-B and AAMDC as assessed by IP experiments in HEK293T cells transiently transfected with *Ankyrin-B-HA* and *FLAG-AAMDC*. The indicated pull-downs were immunoblotted with an α-HA antibody to detect Ankyrin-B-HA. Immunoprecipitations of cell extracts co-transfected with *Ankyrin-B-HA* and *FLAG-RabGAP1L* were included as positive controls.

f, Immunofluorescence images of HEK293T cells transiently co-transfected with *Ankyrin-B-HA* cDNA or *FLAG-AAMDC* cDNA. The α-FLAG staining for the detection of AAMDC (red) and α-HA staining for the detection of Ankyrin-B (green). The overlay of images is shown in the merged panel.

Supplementary Figure 7

a

HEK293T

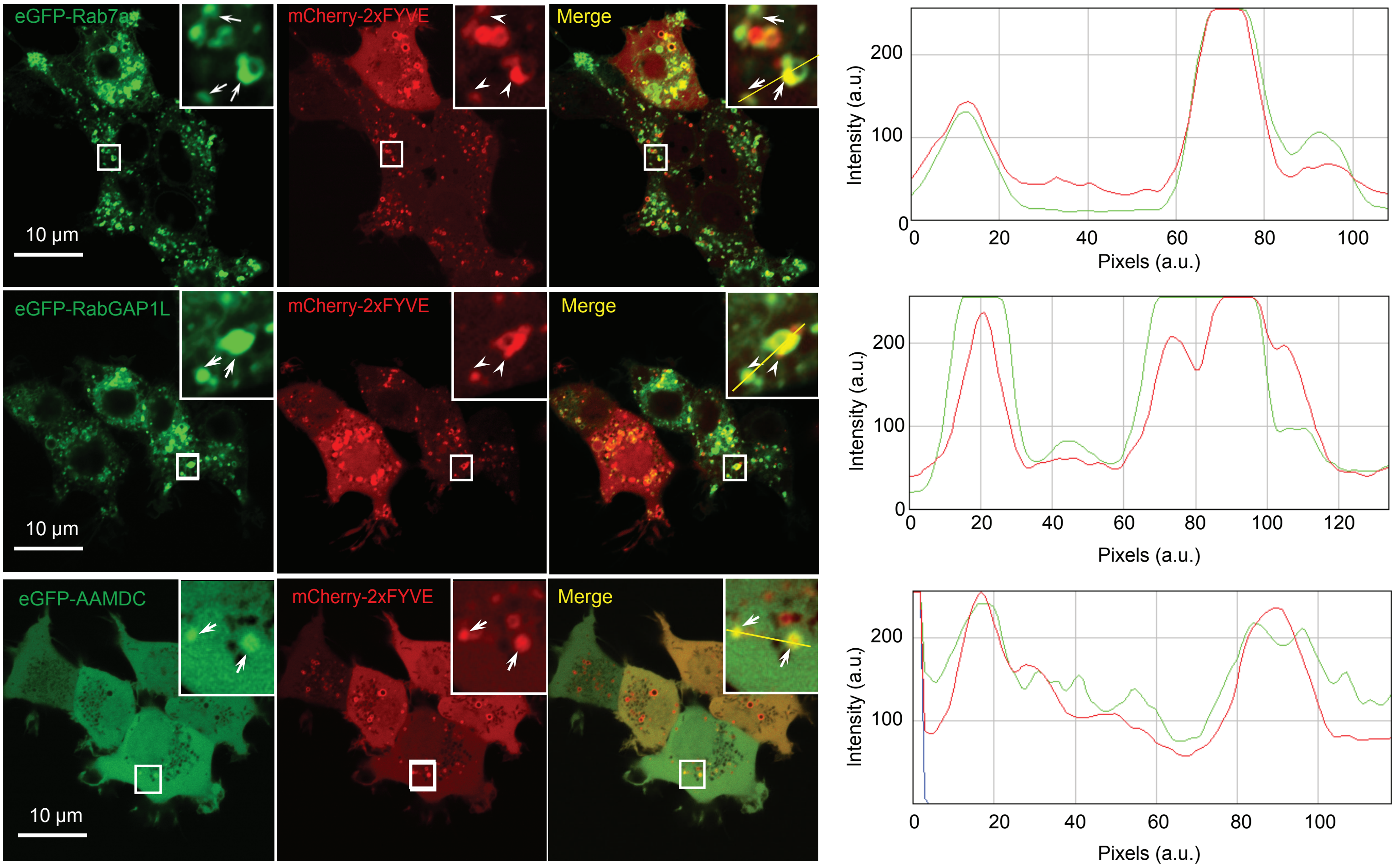

b

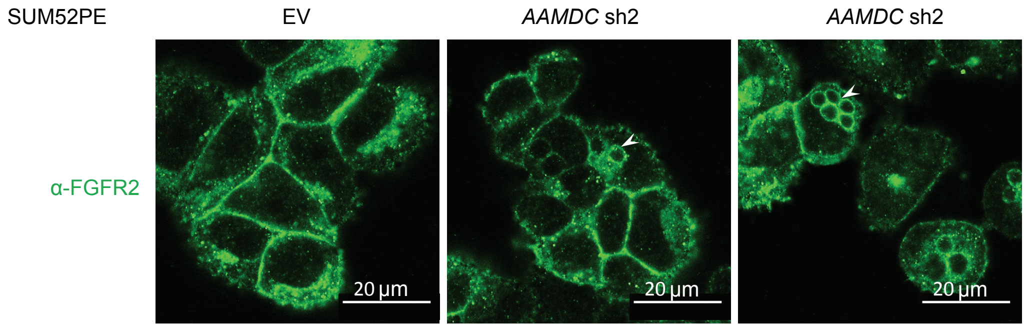

Supplementary Fig. 7. Relating to Fig. 8: AAMDC and RabGAP1L localize into PI3P positive vesicles.

a, Live-cell immunofluorescence of HEK293T cells transiently transfected with the phosphatidylinositol 3-phosphate (PI3P) marker, *mCherry-2xFYVE* (red) and either e*GFP-Rab7a, eGFP- RabGAP1L, or eGFP-AAMDC* (green). An intensity trace of the merged image section (yellow line) in the inset is shown (right panels); eGFP intensity (green line) and mCherry intensity (red line). a.u., arbitrary unit.

b, Immunofluorescence of SUM52PE cells transduced with empty vector (EV) or *AAMDC* shRNA 2 (sh2) showing the localization of the FGFR2 (green). Circular structures represent enlarged late endosomes or endolysosomes induced by the *AAMDC* knockdown (white arrows).

Supplementary Figure 8

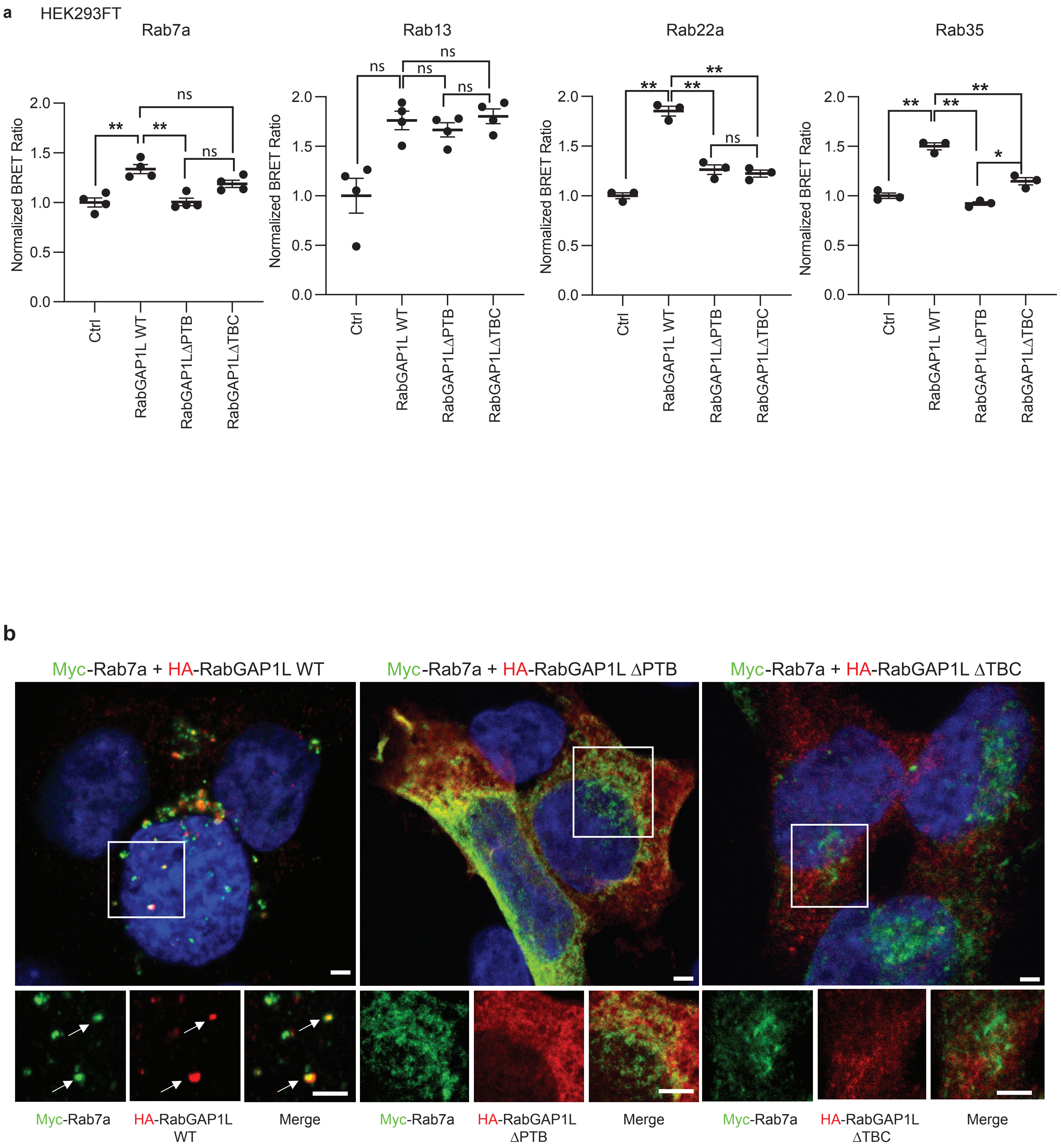

Supplementary Fig. 8. Relating to Fig. 8g: Localization of AAMDC, RabGAP1L, and selected Rabs in HEK293 cells.

a, Bioluminescence resonance energy transfer (BRET) signal release in HEK293FT cells transiently overexpressing *RLuc8-AAMDC* and either *Venus-Rab7a*, *-Rab13*, *-Rab22a*, or -*Rab35*. The Rab7a and Rab22a BRET results are shown in Fig. 8g. Cells were additionally co-transfected with either full-length *RABGAP1L* (*RabGAP1L WT*) or deletion mutants (*RabGAP1LΔPTB and RabGAP1LΔTBC*). Control cells (Ctrl) were co-transfected with *Venus-Rab* and *RLuc8-AAMDC* only. Individual values for $n = 4$ biological replicates (Rab7a and Rab13) and for $n = 3$ biological replicates (*Rab22a* and *Rab35*) are shown as mean +/- SEM and statistical significance determined by Brown-Forsythe and Welch ANOVA tests with Dunnett's T3 multiple comparison test *Rab7a*: $^{**}p = 0.0096$ for *Ctrl* vs. *RabGAP1L WT*, $^{**}p = 0.0069$ for *RabGAP1L WT* vs. *RabGAP1LΔPTB*, and not significant (ns) for *RabGAP1L WT* vs. *RabGAP1LΔTBC* and for *RabGAP1LΔPTB* vs. *RabGAP1LΔTBC*; Rab13: ns for all the conditions; Rab22a: $^{**}p = 0.0024$ for Ctrl vs. *RabGAP1L WT*, $^{**}p = 0.0045$ for *RabGAP1L WT* vs. *RabGAP1LΔPTB*, $^{**}p = 0.0020$ for *RabGAP1L WT* vs. *RabGAP1LΔTBC*, and ns for *RabGAP1LΔPTB* vs. *RabGAP1LΔTBC*; *Rab35*: $^{**}p = 0.0016$ for *Ctrl* vs. *RabGAP1L WT*, $^{**}p = 0.0026$ for *RabGAP1L WT* vs. *RabGAP1LΔPTB*, $^{**}p = 0.0099$ for *RabGAP1L WT* vs. *RabGAP1LΔTBC*, and $^{*}p = 0.0450$ for *RabGAP1LΔPTB* vs. *RabGAP1LΔTBC*.

b, Representative immunofluorescence images of HEK293T cells transiently co-transfected with the following constructs at a 1:1 molar ratio of plasmid DNA: either *HA-RabGAP1L WT* or deletion mutants (*RabGAP1LΔPTB* and *RabGAP1LΔTBC*) and *Myc-Rab7a*. α-HA staining for detection of HA-RabGAP1L (red); α-Myc staining for detection of Myc-Rab7a (green); overlayed images in the merged panel; Hoechst 33258 nuclear staining (blue). Scale bar = 2 μm.

Supplementary Figure 9

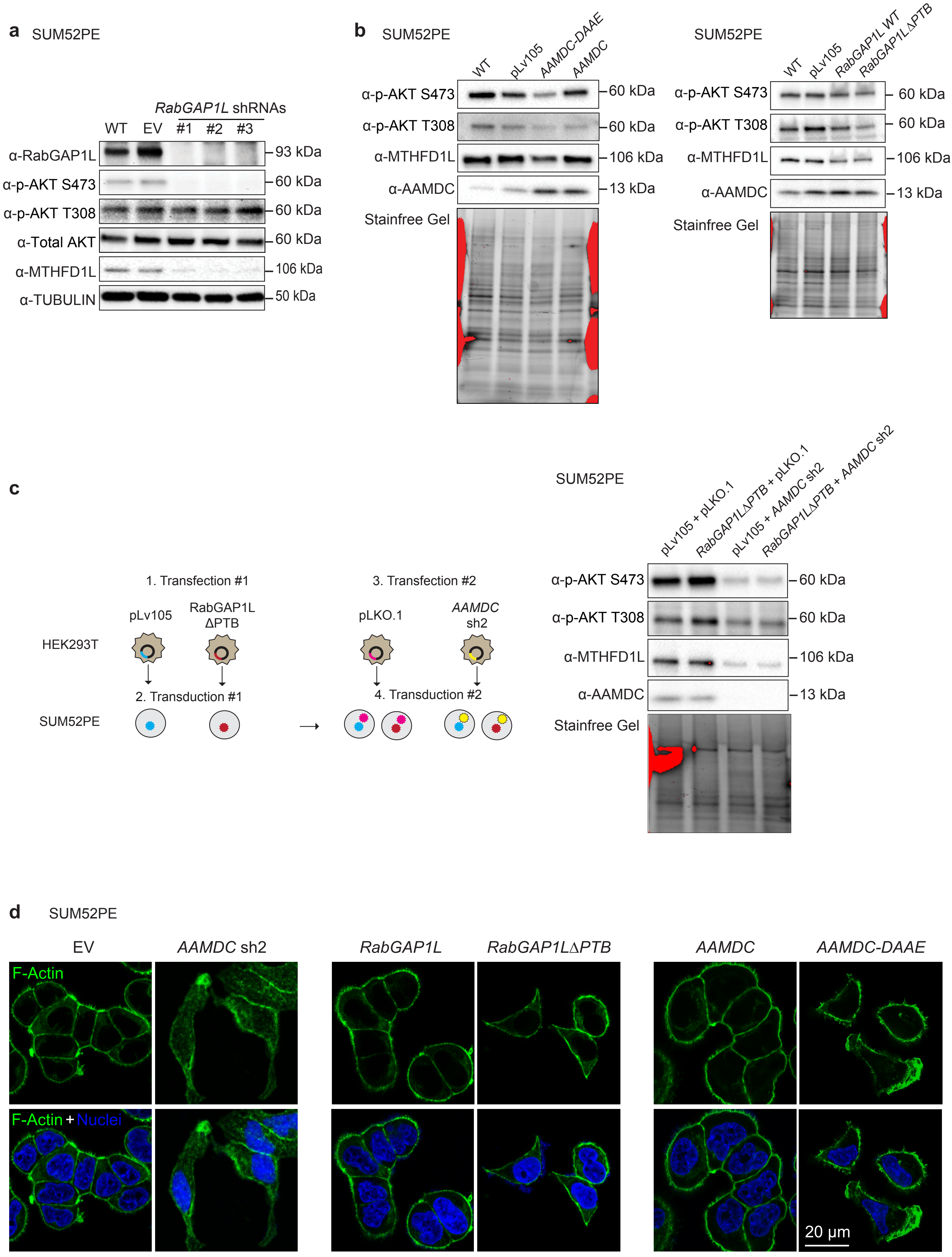

Supplementary Fig. 9. Relating to Fig. 8: RabGAP1L knockdown and RabGAP1L mutants downregulate PI3K-AKT-mTOR signaling.

a, Regulation of the PI3K-AKT-mTOR axis in SUM52PE cells transduced with *RabGAP1L* shRNAs as assessed by immunoblotting. EV, empty vector; WT, wild-type untransduced cells. Source data are provided as a Source Data file.

b, Immunoblots of SUM52PE cells lentivirally transduced with either pLv105 EV, *AAMDC* cDNA, or *AAMDC* cDNA harboring mutations in the predicted RabGAP1L binding motif (*AAMDC-DAAE*) (left). The same immunoblots of SUM52PE cells transduced with either pLv105 EV, full-length *RabGAP1L,* or the *RabGAP1L* mutant (*RabGAP1ΔPTB*) (right). Source data are provided as a Source Data file.

c, Immunoblots of SUM52PE cells lentivirally transduced with an empty vector pLv105 or *RabGAP1LΔPTB* (transfection #1). These cells were then transduced with either a second empty vector pLKO.1 or with *AAMDC* shRNA #2 (sh2) (transfection #2). Schematic representation of the double transduction protocol (left). Assessment of the regulation of the PI3K-AKT-mTOR axis by immunoblot (right). Source data are provided as a Source Data file.

d, Immunofluorescence staining of F-actin (phalloidin, green) in SUM52PE cells lentivirally transduced with an empty vector (EV) or *AAMDC* sh2, and with full-length *RabGAP1L*, *RabGAP1LΔPTB, AAMDC,* or *AAMDC-DAAE* cDNAs. Hoechst 33258 nuclear staining (blue) is shown.

Supplementary Figure 10

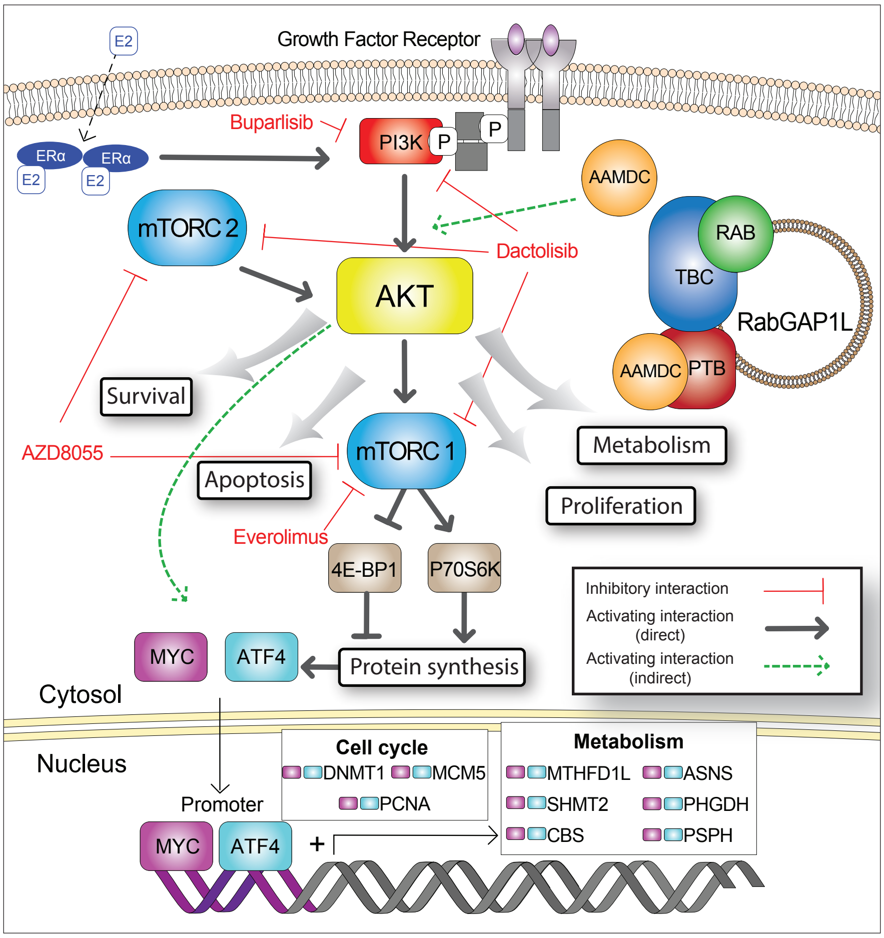

Supplementary Fig. 10. Schematic representation of the mechanism/model of action of oncogenic AAMDC.

The AAMDC oncoprotein activates the PI3K-AKT-mTOR signaling axis, thereby resulting in the upregulation of translation of MYC and ATF4, which in turn regulate transcription of cell-cycle, DNA replication, and metabolic genes. Bioinformatically-predicted MYC and ATF4 binding motifs in the promoter of the *AAMDC*-regulated genes are shown in pink and blue, respectively. The phosphotyrosine binding (PTB) domain and the Tre-2/Bub2/CdC16 (TBC) domain of RabGAP1L are indicated. ER, estrogen receptor; E2, estrogen.

Supplementary Table 1. Relating to Fig. 1c-d: IHC scoring of the breast cancer TMA comprising 60 high-risk breast cancers and benign tissue.

	AAMDC Overall H-score >1.5 cut-off					AAMDC Cytoplasmic H-score >1.0 cut-off					AAMDC Nuclear H-score ≥0.5 cut-off				
**AAMDC IHC Status**	**Negative**		**Positive**		**Sig.***	**Negative**		**Positive**		**Sig.***	**Negative**		**Positive**		**Sig.***
**Median Age (range)**	46 (28-70)		51.5 (30-74)			48 (28-70)		51 (30-74)			50 (28-70)		49.5 (30-74)		
	**N**	**(%)**	**N**	**(%)**		**N**	**(%)**	**N**	**(%)**		**N**	**(%)**	**N**	**(%)**	
**All Patients**	26	(43.3)	34	(56.7)	-	30	(50)	30	(50)		29	(53.3)	31	(46.7)	
**Size**															
0-5cm	22	(84.6)	23	(67.6)	0.06	25	(83.3)	20	66.7	0.07	25	(86.2)	20	(64.5)	0.03
5.0cm+	4	(15.4)	11	(32.3)		5	16.7	10	33.3		4	(13.8)	11	(35.5)	
**Grade**															
1-2	21	(80.8)	30	(88.2)	0.21	25	(83.3)	26	(87.1)	0.36	23	(79.3)	28	(90.3)	0.12
3	5	(19.2)	4	(11.8)		5	(16.7)	4	(13.3)		6	(20.7)	3	(9.7)	
**LN Status**															
NO	18	(69.2)	20	(58.8)	0.2	19	(63.3)	19	(63.3)	1	22	(75.9)	16	(51.6)	0.03
N1-2	8	(30.8)	14	(41.2)		11	(36.7)	11	(36.7)		7	(24.1)	15	(48.4)	
**ER Status**															
Negative	22	(84.6)	18	(52.9)	0.005	25	(83.3)	15	(50)	0.003	24	(82.8)	16	(51.6)	0.005
Positive	4	(15.4)	16	(47.1)		5	(16.7)	15	(50)		5	(17.2)	15	(48.4)	
**PR Status**															
Negative	20	(76.9)	24	(70.6)	0.29	25	(83.3)	19	(63.3)	0.04	21	(72.4)	23	(74.2)	0.44
Positive	6	(23.1)	10	(29.4)		5	(16.7)	11	(36.7)		8	(27.6)	8	(25.8)	
**HER2 status**															
0-1	20	(76.9)	25	(73.5)	0.38	21	(70.0)	24	(80.0)	0.19	21	(72.4)	24	(77.4)	0.33
2-3	6	(23.1)	9	(26.5)		9	(30.0)	6	(20.0)		8	(27.6)	7	(22.6)	
**Intrinsic Sub-type**															
Luminal A	5	(19.2)	8	(23.5)		5	(16.7)	8	(26.7)		5	(17.2)	8	(25.8)	
Luminal B	3	(15.4)	8	(23.5)		3	(10.0)	8	(26.7)		4	(13.8)	7	(22.6)	
HER2-enriched	4	(11.5)	9	(26.5)	-	7	(23.3)	6	(20.0)		7	(24.1)	6	(19.4)	
Basal	14	(53.8	9	(26.5)		15	(50.0)	8	(26.7)		13	(44.8)	10	(32.3)	

*Chi-square

Supplementary Table 1. Relating to Fig. 1c-d: IHC scoring of the breast cancer tissue microarray (TMA) comprising 60 high-risk breast cancers and benign tissue.

Correlations between AAMDC localization and various tumor characteristics, including hormone receptor positivity, the tumor size, and the grade, as determined by immunohistochemistry (IHC) scoring (the H-score cut-off is indicated). Significance values are determined using the chi-square test with a one-tailed *p*-value on the pairs indicated.

Supplementary Table 2. List of all primer names, sequences, and the applications described in METHODS.

Name	Sequence	Application
MTHFD1L Forward	5'-GTTTAGGGGCGATTTTGTGACCAC-3'	ChIP-qPCR
MTHFD1L Reverse	5'-TGATTGGTTCCAGGGCCCCTC-3'	ChIP-qPCR
ASNS Forward	5'-AGTCCTGCTCCGCCC-3'	ChIP-qPCR
ASNS Reverse	5'-GCACGCGAGGAGGATGC-3'	ChIP-qPCR
*hActB*_ex6_Forward	5'-GATGAGATTGGCATGGCTTT-3'	ChIP-qPCR
*hActB*_ex6_Reverse	5'-CACCTTCACCGTTCCAGTTT-3'	ChIP-qPCR
RabGAP1LΔTBC(585) Forward	5'-CAGCCTCCGGACTCTAGC-3'	Molecular cloning
RabGAP1LΔTBC(585) Reverse	5'-CTCCTCCTCCTCCTCCTCCTCGCGGCCGCACTCGAGCTACACCAGAGTAGACAGCCCTTT-3'	Molecular cloning
RabGAP1LΔPTB(471) Forward	5'-GAGGAGGAGGAG GAGGAGGTTTAAACCGATTTTGGTATTTCAGCAG-3'	Molecular cloning
RabGAP1LΔPTB(471) Reverse	5'-GCTTATAATACGACTCACTATAGGG-3'	Molecular cloning
