# Supplementary material for: The oncogene AAMDC links PI3K-AKT-mTOR signaling with metabolic reprograming in estrogen receptor-positive breast cancer
Source: Nat Commun. 2021 Mar 26;12:1920. doi: 10.1038/s41467-021-22101-7 (PMC7998036; doi:10.1038/s41467-021-22101-7)
Supplement: Supplementary file 2 — Description of Additional Supplementary Files [file 41467_2021_22101_MOESM2_ESM.pdf]

Description of Additional Supplementary Files

Supplementary Data 1. Relating to Fig. 1e: Fluorescence in situ hybridization (FISH) assessment of adipogenesis associated Mth938 domain containing (AAMDC) and histological characteristics of tumors from a cohort of 119 breast cancer patients. a, Clinically available characteristics of the tumors analyzed by fluorescence in situ hybridization FISH, including the tumor grade and the presence of lymph node and or/distal metastasis. b, Cases with no amplification of adipogenesis associated Mth938 domain containing (AAMDC). c, Cases with amplification of AAMDC. d, Cases with polysomy of chromosome 11. NR, no result or data available; IDC, invasive ductal carcinoma; ILC, invasive lobular carcinoma; Poly, polysomy of chromosome 11; Amp, amplification; TNM, Tumor, Nodes, and Metastases.

Supplementary Data 2. Relating to Fig. 3a-c: Genome-wide gene expression analyses of SUM52PE cells transduced with adipogenesis associated Mth938 domain containing (AAMDC) shRNA #2 (sh2) vs. empty vector control. Relative change in gene transcript abundance (log2 fold-change (log2FC) mRNA) determined by RNA sequencing of SUM52PE cells transduced with adipogenesis associated Mth938 domain containing (AAMDC) shRNA #2 (sh2). a, Annotated genes that are significantly downregulated in the AAMDC knockdown (KD) compared to the empty vector (EV). Differential gene expression analysis was performed using Cuffdiff (v2.2.1), with significant changes in gene expression determined using a q-value < 0.05 in three biological replicates. Raw and normalized RNA-Seq data are available in GSE92893. b, Annotated genes that are significantly upregulated in the AAMDC KD compared to EV. Differential gene expression analysis was performed as in (a). c, Significantly and differentially downregulated genes in the AAMDC sh2 RNA sequencing identified by Gene Ontology (GO) enrichment analysis as lipid metabolism related genes. These genes are significantly enriched in the RNAseq dataset (1.66-fold, p = 9.47 x 10-7).

Supplementary Data 3. Relating to Fig. 3f: Liquid chromatography-mass spectrometry (LC-MS) metabolomic analyses of SUM52PE cells transduced with adipogenesis associated Mth938 domain containing (AAMDC) shRNA #2 (sh2) vs. wild-type (WT) or empty vector (pLKO.1). a, Key to terms shown in (b). b, Significantly and differentially synthesized metabolites measured in triplicate samples of SUM52PE cells untransduced wild-type (WT) or lentivirally transduced with either empty vector (pLKO.1) or AAMDC shRNA #2 (sh2). Significance was defined as (p < 0.05, two-tailed unpaired t-test) and regulated metabolites determined by p < 0.05 and | log2FC | > 1.0.

Supplementary Data 4. Relating to Fig. 5: Gene Set Enrichment Analysis of SUM52PE adipogenesis associated Mth938 domain containing (AAMDC) shRNA #2 (sh2) and PI3K/mTOR pathway inhibitor-treated cells. a, List of significant pathways (p < 0.05) that are enriched with differentially regulated genes by adipogenesis associated Mth938 domain containing (AAMDC) shRNA #2 (sh2) or by specific inhibitors (dactolisib, everolimus, AZD8055, and buparlisib). Hallmark pathways (MSigDB, GSEA) that are significantly enriched (NOM p-val < 0.05) in each comparison are ranked by normalized enrichment score (NES). GSEA statistics values (NES, FDR (false discovery rate), FWER (family-wise error rate), and NOM p-val (nominal pvalues)) are determined as previously described (https://pubmed.ncbi.nlm.nih.gov/16199517/). b, List of convergent genes that are regulated similarly for the AAMDC sh2 and the indicated pharmacological inhibitors. The data expressed as log2 foldchange (log2FC) across the AAMDC sh2 and the drug-treated samples, and according to the HGNC (Hugo Gene Nomenclature Committee). c, List of divergent genes that are regulated in an opposite manner by the AAMDC sh2 and the indicated pharmacological inhibitors. The data expressed as log2FC

across AAMDC sh2 and drug-treated samples and according to HGNC, Hugo Gene Nomenclature Committee.

Supplementary Data 5. Relating to Fig. 8: Yeast Two-Hybrid (Y2H) data. a, Summary of the prey genes identified by Y2H as interacting with adipogenesis associated Mth938 domain containing (AAMDC). The Rab GTPase activating protein 1 like (RabGAP1L) was identified as a very high confidence prey in two independent screens using two bait constructs: N-LexA-AAMDC-C fusion and N-GAL4-AAMDC-C fusion. The predicted biological scores (PBS) are: A, Very high confidence in interaction; B, High confidence in the interaction; C, Good confidence in the interaction; and D, Moderate confidence in the interaction. b, Sequences of the prey fragments isolated from a library of human breast luminal cells identified by Y2H screenings as interacting with AAMDC bait constructs (N-LexA-AAMDC-C and N-Gal4-AAMDC-C). Each fragment is assigned to a GenBank gene identification based on the fragment sequence and a PBS score as a measure of the confidence of the interaction.

Supplementary Data 6. Key Resources Table. A complete list of resources used for undertaking this study.
