# Supplementary material for: The oncogene AAMDC links PI3K-AKT-mTOR signaling with metabolic reprograming in estrogen receptor-positive breast cancer
Source: Nat Commun. 2021 Mar 26;12:1920. doi: 10.1038/s41467-021-22101-7 (PMC7998036; doi:10.1038/s41467-021-22101-7)
Supplement: Supplementary file 9 — Reporting summary [file 41467_2021_22101_MOESM9_ESM.pdf]

nature research

Corresponding author(s): Pilar Blancafort

Last updated by author(s): Feb 11, 2021

Reporting Summary

Nature Research wishes to improve the reproducibility of the work that we publish. This form provides structure for consistency and transparency in reporting. For further information on Nature Research policies, see our Editorial Policies and the Editorial Policy Checklist.

Statistics

For all statistical analyses, confirm that the following items are present in the figure legend, table legend, main text, or Methods section.

n/a	Confirmed	
☐	☒	The exact sample size (n) for each experimental group/condition, given as a discrete number and unit of measurement
☐	☒	A statement on whether measurements were taken from distinct samples or whether the same sample was measured repeatedly
☐	☒	The statistical test(s) used AND whether they are one- or two-sided<br>*Only common tests should be described solely by name; describe more complex techniques in the Methods section.*
☐	☒	A description of all covariates tested
☒	☐	A description of any assumptions or corrections, such as tests of normality and adjustment for multiple comparisons
☐	☒	A full description of the statistical parameters including central tendency (e.g. means) or other basic estimates (e.g. regression coefficient) AND variation (e.g. standard deviation) or associated estimates of uncertainty (e.g. confidence intervals)
☐	☒	For null hypothesis testing, the test statistic (e.g. F, t, r) with confidence intervals, effect sizes, degrees of freedom and P value noted<br>*Give P values as exact values whenever suitable.*
☒	☐	For Bayesian analysis, information on the choice of priors and Markov chain Monte Carlo settings
☒	☐	For hierarchical and complex designs, identification of the appropriate level for tests and full reporting of outcomes
☒	☐	Estimates of effect sizes (e.g. Cohen's d, Pearson's r), indicating how they were calculated

Our web collection on statistics for biologists contains articles on many of the points above.

Software and code

Policy information about availability of computer code

Data collection	No Software was used for Data collection

Data analysis	Image processing and quantification were performed using NIS-Elements AR (Version 4.13) (Nikon Corporation, Tokyo, Japan) and ImageJ (Rasband, W.S., ImageJ, U. S. National Institutes of Health, Bethesda, Maryland, USA, https://imagej.nih.gov/ij/, 1997-2018). DAVID (v6.8) was used for gene ontology functional annotation analysis. Structure analysis was performed using PyMOL (v2.2) (Schrödinger) and modelling was performed using Phyre2 (v2.0) (Structural Bioinformatics Group, Imperial College, London). Combination index determination was performed using the median dose effect method proposed by Chou and Talalay with the CompuSyn software (ComboSyn). TMA visualization was performed using Aperio ImageScope Pathology Slide Viewing Software (v12.3.3) (Leica Biosystem, Nussloch, GmbH). Network analysis and KEGG pathways map of differentially regulated targets were performed by STRING database (v11) (http://string-db.org). RNA-sequencing data has been deposited in the Gene Expression Omnibus public database under accession numbers GSE92893 and GSE123740. All repository data has been made publicly available (https://github.com/jcursons/Golden_2021_NatComm). Analysis of somatic alterations for the AAMDC gene in Fig. 1a was performed using cancer genomic data sets and tools from cBioPortal (https://www.cbioportal.org/). Survival analyses of cancer patients with high and low expression levels of AAMDC (Fig. 1b) is performed in the PPISURV portal http://www.bioprofiling.de/GEO/PPISURV/ppisurvD.html). The survival analyses of breast, ovarian and lung cancer patients were performed by using the GSE11121, GSE13876, and GSE19188 GEO datasets, respectively. Survival of Luminal B patients treated with tamoxifen with high and low expression of AAMDC was compared using Kaplan Meier Plotter server (https://kmplot.com/analysis/) and the GEO datasets: GSE12093, GSE16391, GSE17705, GSE19615, GSE26971, GSE2990, GSE3494, GSE37946, GSE45255, GSE6532, and GSE9195. GraphPad Prism (v8.4) was used for graphing and statistical analysis. For RNA-seq data shown in Fig. 3, sequenced reads were aligned to human (hg19) genome using TopHat (v2.0.14) and expression at the gene level (FPKM values) was estimated and normalized by Cufflinks (v2.2.1), Cuffmerge (v1.0.0) and Cuffnorm (v2.2.1). Differential gene expression analysis was performed using Cuffdiff (v2.2.1), with significant changes in gene expression determined using a q-value <0.05 in three biological replicates. For RNA-seq data shown in Fig. 5, sequenced reads underwent pseudo-alignment against the GRCh38 (Ensembl) reference genome and quantification using Salmon (v0.8.2). Data were imported into R (v3.5) using the Bioconductor package tximport (v1.12.3) and collapsed to the gene-level for differential expression analysis using DESeq2 (v1.24.0). Results were visualized with python (v3.6) using the matplotlib (v3.1.1), scipy (v1.3.0), numpy (v1.16.4+mkl), and pandas (v0.24.2) packages.

For manuscripts utilizing custom algorithms or software that are central to the research but not yet described in published literature, software must be made available to editors and reviewers. We strongly encourage code deposition in a community repository (e.g. GitHub). See the Nature Research guidelines for submitting code & software for further information.

Data

Policy information about availability of data

All manuscripts must include a data availability statement. This statement should provide the following information, where applicable:

- Accession codes, unique identifiers, or web links for publicly available datasets
- A list of figures that have associated raw data
- A description of any restrictions on data availability

RNA-sequencing data has been deposited in the Gene Expression Omnibus public database under accession numbers GSE92893 (https://www.ncbi.nlm.nih.gov/geo/query/acc.cgi?acc=GSE92893) and GSE123740 (https://www.ncbi.nlm.nih.gov/geo/query/acc.cgi?acc=GSE123740). Protein Data Bank (PDB) structures shown in Fig. 8 are available under the accession codes IHN (http://doi.org/10.2210/pdb1IHN/pdb) and 2AB1 (http://doi.org/10.2210/pdb2AB1/pdb). Metabolomics data is available in Supplementary Data 3. Yeast Two-Hybrid data is available in Supplementary Data 5. Code used to analyze 'DepMap' project data and to produce visualisations of RNA-seq data comparing specific drugs against shRNA-mediated AAMDC knockdown, are made publicly available on GitHub (https://github.com/jcursons/Golden_2021_NatComm).

Field-specific reporting

Please select the one below that is the best fit for your research. If you are not sure, read the appropriate sections before making your selection.

☒ Life sciences ☐ Behavioural & social sciences ☐ Ecological, evolutionary & environmental sciences

For a reference copy of the document with all sections, see nature.com/documents/nr-reporting-summary-flat.pdf

Life sciences study design

All studies must disclose on these points even when the disclosure is negative.

Sample size	For animal studies 8 mice/group was used to determine statistical significance. This is calculated based on the sample size power calculator where the type 1 (alpha) and type 2 (beta) probability are set to 0.05 and 0.2 accordingly.
Data exclusions	Data were not excluded except in cases of technical error.
Replication	At least three biological replicates were performed with successful outcomes.
Randomization	The phenotype of the engineered cell lines was visually obvious. Only the mouse xenograft study was randomized. Mice were randomly assigned to the specific groups indicated in the manuscript.
Blinding	Blinding was not performed for these studies since it required the generation of genetically engineered cell lines. Validation of these genetically engineered cell lines was necessary in order to confirm the downregulation of the gene before injecting in mice in accordance to the Animal Ethics approval. Also, the phenotype of the engineered cell lines was visually obvious.

Reporting for specific materials, systems and methods

We require information from authors about some types of materials, experimental systems and methods used in many studies. Here, indicate whether each material, system or method listed is relevant to your study. If you are not sure if a list item applies to your research, read the appropriate section before selecting a response.

Materials & experimental systems

n/a	Involved in the study
☐	☒ Antibodies
☐	☒ Eukaryotic cell lines
☒	☐ Palaeontology and archaeology
☐	☒ Animals and other organisms
☐	☒ Human research participants
☒	☐ Clinical data
☒	☐ Dual use research of concern

Methods

n/a	Involved in the study
☒	☐ ChIP-seq
☒	☐ Flow cytometry
☒	☐ MRI-based neuroimaging

Antibodies

Antibodies used

Mouse monoclonal anti-AAMDC Abcam Cat#ab180533, Rabbit polyclonal anti-AAMDC (PTD015, T-15) Santa Cruz Biotechnology Cat#sc-138556, Rabbit polyclonal anti-RabGAP1L Proteintech Cat#13894-1-AP, Rabbit monoclonal anti-MTHFD1L (D708E) Cell Signaling Technology Cat#14998, Rabbit polyclonal anti-FLAG™ Sigma-Aldrich Cat#F7425, Rabbit monoclonal anti-HA (C29F4) Cell Signaling Technology Cat#3724, Rabbit monoclonal anti-Myc Tag (71D10) Cell Signaling Technology Cat#2278, Mouse monoclonal anti-Myc-Tag (9B11) (Magnetic Bead Conjugate) Cell Signaling Technology Cat#5698, Mouse monoclonal anti-DYKDDDDK Tag (9A3) Cell Signaling Technology Cat#8146, Rat monoclonal anti-HA Tag Antibody (16.43) Novus Biologicals Cat#NBP2-50416, Mouse monoclonal anti-FLAG® M2 Magnetic Beads Sigma-Aldrich Cat#M8823, Mouse monoclonal anti-HA.11 Epitope Tag (16B12) BioLegend Cat#MMS-101P, Normal mouse anti-IgG Santa Cruz Biotechnology Cat#sc-2025, Mouse monoclonal anti-Ki-67 Cell Signaling Technology Cat#9449, Rabbit polyclonal anti-Total AKT Cell Signaling Technology Cat#9272, Rabbit monoclonal anti-Phospho-AKT (Ser473) Cell Signaling Technology Cat#4060, Rabbit monoclonal anti-Phospho-AKT (Thr308) Cell Signaling Technology Cat#13038, Rabbit monoclonal anti-Phospho-mTOR (Ser2448) Cell Signaling Technology Cat#5536, Rabbit monoclonal anti-Phospho-PDK1 (Ser241) Cell Signaling Technology Cat#3438, Rabbit monoclonal anti-Phospho-TSC2 (T1462) Cell Signaling Technology Cat#3617, Rabbit polyclonal anti-FOXO 1/3 (Thr24/Thr32) Cell Signaling Technology Cat#9464, Rabbit polyclonal anti-p70 S6 Kinase Antibody Cell Signaling Technology Cat#9202, Rabbit monoclonal anti-Phospho-p70 S6 Kinase (Thr389) Antibody Cell Signaling Technology Cat#9234, Rabbit monoclonal anti-Phospho-4E-BP1 (Thr37/46) Cell Signaling Technology Cat#2855, Rabbit monoclonal anti-Total 4E-BP1 (53H11) Cell Signaling Technology Cat#9644, Rabbit polyclonal anti-c-Myc Cell Signaling Technology Cat#9402, Rabbit monoclonal anti-ATF-4 Cell Signaling Technology Cat#11815, Mouse monoclonal anti-α-Tubulin Sigma-Aldrich Cat#T5168, Peroxidase-conjugated Goat anti-Rabbit IgG (H+L) Jackson ImmunoResearch Laboratories Cat#111-035-144, Peroxidase-conjugated Goat anti-Mouse IgG (H+L) Jackson ImmunoResearch Laboratories Cat#115-035-003, Goat anti-mouse Alexa Fluor 488-conjugated antibody Thermo Fisher Scientific Cat#A11001, Goat anti-rabbit Alexa Fluor 594-conjugated antibody Thermo Fisher Scientific Cat#A11012, Goat anti-mouse Alexa Fluor 594-conjugated antibody Thermo Fisher Scientific Cat#A11032, Goat anti-rabbit Alexa Fluor 488-conjugated antibody Thermo Fisher Scientific Cat#A11008, Goat anti-rat Alexa Fluor 647-conjugated antibody Thermo Fisher Scientific Cat#A21247, Mouse monoclonal anti-Ankyrin B Santa Cruz Biotechnology Cat#sc-12718, Rabbit monoclonal anti-Tuberin/TSC2 Cell Signaling Technology Cat#4308, Mouse monoclonal anti-LAMP-2 Santa Cruz Biotechnology Cat#sc-18822, Rabbit monoclonal anti-PI3 Kinase p110α Cell Signaling Technology Cat#4249, Rabbit polyclonal anti-EEA1 Cell Signaling Technology Cat#2411, Rabbit monoclonal anti-FGF Receptor 2 Cell Signaling Technology Cat#11835, Rabbit polyclonal anti-Cleaved Caspase-3 (Asp175) Cell Signaling Technology Cat#9661, Phalloidin stain Alexa Fluor 488 Invitrogen Cat#A12379.

Validation

All antibodies were validated for their associated applications (western blot, immunoprecipitation, ChIP or IF) by the manufacturing companies that they were purchased from, except Jackson ImmunoResearch and Santa Cruz Biotechnologies which are approved for use for the corresponding applications and validated by literature citations available on the website. Positive and negative controls with known levels of the target gene expression were used to validate the specificity and selectivity for the endogenous levels of the target gene expression. We then employed knockdown strategies to decrease the abundance of the target gene to confirm that the primary antibody is specific.

Eukaryotic cell lines

Policy information about cell lines

Cell line source(s)

SUM44PE Asterand Bioscience Cat#CS-04 r03, SUM52PE Asterand Bioscience Cat#CS-05 r03, T-47D ATCC Cat#HTB-133, BT-474 ATCC Cat#HTB-20, MCF-7 ATCC Cat#HTB-22, ZR-75-1 ATCC Cat#CRL-1500, MDA-MB-231 ATCC Cat#HTB-26, SUM159 Asterand Bioscience Cat#CS-08 r03, BT-549 ATCC Cat#HTB-122, SK-BR-3 ATCC Cat#HTB-30, HMEC ATCC Cat#PCS-600-010, hTERT-HME1 [ME16C] ATCC Cat#CRL4010, MCF-12A ATCC Cat#CRL-10782, HEK293T ATCC Cat#CRL-1573™, 293FT Thermo Fisher Scientific Cat#R70007, MDA-MB-134 ATCC Cat#HTB-23, HDFa ATCC Cat#PCS-201-012, 3T3-L1 ATCC Cat#CL-173.

Authentication

Cell lines were purchased from ATCC and Asterand Biosciences and are all authenticated by STR profiling using Promega PowerPlex(R) 1.2 system and Applied Biosystems Genotyper 2.0 software for analysis of the amplicons.

Mycoplasma contamination

Cell lines were purchased from ATCC and Asterand Biosciences and are confirmed negative of mycoplasma in our laboratory before and after performing biological assays and re-tested regularly.

Commonly misidentified lines (See ICLAC register)

None of the cell lines used in this study are included in the ICLAC register.

Animals and other organisms

Policy information about studies involving animals; ARRIVE guidelines recommended for reporting animal research

Laboratory animals	5 week old, female, BALB/cJ Foxn1nu/Arc (Nude mice), weighing 11-17g.
Wild animals	Study did not involve wild animals.
Field-collected samples	Study did not involve field-collected samples.
Ethics oversight	The Animal Ethics Committee of the University of Western Australia, Australia, protocol RA/3/100/1159 and RA/3/100/1687.

Note that full information on the approval of the study protocol must also be provided in the manuscript.

Human research participants

Policy information about studies involving human research participants

Population characteristics	Immunohistochemistry of estrogen receptors as well as AAMDC gene amplification studies by FISH were performed on a total of 119 breast cancer samples obtained after surgery of the primary tumor. The age of onset is as follow: 31 to 91, average 60.8; in a cohort without amplification: 31 to 84, average 60.1; with amplification: 32 to 91, average 61.7; with polysomy: 37 to 76, average 58.7.
Recruitment	Participants recruited were female diagnosed with ER+ Luminal B breast cancer aged between 31 and 91 years old. Luminal B tumors were defined either by ER and HER2 co-expression or by high tumor grade in ER expressing malignancies.
Ethics oversight	The study was approved by the medical review board of the Medical University of Gdansk: Amplification of C11orf67 (AAMDC) as a potential prognostic factor in selected human malignancies (NKBBN/205/2017) and all patients gave written informed consent.

Note that full information on the approval of the study protocol must also be provided in the manuscript.
